# Synthesis of Aminooxy Glycoside Derivatives of the Outer Core Domain of *Pseudomonas aeruginosa* Lipopolysaccharide

**DOI:** 10.3389/fmolb.2021.750502

**Published:** 2021-11-08

**Authors:** Anshupriya Si, Steven J. Sucheck

**Affiliations:** Department of Chemistry and Biochemistry, University of Toledo, Toledo, OH, United States

**Keywords:** thioglycosides, vaccine, lipopolysaccharide, *Pseudomonas aeruginosa*, outer core, aminooxy glycosides

## Abstract

*Pseudomonas aeruginosa* is a highly prevalent gram-negative bacterium that is becoming more difficult to treat because of increasing antibiotic resistance. As chemotherapeutic treatment options diminish, there is an increased need for vaccines. However, the creation of an effective *P. aeruginosa* vaccine has been elusive despite intensive efforts. Thus, new paradigms for vaccine antigens should be explored to develop effective vaccines. In these studies, we have focused on the synthesis of two L-rhamnose–bearing epitopes common to glycoforms I and II of the outer core domain of *Pseudomonas aeruginosa* lipopolysaccharide, α-L-Rha-(1→6)-α-D-Glc-(1→4)-α-D-Gal*N*-(Ala)-α-aminooxy (**3**) and α-L-Rha-(1→3)-β-D-Glc-(1→3)-α-D-Gal*N*-(Ala)-α-aminooxy (**4**), respectively. The target trisaccharides were both prepared starting from a suitably protected galactosamine glycoside, followed by successive deprotection and glycosylation with suitably protected D-glucose and L-rhamnose thioglycosides. Global deprotection resulted in the formation of targets **3** and **4** in 22 and 35% yield each. Care was required to modify basic reaction conditions to avoid early deprotection of the *N*-oxysuccinamido group. In summary, trisaccharides related to the L-rhamnose–bearing epitopes common to glycoforms I and II of the outer core domain of *Pseudomonas aeruginosa* lipopolysaccharide have been prepared as their aminooxy glycosides. The latter are expected to be useful in chemoselective oxime-based bioconjugation reactions to form *Pseudomonas aeruginosa* vaccines.

## Introduction

**FIGURE 1:**
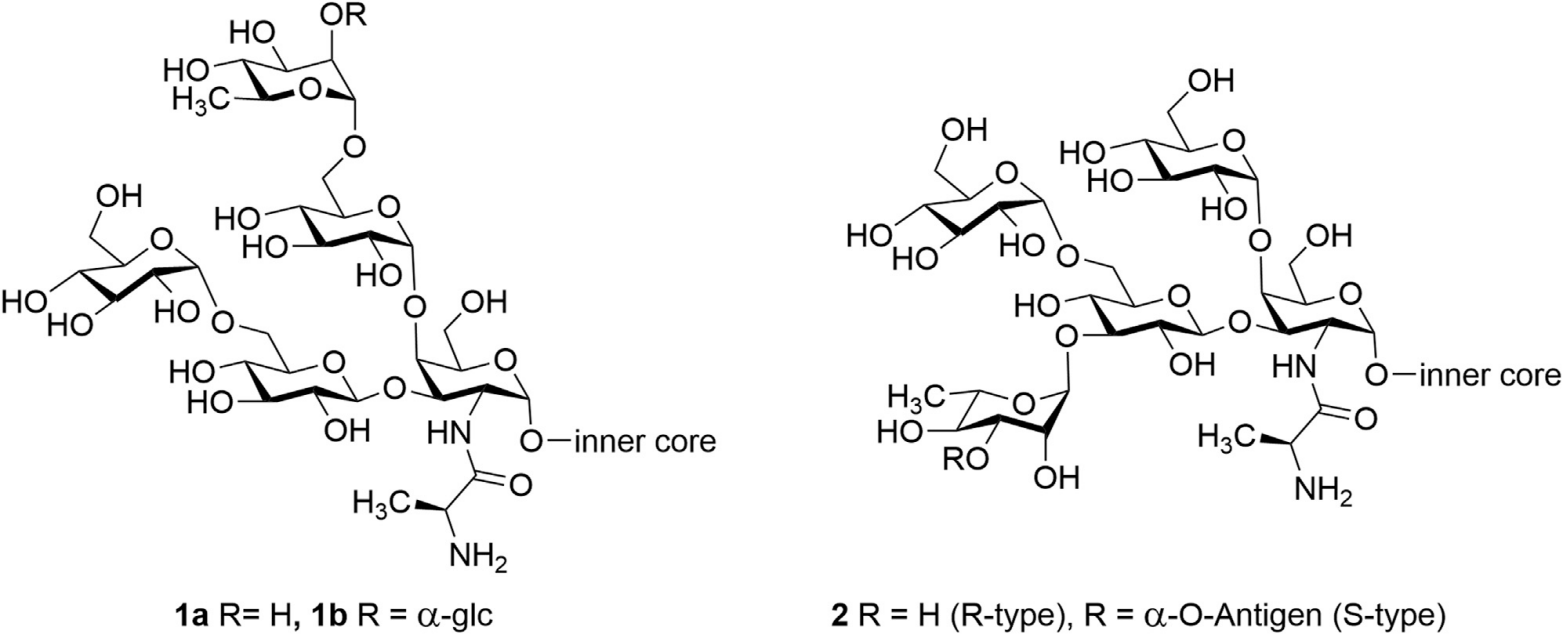
*P. aeruginosa* LPS outer core structure of glycoform I (**1a,b**) and glycoform II (**2**).

*Pseudomonas aeruginosa* is a widely distributed, encapsulated, gram-negative bacterium. In the early 1900s, it was recognized as a bacterial pathogen, and in the past 50 years, it has become one of the most concerning pathogens. It is reported that *P. aeruginosa* is the cause of 1 in 10 nosocomial infections associated with serious illness such as ventilator-associated pneumonia and various sepsis syndromes, and it has the highest mortality rate (37%) (Klevens et al., 2008; Lister et al., 2009). Most *P. aeruginosa* is resistant to at least one of the classes of antibiotics, and some *P. aeruginosa* is resistant to all the available antibiotics (Talbot et al., 2006; Pier, 2007). Therefore, vaccines are potential solutions to overcome the antimicrobial resistance (AMR) developed by *P. aeruginosa*. Lipopolysaccharide (LPS) is a complex glycolipid present on the outer layer of gram-negative bacteria. It plays a vital role as an essential virulence factor in the pathogenicity of *P. aeruginosa* strains and, hence, is a potential antigen target for a prophylactic vaccine (Pier, 2007). In a recent study, Liu et al. published that this bacterium could have one of twenty different O-polysaccharides as a part of its LPS (Liu et al., 1983; Liu and Wang, 1990). This is a proposed reason for inconsistent efficiency of some of the vaccines that were formulated based on isolated LPS as target antigens. Therefore, we have shifted our focus to synthetic outer core domains of the LPS that have been shown to react with protective monoclonal antibodies that promoted macrophage-mediated opsonophagocytosis against several serotypes and are also believed to target the rhamnose moiety and neighboring saccharides (Yokota et al., 1989; Terashima et al., 1991). It is noted that LPS is made up of O-polysaccharides linked to Lipid A *via* an intervening core oligosaccharide. Outer-membrane LPS consists of both smooth-type (S) LPS and rough-type (R) LPS based on the presence and absence of O-polysaccharides, respectively (Rocchetta et al., 1999; Raetz and Whitfield, 2002). Both the (S)-type laboratory strains and (R)-type clinical strains of *P. aeruginosa* share the presence of structurally similar outer-core glycoform I (**1**) and glycoform II (**2**) (Figure 1) consisting of one D-galactosamine (GalN) residue, three D-glucose residues, and one L-rhamnose residue. The GalN residue is acylated with an L-alanyl group in all LPSs (Sadovskaya et al., 1998; Sadovskaya et al., 2000; Knirel et al., 2001; Bystrova et al., 2002; Bystrova et al., 2003; Bystrova et al., 2004; Bystrova et al., 2006).

**FIGURE 2:**
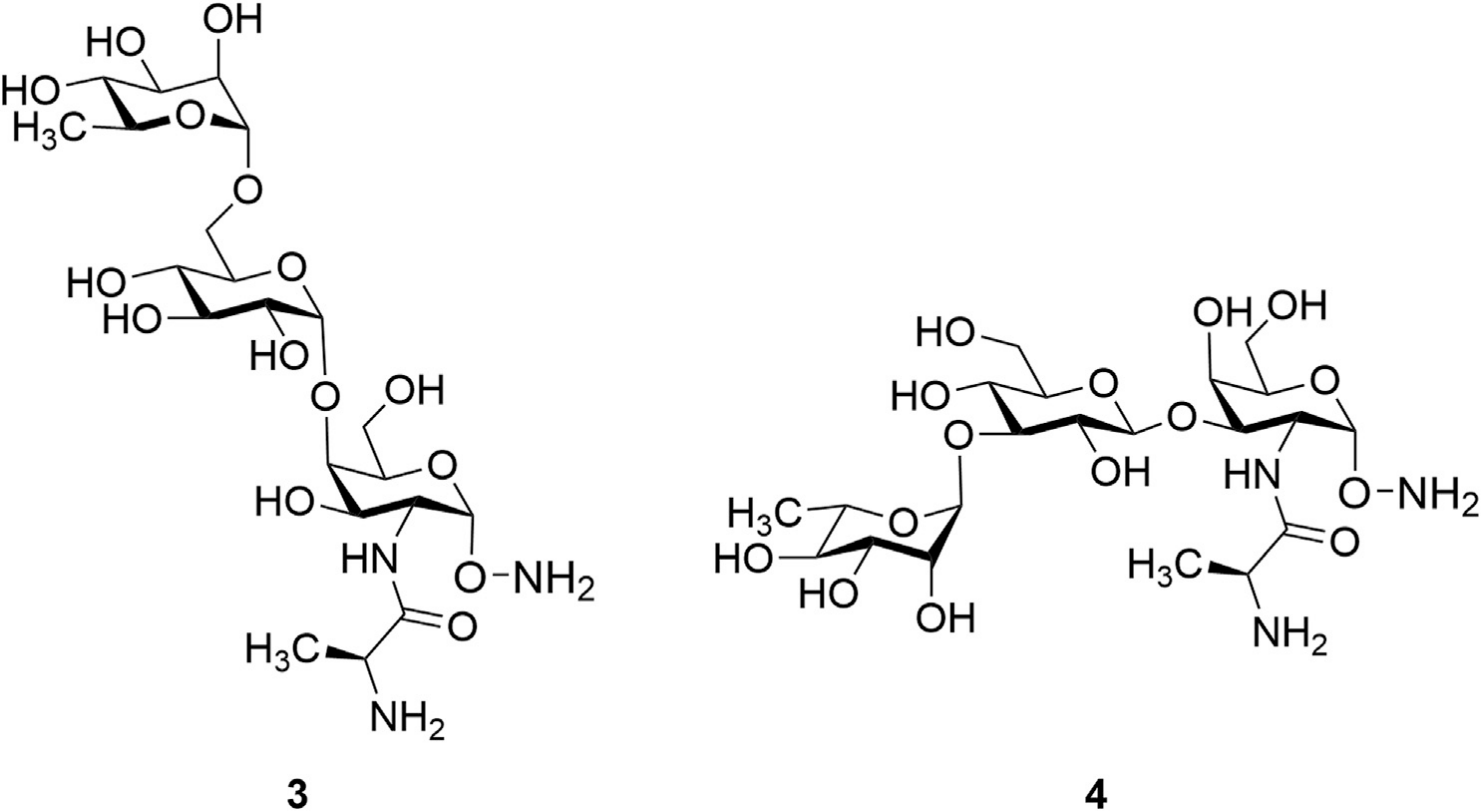
Two target trisaccharide fragments common to glycoform I and glycoform II, respectively.

Glycan epitopes common to both S-type and R-type LPS are potentially ideal antigen targets as they are less serotype dependent. However, isolation of these minimal epitopes from the biological source is not possible. Therefore, it is essential to develop synthetic strategies to obtain these materials for study. In this context, synthesis of two common trisaccharide fragments found within the outer core domain of *P. aeruginosa* related to glycoform I and glycoform II, noted as α-L-Rha-(1→6)-α-d-Glc-(1→4)-α-D-Gal*N*-(Ala)-α-aminooxy **(3)** and α-L-Rha-(1→3)-β-D-Glc-(1→3)-α-D-Gal*N*-(Ala)-α-aminooxy (**4**) (Figure 2), respectively, is reported herein. We have selected the succinimidyl group as the aminooxy precursor and introduced it at the preliminary stage of the synthetic strategies based on the previous reports (Marcaurelle et al., 2001; De Silva et al., 2009) for developing biologically stable oxime bonds for glycoconjugate vaccines. The use of *N*-hydroxysuccinimide (NHS) was essential for introducing the aminooxy (-ONH_2_) functionality which has already proved very useful in several reports (Bourgault et al., 2014; Ghosh and Andreana, 2014; Ghosh et al., 2016; Ghosh et al., 2020; Kleski et al., 2020). The presence of an aminooxy group at the reducing terminal of the trisaccharides provides a readily available form for conjugation to an appropriate carbonyl-modified carrier protein (Agten et al., 2016) without destroying the cyclic structure of the reducing end saccharide. The resulting glycoconjugates can then be evaluated in immunological experiments.

## Results and Discussion

Earlier syntheses of pentasaccharide and trisaccharide fragments of outer core domains corresponding to glycoforms I and II of *P. aeruginosa* containing a methoxy group and *tert*-butyldiphenylsilyl (TBDPS)-protected hydroquinone (TPH) as a multifunctional reducing-end capping group have been reported (Komarova et al., 2006; Komarova et al., 2008, 2012; Vartak et al., 2018). In these studies, we sought a route to an aminooxy glycoside that could be used in an oxime-based conjugation which would leave the unique L-Ala–modified galactosamine in its native state.

**FIGURE 3:**
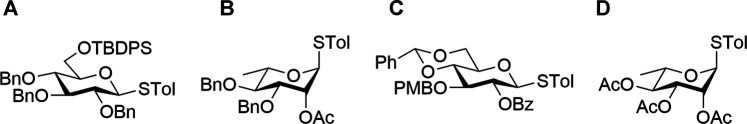
Thioglycoside donor building blocks **(A–D)**.

The target trisaccharide fragments of glycoforms I and II were synthesized as their α-aminooxy glycosides from suitably functionalized monosaccharides using stereoselective sequential glycosylations and functional group manipulations. A set of suitably functionalized donor thioglycoside building blocks, **A** (Tam and Lowary, 2010), **B** (Rajput and Mukhopadhyay, 2008), **C** (He et al., 2019), and **D** (Mukhopadhyay et al., 2004) (Figure 3), were prepared from the naturally available reducing sugars applying a number of reaction conditions reported earlier. D-(+)-glucose–based thioglycoside donors **A** and **C** were obtained from commercially available D-(+)-glucose. L-rhamnose–based glycosyl donors **B** and **D** were synthesized starting from L-rhamnose according to reported literature (Mukhopadhyay et al., 2004; Rajput and Mukhopadhyay, 2008; Tam and Lowary, 2010; He et al., 2019).

**SCHEME 1:**
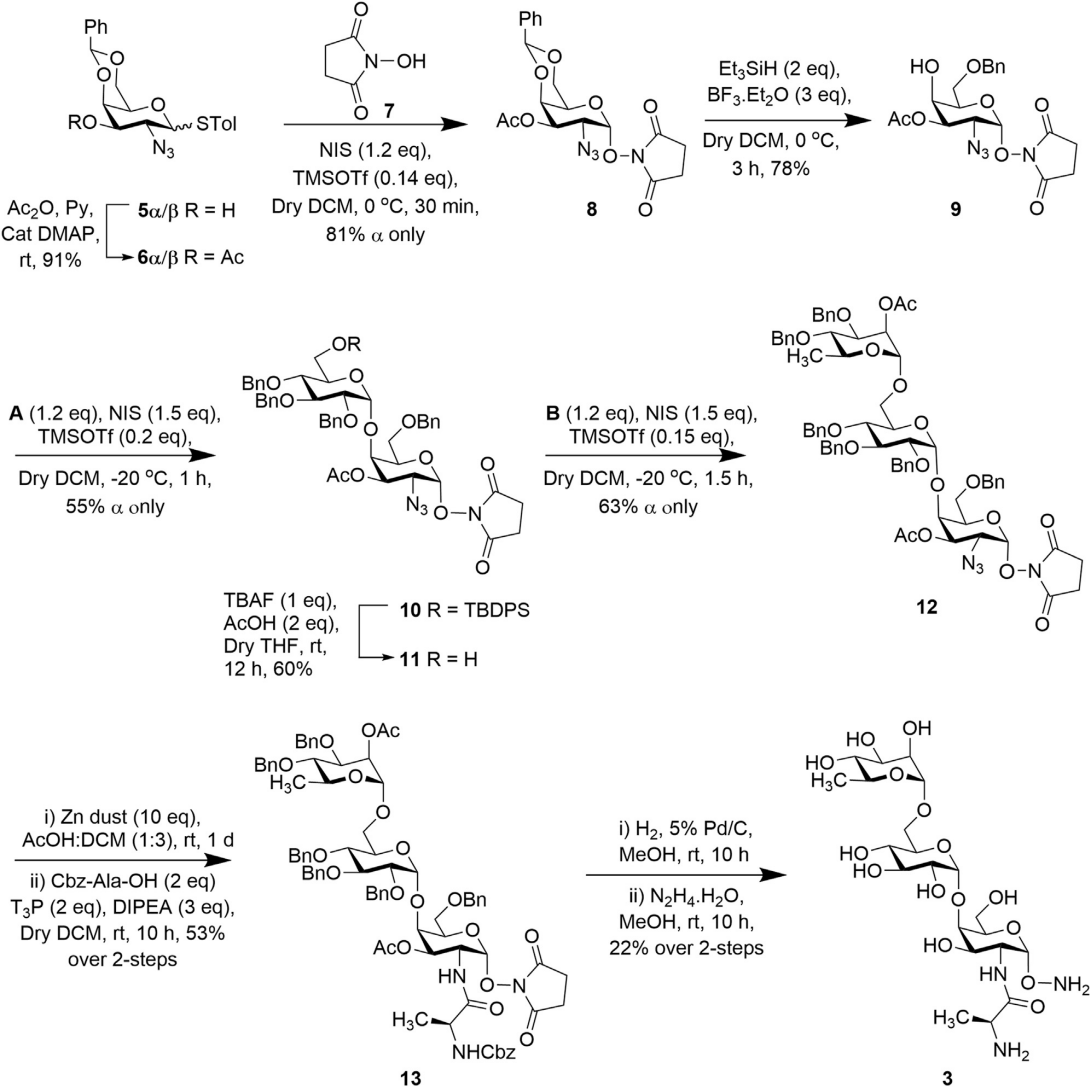
Synthesis of trisaccharide fragment target **3**.

Syntheses of the two trisaccharides, **3** and **4**, proceeded from common intermediate *p*-methylphenyl 2-azido-4,6-*O-*benzylidene-2-deoxy-1-thio-α/β-D-galactopyranoside (**5α/β**) (Scheme 1) which was prepared from tri-*O*-acetyl-D-galactal over six steps according to the reported procedure (Lemieux and Ratcliffe, 1979; Santra et al., 2012).

To access the target trisaccharide **3** of *P. aeruginosa* LPS, the 3-OH positions of both **5α/β** were acetylated using acetic anhydride and pyridine to obtain *p*-methylphenyl 2-azido-3-*O*-acetyl-4,6-*O-*benzylidene-2-deoxy-1-thio-α/β-D-galactopyranoside (**6α/β**) in 91% yield (Santra et al., 2012). Both the isomers were used as donors for stereoselective glycosylation with N-hydroxy succinimide (NHS) (**7**) in the presence of a combination of *N*-iodosuccinimide (NIS) and trimethylsilyl trifluoromethanesulfonate (TMSOTf) (Nicolaou et al., 1983; Konradsson et al., 1990; Veeneman et al., 1990) at 0°C to furnish compound **8** (exclusively α product) in 81% yield. NMR spectral analysis of this compound supported its formation; signals at [δ = 5.60 ppm (d, *J* = 3.7 Hz, 1H, H-1) in the ^1^H NMR and at 102.80 (C-1) in the ^13^C NMR spectra]. Compound **8** was subjected to regioselective reductive cleavage by treatment with borontrifluoride diethyletherate and triethylsilane (Et_3_SiH) (Debenham and Toone, 2000) at 0°C to selectively open the benzylidene ring to afford acceptor **9** containing a free C-4 hydroxyl in 78% yield (Scheme 1). Acceptor **9** was then coupled with thioglucoside donor **A** in the presence of a combination of NIS and TMSOTf (as before) in a mixed solvent (CH_2_Cl_2_-Et_2_O; 1:3) at −20°C for 40 min to furnish disaccharide **10** in 55% yield (α only) (Scheme 1). The newly formed glycosyl linkage in the compound was confirmed by its NMR spectral analysis: signals at δ = 5.60 (d, *J* = 3.8 Hz, 1H, H-1) and 4.91 ppm (brs, 1H, H-1′) in the ^1^H NMR and at 101.82 (C-1) and 99.94 ppm (C-1′) in the ^13^C NMR spectra. The TBDPS group was deprotected using 1 M tetra-*n*-butyl ammonium fluoride (TBAF) solution in THF and in the presence of a catalytic amount of acetic acid (AcOH) to afford disaccharide acceptor **11** in 60% yield. The compound was subsequently glycosylated with rhamnose-based thioglycoside donor **B** in the presence of a combination of NIS and TMSOTf (as before) at −20°C for 30 min to afford trisaccharide **12** in 63% yield (α only), which was confirmed from its spectral analysis: signals at δ = 5.64 (d, *J* = 3.8 Hz, 1H, H-1), 4.84 (brs, 1H, H-1′), and 4.67 ppm (brs, 1H, H-1″) in the ^1^H NMR and at 101.78 (C-1), 99.52 (C-1′), and 97.86 ppm (C-1″) in the ^13^C NMR spectra. The Cbz-alanine residue was then introduced by reducing the azide group of the trisaccharide **12** with Zn/AcOH, followed by coupling with Cbz-Ala-OH using propylphosphonic anhydride (T3P) as the coupling reagent and DIPEA to afford trisaccharide derivative **13** in 53% overall yield. Finally, the benzyl groups were removed by hydrogenation using 5% Pd/C followed by treatment with hydrazine hydrate for deprotection of the succinimide group and the remaining acetate groups to furnish the final product **3** (Scheme 1) in 22% yield. Spectral analysis of compound **3** unambiguously supported its formation [signals at δ 4.93 (brs, 1H, H-1″), 4.88 (brs, 1H, H-1), and 4.85 ppm (brs, 1H, H-1′) in the ^1^H NMR and at 100.37 (C-1′) and 100.22 ppm (C-1, C-1″) in the ^13^C NMR spectra].

**SCHEME 2:**
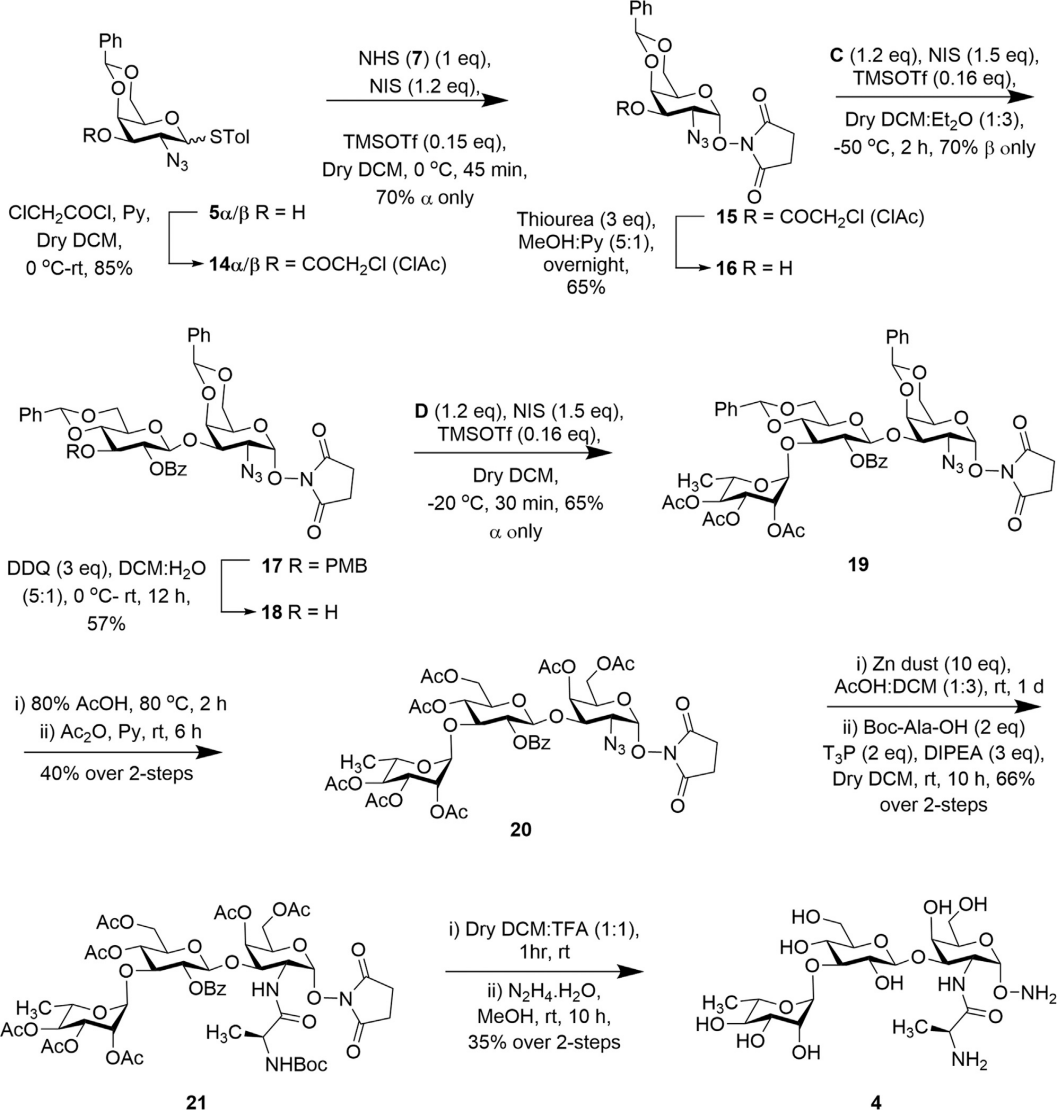
Synthesis of trisaccharide fragment target **4**.

To access the second trisaccharide common outer core fragment of *P. aeruginosa* LPS, we selected the chloroacetyl group (ClAc) for 3-OH protection inspired by previous uses (Jiaang et al., 2000; Bourgault et al., 2014). Treatment of both **5α/β** with chloroacetyl chloride and pyridine afforded *p*-methylphenyl 2-azido-4,6-*O*-benzylidene-3-*O*-chloroacetyl-2-deoxy-1-thiol-α/β-D-galactopyranoside (**14α/β**) in 85% yield. Both the compounds were then used for stereoselective 1,2-cis glycosylation with *N*-hydroxy succinimide (**7**) in the presence of NIS and TMSOTf (as before) at 0°C to furnish compound succinimidyl 2-azido-4,6-*O*-benzylidene-2-deoxy-α-D-galactopyranoside (**15**) in 70% yield. NMR spectral analysis of compound **15** supported its formation: signals at δ = 5.56 ppm (d, *J* = 3.7 Hz, 1H, H-1) in the ^1^H NMR and at 103.01 (C-1) in the ^13^C NMR spectra and it matches with the previously reported data (Bourgault et al., 2014). The chloroacetyl group was selectively removed (Masaki et al., 1968) by the treatment with a nonbasic nucleophile, thiourea, to afford the first monosaccharide acceptor **16** in 65% yield without affecting the base labile NHS group (Scheme 2).

With a reasonable amount of acceptor in hand, iodonium ion–mediated stereoselective 1,2-trans glycosylation was achieved with thioglucoside donor **C** in the presence of a combination of NIS and TMSOTf (as before) at −50°C to afford β (1 → 3) disaccharide **17** in 70% yield. Exclusive formation of compound **17** was confirmed from its spectral analysis: signals at δ = 5.53 (d, *J* = 3.7 Hz, 1H, H-1) and 4.93 ppm (d, *J* = 7.7 Hz, 1H, H-1′) in the ^1^H NMR and at 103.04 (C-1) and 102.83 ppm (C-1′) in the ^13^C NMR spectra. To build the target trisaccharide, oxidative removal of the *p*-methoxybenzyl (PMB) (Oikawa et al., 1982) group from compound **17** was achieved by the treatment with 2,3-dicholoro-5,6-dicyano-1,4-benzoquinone (DDQ) to give disaccharide acceptor **18** in 57% yield. Acceptor **18** and rhamnoside donor **D** were then coupled using the NIS:TMSOTf (as before) promoter system at −20°C to furnish the product **19** in 65% yield. From spectral analysis it was determined that the product was exclusively the desired trisaccharide without producing unwanted orthoester. The stereochemistry at the glycosidic linkages in compound **19** was confirmed from its spectral analysis: signals at δ = 5.52 (d, *J* = 3.7 Hz, 1H, H-1), 5.06 (d, *J* = 7.3 Hz, 1H, H-1′), and 4.83 ppm (brs, 1H, H-1″) in the ^1^H NMR and at 102.96 (C-1), 102.27 (C-1′), and 97.78 ppm (C-1″) in the ^13^C NMR spectra. The global deprotection was achieved by treatment of the compound **19** with 80% acetic acid at 80°C to remove the benzylidene acetal groups. This step was followed by acetylation with acetic anhydride and pyridine to afford trisaccharide **20** in 40% yield over two steps. The azido group was reduced to an amine by the treatment with Zn/AcOH and coupled with Boc-Ala-OH in the presence of T3P in one pot to obtain trisaccharide derivative **21** in 66% yield in two steps. Finally, trisaccharide was subjected to deprotection reactions including (a) removal of the Boc-group using trifluoroacetic acid (TFA) and (b) removal of the remaining acetyl groups and the replacement of the succinimidyl protecting group as the α-aminooxy group by the treatment with hydrazine monohydrate to afford the target trisaccharide **4** in 35% overall yield (Scheme 2). The formation of compound **4** was confirmed by spectroscopic analysis: signal δ 5.00 (brs, 1H, H-1″), 4.86 (d, *J* = 4.0 Hz, 1H, H-1), and 4.43 ppm (d, *J* = 8.0 Hz, 1H, H-1′) in the ^1^H NMR and at 103.50 (C-1′), 100.91 (C-1″), and 100.44 ppm (C-1) in the ^13^C NMR spectra.

Difficult-to-remove side products were generated during the removal of the succinimide group due to the use of excess hydrazine monohydrate (Renaudet and Dumy, 2004; Ghosh and Andreana, 2014). Furthermore, it was challenging to purify both deprotected compounds using only a C18 silica gel column. The problem was solved by passing the compounds through size exclusion chromatography (Bio-gel P-2) using water as the eluent. Fractions containing aminooxy sugars **3** and **4** (identified by TLC staining) were collected, frozen, and lyophilized. The resulting white solid was characterized by NMR and HRMS.

## Conclusion

In conclusion, oligosaccharide fragments corresponding to the outer core domain of *P. aeruginosa* LPS were synthesized in good yield using a sequential glycosylation approach. During the synthesis of the target molecules, similar reaction conditions were used in each of the intermediate glycosylation reaction steps to obtain excellent stereochemical outcomes. This report again presents the importance of the NHS group for the synthesis of aminooxy glycosides where the base-sensitive NHS group remains stable through multiple glycosylations, protecting group modifications, and deprotections. However, care was required to avoid basic conditions to maintain NHS stability. For example, the modification of TBAF with 2 equivalents of acetic acid was essential for NHS stability. Furthermore, the route to compound **3** was tolerant of carefully controlled hydrogenolysis to avoid reduction of the N-O bond. Formation of the aminooxy linkage at the reducing end is expected to afford a convenient handle for highly chemoselective oxime conjugation with an appropriately modified carrier protein.

## Experimental

### General Methods

All chemicals and solvents were purchased from Fisher Scientific, Acros Organics, Alfa Aesar, or Sigma-Aldrich. Reactions are carried out under an atmosphere of nitrogen using a nitrogen balloon. Solvents were dried using a solvent purification system by passing them through activated alumina and copper catalyst columns. Reactions were monitored by TLC (silica gel, f_254_) under UV light or by charring (5% H_2_SO_4_-MeOH), and the purification was performed by column chromatography on silica gel (230–400 mesh), C-18, and P-2 biogel using the solvent system specified; solvents were used without purification for chromatography. ^1^H NMR was recorded on a Bruker Avance III 600 MHz spectrometer using CDCl_3_ and D_2_O as an internal reference. ^13^C were recorded on a Bruker Avance III 600 MHz spectrometer using CDCl_3_ and D_2_O as the internal reference. High-resolution mass spectrometry was recorded on a Thermo LTQ XL Orbitrap instrument from the Ohio State University Mass Spectrometry Center.

### *p*-methylphenyl 2-azido-3-*O*-acetyl-4,6-*O-*benzylidene-2-deoxy-1-thio-α/β-D-galactopyranoside (6α/β)

Acetic anhydride (1.07 ml, 11.3 mmol) was added to *p*-methylphenyl 2-azido-4,6-*O*-benzylidene-2-deoxy-1-thio-α/β-D-galactopyranoside (**5α/β**) (1.50 g, 3.76 mmol) in pyridine (1.50 ml, 18.6 mmol) and the mixture was stirred at room temperature for 1 h. After completion of the reaction, the solvents were removed under reduced pressure and co-evaporated with toluene three times (3 × 10 ml). The reaction mixture was diluted with ethyl acetate and successively washed with satd. CuSO_4_ (2 × 100 ml), 1 N HCl (2 × 50 ml), and brine (2 × 100 ml) solution. The organic layer was collected, dried over anhydrous Na_2_SO_4_, and concentrated. The residue was purified by silica gel flash column chromatography to isolate the compound as white solid **6α/β**: yield 91% (1.50 g); silica gel TLC *R*_*f*_ = 0.65 (50% ethyl acetate: hexane). All the spectral data match with reported data (Santra et al., 2012).

### Succinimidyl 2-azido-3-*O*-acetate-4,6-*O*-benzylidene-2-deoxy-α-D-galactopyranoside (8)

Both **6α/β** compound (1.20 g, 2.72 mmol) and *N*-hydroxy succinimide **(7)** (0.31 g, 2.70 mmol) were mixed in a flame-dried round bottom flask and left under high vacuum overnight. To the dried reagents were added flame-dried 4-Å molecular sieves and 20 ml of anhydrous DCM. This mixture was then stirred at room temperature for 15 min. NIS (0.73 g, 3.26 mmol) was added to it, and the reaction mixture was cooled to 0°C. Subsequently, (70 μL, 0.38 mmol) TMSOTf was added. After 30 min, the reaction was diluted with DCM and filtered. The filtrate was washed with aq. sodium thiosulfate (2 × 50 ml), aq. sodium bicarbonate (2 × 50 ml), and brine (2 × 100 ml) solution. The organic layer was collected, dried over anhydrous Na_2_SO_4_, and concentrated. The residue was purified by silica gel flash column chromatography to isolate the compound as white solid **8**: yield 81% (0.95 g); silica gel TLC *R*_*f*_ = 0.20 (50% ethyl acetate: hexane). ^1^H NMR (600 MHz, CDCl_3_) δ 7.58–7.33 (Ar-H, 5H), 5.60 (d, *J* = 3.7 Hz, 1H, H-1), 5.56 (s, 1H, PhC*H*), 5.42 (dd, *J* = 11.4, 3.3 Hz, 1H, H-3), 4.72 (m, 1H, H-5), 4.56 (dd, *J* = 3.4, 1.2 Hz, 1H, H-4), 4.23–4.07 (m, 3H, H-2, H-6a, H-6b), 2.75 (s, 4H), 2.18 (s, 3H, COC*H*_3_). ^13^C NMR (151 MHz, CDCl_3_) δ 170.86 (2C), 170.29, 137.40–126.12 (Ar-C), 102.80, 100.78, 73.13, 68.88, 68.87, 64.65, 56.33, 25.47 (2C), 20.97. mass spectrum (HRMS), *m/z* = 455.1168 (M+Na)^+^, C_19_H_20_N_4_O_8_ requires 455.1173.

### Succinimidyl 2-azido-3-*O*-acetate-6-*O*-benzyl-2-deoxy-α-D-galactopyranoside (9)

To the solution of compound **8** (0.93 g, 2.14 mmol) in anhydrous DCM (25 ml) at 0°C, BF_3_.OEt_2_ (0.80 ml, 6.45 mmol) and triethylsilane (Et_3_SiH) (0.70 ml, 4.30 mmol) were added successively using a syringe. The reaction was stirred under N_2_ and monitored by TLC. After 3 h, the reaction was quenched with aq. sodium bicarbonate (2 × 50 ml) solution. The compound was extracted with DCM (2 × 50 ml). The organic layer was collected, dried over anhydrous Na_2_SO_4_, and purified by silica gel flash column chromatography to obtain the compound as white solid **9**: yield 78% (725 mg); silica gel TLC *R*_*f*_ = 0.15 (50% ethyl acetate: hexane). mass spectrum ^1^H NMR (600 MHz, CDCl_3_) δ 7.46–7.22 (Ar-H, 5H), 5.58 (d, *J* = 3.8 Hz, 1H, H-1), 5.35 (dd, *J* = 11.2, 2.8 Hz, 1H, H-3), 4.86 (t, *J* = 3.5 Hz, 1H, H-5), 4.64 (d, *J* = 11.7 Hz, 1H, PhC*H*), 4.52 (d, *J* = 11.7 Hz, 1H, PhC*H*), 4.46–4.39 (m, 1H, H-4), 4.21 (dd, *J* = 11.1, 3.9 Hz, 1H, H-2), 3.90–3.76 (m, 2H, H-6a, H-6b), 2.76 (s, 4H), 2.22 (s, 3H, COC*H*_3_). ^13^C NMR (151 MHz, CDCl_3_) δ 170.70 (2C), 170.07, 136.80–127.87 (Ar-C), 102.63, 74.08, 71.06, 70.48, 70.04, 69.19, 56.23, 25.47 (2C), 20.99 (HRMS), *m/z* = 457.1327 (M+Na)^+^, C_19_H_22_N_4_O_8_ requires 457.1330.

### Succinimidyl [2,3,4-tri-*O*-benzyl-6-tertbutyldiphenylsillyl-α-D-glucopyranosyl]-(1→4)-2-azido-3-*O*-acetate-6-*O*-benzyl-2-deoxy-α-D-galactopyranoside (10)

The acceptor **9** (0.70 g, 1.56 mmol) and thioglycoside donor **A** (1.50 g, 1.88 mmol) were dissolved in dry DCM (5 ml) and dry diethyl ether (15 ml) and stirred using 4-Å molecular sieves for 30 min. NIS (0.51 g, 2.26 mmol) and TMSOTf (50 μl, 0.27 mmol) were added successively to the reaction mixture at −20°C. The solution was then allowed to warm to room temperature. The reaction was stirred under N_2_ and observed to be completed after 1 h. The reaction was diluted with DCM (50 ml) and filtered. The filtrate was washed with aq. sodium thiosulfate (2 × 50 ml), aq. sodium bicarbonate (2 × 50 ml), and brine (2 × 50 ml) solution. The organic layer was collected and dried over anhydrous Na_2_SO_4_, and the residue was subjected to silica gel flash column chromatography to afford the compound as white solid **10**: yield 55% (0.98 g α only); silica gel TLC *R*_*f*_ = 0.50 (30% ethyl acetate: hexane). ^1^H NMR (600 MHz, CDCl_3_) δ 7.78–7.63 (Ar-H, 4H), 7.49–7.22 (Ar-H, 26H), 5.60 (d, *J* = 3.8 Hz, 1H, H-1), 5.35 (dd, *J* = 11.5, 2.7 Hz, 1H, H-3), 5.00 (d, *J* = 11.6 Hz, 1H, PhC*H*), 4.96 (d, *J* = 11.6 Hz, 1H, PhC*H*), 4.94–4.88 (m, 2H, H-1′, H-5), 4.80 (dd, *J* = 13.6, 11.1 Hz, 2H, 2 PhC*H*), 4.68 (d, *J* = 11.6 Hz, 1H, PhC*H*), 4.40 (d, *J* = 11.9 Hz, 1H, PhC*H*), 4.32 (d, *J* = 11.9 Hz, 1H, PhC*H*), 4.28–4.24 (m, 1H, H-4), 4.12 (dd, *J* = 11.4, 2.3 Hz, 1H, H-6b’), 4.08–3.95 (m, 2H, H-4′, H-5′), (dd, *J* = 11.5, 3.8 Hz, 1H, H-2), 3.93–3.81 (m, 3H, H-2′, H-6b, H-6a), 3.55 (ddd, *J* = 9.8, 4.9, 2.8 Hz, 2H, H-3′, H-6a’), 2.74–2.59 (m, 4H), 1.70 (s, 3H, COC*H*_3_), 1.10 (s, 9H, t-Bu-H). ^13^C NMR (151 MHz, CDCl_3_) δ 170.55 (2C), 170.01, 138.55–127.58 (Ar-C), 101.82, 99.94, 81.65, 80.91, 76.85, 75.79, 75.65, 75.47, 74.11, 72.83, 72.44, 71.28, 69.65, 67.53, 62.34, 56.88, 26.92, 25.43 (2C), 20.70, 19.45. mass spectrum (HRMS), *m/z* = 1127.4434 (M+Na)^+^, C_62_H_68_N_4_O_13_Si requires 1127.4552.

### Succinimidyl [2,3,4-tri-*O*-benzyl-α-D-glucopyranosyl]-(1→4)-2-azido-3-*O*-acetate-6-*O*-benzyl-2-deoxy α-D-galactopyranoside (11)

To the disaccharide **10** (0.97 g, 0.88 mmol) in THF (20 ml) was added acetic acid (0.10 ml, 1.75 mmol), followed by tetrabutylammonium fluoride (TBAF) (1.08 ml, 0.87 mmol, 1.0 M in THF). After stirring at room temperature for 12 h, the solution was washed with aq. sodium bicarbonate (2 × 50 ml) and brine (2 × 50 ml) solution and extracted with DCM (2 × 50 ml). The organic layer was collected and dried over anhydrous Na_2_SO_4_, and purification of the resulting residue was done by flash chromatography, yielding the disaccharide acceptor as white solid **11**: yield 60% (0.46 g); silica gel TLC *R*_*f*_ = 0.19 (30% ethyl acetate: hexane). ^1^H NMR (600 MHz, CDCl_3_) δ 7.29 (Ar-H, 20H), 5.62 (d, *J* = 3.9 Hz, 1H, H-1), 5.41 (dd, *J* = 11.6, 2.8 Hz, 1H, H-3), 5.03–4.85 (m, 5H, 3 PhC*H*, H-1′, H-5), 4.75 (d, *J* = 12.6 Hz, 1H, PhC*H*), 4.69 (d, *J* = 12.6 Hz, 1H, PhC*H*), 4.63 (d, *J* = 12.6 Hz, 1H, PhC*H*), 4.43 (d, *J* = 11.9 Hz, 1H, PhC*H*), 4.38–4.29 (m, 2H, PhC*H*, H-4), 4.08–3.95 (m, 3H, H-2, H-3′, H-5′), 3.88–3.73 (m, 3H, H-6a, H-6b, H-6b’), 3.59 (dd, *J* = 10.0, 9.0 Hz, 1H, H-4′), 3.54–3.46 (m, 2H, H-6a’, H-2′), 2.73–2.63 (m, 4H), 2.16 (s, 3H, COC*H*_3_). ^13^C NMR (151 MHz, CDCl_3_) δ 170.60 (2C), 170.13, 138.49–127.55 (Ar-C), 101.85, 99.38, 81.48, 80.27, 77.62, 75.57, 75.36, 75.17, 74.06, 72.89, 71.70, 71.00, 69.76, 67.23, 61.82, 56.97, 25.44 (2C), 21.10. mass spectrum (HRMS), *m/z* = 889.3259 (M+Na)^+^, C_46_H_50_N_4_O_13_ requires 889.3374.

### Succinimidyl [2-*O*-acetyl-3,4-di-*O*-benzyl-α-L-rhamnopyranosyl]-(1→6)-[2,3,4-tri-*O*-benzyl-α-D-glucopyranosyl]-(1→4)-2-azido-3-*O*-acetate-6-*O*-benzyl-2-deoxy-α-D-galactopyranoside (12)

Disaccharide acceptor **11** (0.45 g, 0.52 mmol) and rhamnose thioglycoside donor **B** (0.30 g, 0.62 mmol) were dried together in high vacuum overnight. The compounds were dissolved in dry DCM (20 ml), followed by the addition of 4-Å molecular sieves, and stirred for 30 min. The solution temperature was lowered to −20°C, and NIS (0.17 g, 0.78 mmol) and TMSOTf (15 μL, 0.08 mmol) were added. The reaction was monitored by TLC and appeared complete after 1.5 h. The reaction temperature was raised to 0°C. After completion, the reaction was diluted with DCM (50 ml), filtered, and washed with aq. sodium thiosulfate (2 × 30 ml), aq. sodium bicarbonate (2 × 30 ml), and brine (2 × 50 ml) solution. The organic layer was collected, dried over anhydrous Na_2_SO_4_, and concentrated. The residue was purified by silica gel flash column chromatography to isolate the compound as white solid **12**: yield 63% (0.40 g, α only); silica gel TLC *R*_*f*_ = 0.45 (30% ethyl acetate: hexane). ^1^H NMR (600 MHz, CDCl_3_) δ 7.44–7.17 (Ar-H, 30H), 5.64 (d, *J* = 3.8 Hz, 1H, H-1), 5.42 (ddd, *J* = 11.6, 2.7, 1.5 Hz, 1H, H-3), 5.27–5.24 (m, 1H, H-2″), 5.01–4.87 (m, 5H, 4 PhC*H*, H-5), 4.84 (brs, 1H, H-1′), 4.73 (dt, *J* = 11.7, 1.8 Hz, 2H, 2 PhC*H*), 4.67 (brs, 1H, H-1″), 4.65–4.56 (m, 4H, 4 PhC*H*), 4.38–4.31 (m, 3H, 2 PhC*H*, H-4), 4.04–3.95 (m, 3H, H-5″, H-4′, H-2), 3.93 (ddd, *J* = 9.4, 3.5, 1.5 Hz, 1H, H-3″), 3.83–3.76 (m, 3H, H-3′, H-6a, H-6b), 3.69–3.64 (m, 1H, H-6a’), 3.63–3.55 (m, 2H, H-5′, H-6b’), 3.48–3.42 (m, 2H, H-2′, H-4″), 2.76–2.60 (s, 4H), 2.14 (s, 6H, 2 COC*H*_3_), 1.30 (d, *J* = 6.2 Hz, 3H, Rha-C*H*_3_). ^13^C NMR (151 MHz, CDCl_3_) δ 170.56 (2C), 170.29, 169.99, 138.51–127.52 (Ar-C), 101.78, 99.52, 97.87, 81.50, 80.53, 80.06, 77.63, 77.47, 75.70, 75.65, 75.60, 75.42, 73.95, 72.77, 71.97, 71.25, 70.80, 69.74, 69.21, 67.75, 67.69, 66.00, 56.99, 25.43 (2C), 21.16, 21.10, 17.93. mass spectrum (HRMS), *m/z* = 1257.4881 (M+Na)^+^, C_68_H_74_N_4_O_18_ requires 1257.4998.

### Succinimidyl [2-*O*-acetyl-3,4-di-*O*-benzyl-α-L-rhamnopyranosyl]-(1→6)-[2,3,4-tri-*O*-benzyl-α-D-glucopyranosyl]-(1→4)-2-*N*-benzyloxycarbonylalanine-3-*O*-acetate-6-*O*-benzyl-2-deoxy-α-D-galactopyranoside (13)

To the solution of trisaccharide **12** (0.38 g, 0.30 mmol) in dry DCM:AcOH (3:1, 12 ml), zinc dust (100 mg, 1.53 mmol) was added, and the reaction was stirred under N_2_ at room temperature. After 1 day, the reaction was observed to be complete by TLC. The reaction was diluted with DCM (25 ml) and washed with aq. sodium bicarbonate (2 × 30 ml) and brine (2 × 30 ml). The organic layer was separated, dried over anhydrous Na_2_SO_4_, and evaporated. The residue was dried and used for the next reaction without further purification. To the solution of the residue in dry DCM, Cbz-Ala-OH (0.14 g, 0.61 mmol), T_3_P (0.18 ml, 0.61 mmol), and DIPEA (0.16 ml, 0.92 mmol) were added successively at 0°C. The solution was stirred under a N_2_ atmosphere and allowed to warm to room temperature. The reaction appeared complete after 12 h. The reaction was diluted with DCM (30 ml) and washed with 1N HCl (2 × 15 ml), followed by aq. sodium bicarbonate (2 × 30 ml). The organic layer was collected and purified by silica gel flash column chromatography to obtain the compound as fluffy white solid **13**: yield 53% over two steps (0.23 g); silica gel TLC *R*_*f*_ = 0.20 (50% ethyl acetate: hexane). ^1^H NMR (600 MHz, CDCl_3_) δ 7.47–7.12 (m, 36H), 6.66 (d, *J* = 9.5 Hz, 1H, N*H*), 5.38 (ddd, *J* = 11.6, 2.7, 1.5 Hz, 1H, H-3), 5.35 (d, *J* = 3.8 Hz, 1H, H-1), 5.26 (m, 1H, H-2″), 5.14 (brs, 2H, PhC*H*_2_), 5.02–4.84 (m, 6H, 4 PhC*H*, H-5, H-1″), 4.81–4.67 (m, 3H, 2 PhC*H*, H-2), 4.67–4.53 (m, 7H, 6 PhC*H*, H-1′), 4.45 (d, *J* = 12.0 Hz, 1H, PhC*H*), 4.41–4.29 (m, 2H, PhC*H*, C*H*), 4.30–4.19 (m, 2H, H-4, H-5′), 4.10 (t, *J* = 9.5 Hz, 1H, H-3′), 3.99–3.86 (m, 2H, H-3″, H-6a), 3.86–3.76 (m, 2H, H-5″, H-6a’), 3.70 (dd, *J* = 11.1, 3.6 Hz, 1H, H-6b’), 3.59 (t, *J* = 9.6 Hz, 1H, H-4′), 3.57–3.37 (m, 3H, H-6b, H-4″, H-2′), 2.67 (s, 4H), 2.10 (2 s, 6H, 2 COC*H*_3_), 1.45 (d, *J* = 7.1 Hz, 3H, Ala-C*H*_3_), 1.30 (d, *J* = 6.2 Hz, 3H, Rha-C*H*_3_). ^13^C NMR (151 MHz, CDCl_3_) δ 172.61 (2C), 170.73, 170.26, 138.72–127.50, 103.92, 98.85, 97.77, 81.66, 80.50, 80.14, 77.75, 77.71, 75.69, 75.60, 75.07, 74.13, 73.90, 72.82, 71.90, 71.36, 70.57, 69.13, 68.48, 67.60, 67.13, 66.08, 50.94, 47.94, 25.40 (2C), 21.14, 21.07, 18.17, 17.95. mass spectrum (HRMS), *m/z* = 1436.5712 (M+Na)^+^, C_79_H_87_N_3_O_21_ requires 1436.5832.

### Aminooxy [α-L-rhamnopyranosyl]-(1→6)-[α-D-glucopyranosyl]-(1→4)-2-*N*-alanine-2-deoxy-α-D-galactopyranoside (3)

To a solution of compound **13** (0.21 g, 0.15 mmol) in dry MeOH (10 ml) was added 25 mg of Pd/C (5 wt%). The reaction was stirred for 6 h under 1 atm of H_2._ The reaction was observed to be complete by TLC and ESI-MS. The reaction was diluted with MeOH (15 ml) and filtered through Celite 545. The filtrate was evaporated and dried and used for the next reaction without further purification. The residue (0.06 g, 0.08 mmol) was dissolved in methanol (3 ml), and then hydrazine hydrate (0.08 ml, 1.60 mmol) was added and the reaction was stirred for 10 h. The reaction mixture was then concentrated to dryness. This residue was dissolved in a minimal amount of water and purified using P-2 biogel with water as the eluent (collecting ∼0.5 ml fractions), to provide white solid **3**: yield 22% (10 mg); silica gel TLC *R*_*f*_ = 0.20 (50% methanol: DCM). ^1^H NMR (600 MHz, D_2_O) δ ^1^H NMR (600 MHz, D_2_O) δ 4.93 (brs, 1H, H-1″), 4.88 (brs, 1H, H-1), 4.85 (brs, 1H, H-1′), 4.10–4.02 (m, 2H, H-3, H-5), 3.97–3.93 (m, 3H, H-2, H-5″, H-3″), 3.86–3.82 (m, 2H, H-5′, H-4′), 3.70–3.66 (m, 5H, H-4″, C*H*, H-2′, H-3′, H-2″), 3.50–3.46 (m, 2H, H-6a, H-6b), 3.37–3.30 (m, 3H, H-4, H-6a’, H-6b’), 1.45 (d, *J* = 7.1 Hz, 3H, Ala-C*H*_3_), 1.17 (d, *J* = 6.3 Hz, 3H, Rha-C*H*_3_). ^13^C NMR (151 MHz, D_2_O) δ 171.89, 100.37, 100.22 (2C), 72.50, 71.92, 71.60, 71.24, 70.99, 70.12, 69.88, 69.23, 68.50, 67.12, 65.95, 60.29 (2C), 49.21 (2C), 16.50 (2 C). Data for. mass spectrum (HRMS), *m/z* = 574.2451 (M + H)^+^, C_21_H_39_N_3_O_15_ requires 574.2381.

### *p*-Methylphenyl 2-azido-4,6-*O*-benzylidene-3-*O*-chloroacetyl-2-deoxy-1-thiol-α/β-D-galactopyranoside (14α/β)

A solution of the compound *p*-methylphenyl 2-azido-4,6-*O-*benzylidene-2-deoxy-1-thio-α/β-D-galactopyranoside (**5α/β**) (Santra et al., 2012) (1.50 g, 3.75 mmol) in dry DCM (20 ml) and pyridine (0.30 ml, 3.75 mmol) was cooled to 0°C. To the cooled reaction mixture was added chloroacetyl chloride (0.36 ml, 4.52 mmol) using a syringe, and the reaction mixture was stirred for another 2 h at room temperature. After completion, the reaction mixture was diluted with DCM (50 ml). The organic layer was washed successively with satd. sodium bicarbonate (2 × 100 ml) and brine (2 × 100 ml) solution. The organic layer was collected, dried over anhydrous Na_2_SO_4_, and concentrated. The residue was purified by silica gel flash column chromatography to isolate the compound as white solid **14α/β**: yield 85% (1.51 g); silica gel TLC *R*_*f*_ = 0.60 (50% ethyl acetate: hexane). All the spectral data match with reported data (Zhang et al., 1999; Sarkar et al., 2003).

### Succinimidyl 2-azido-4,6-*O*-benzylidene-3-*O*-chloroacetyl-2-deoxy-α-D-galactopyranoside (15)

Both the **14α/β** compound (1.40 g, 2.94 mmol) and *N*-hydroxy succinimide (**7**) (0.34 g, 2.91 mmol) were mixed in a flame-dried round bottom flask and left under high vacuum overnight. To the dried reagents were added flame-dried 4-Å molecular sieves and 20 ml of anhydrous DCM. This mixture was then stirred at room temperature for 15 min. NIS (0.78 g, 3.50 mmol) was added to it, and the reaction mixture was cooled to 0°C. Subsequently, (80 μL, 0.44 mmol) TMSOTf was added. After 45 min, the reaction was diluted with DCM (50 ml), filtered, and washed with aq. sodium thiosulfate (2 × 50 ml), aq. sodium bicarbonate (2 × 50 ml), and brine (2 × 100 ml) solution. The organic layer was collected, dried over anhydrous Na_2_SO_4_, and concentrated. The residue was purified by silica gel flash column chromatography to isolate the compound as white solid **15**: yield 70% (0.96 g); silica gel TLC *R*_*f*_ = 0.30 (50% ethyl acetate: hexane). ^1^H NMR (600 MHz, CDCl_3_) δ 7.49–7.40 (Ar-H, 5H), 5.64 (t, *J* = 2.7 Hz, 1H, H-1), 5.58 (s, 1H, PhC*H*), 5.52–5.42 (m, 1H, H-5), 4.75 (t, *J* = 1.7, 1.7 Hz, 1H, H-3), 4.62 (dt, *J* = 3.3, 1.6 Hz, 1H, H-4), 4.27–4.17 (m, 4H, H-2, H-6a, COC*H*_2_), 4.11 (dt, *J* = 12.8, 1.9 Hz, 1H, H-6b), 2.80 (s, 4H). ^13^C NMR (151 MHz, CDCl_3_) δ 170.72, 166.76, 137.18, 129.30, 128.33, 126.08, 102.71, 100.82, 72.77, 70.78, 68.82, 64.54, 56.30, 40.65, 25.48. mass spectrum (HRMS), *m/z* = 498.0857 (M+Na)^+^, C_22_H_22_ClN_3_O_5_S requires 498.0784.

### Succinimidyl 2-azido-4,6-*O*-benzylidene-2-deoxy-α-D-galactopyranoside (16)

To a solution of compound **15** (0.93 g, 1.99 mmol) in MeOH:pyridine (90 ml, 5:1) was added thiourea (0.45 g, 5.98 mmol), and this reaction mixture was stirred overnight at room temperature. After completion of the reaction as shown in TLC, the reaction mixture was concentrated and co-evaporated with toluene two times. The residue was diluted with DCM (50 ml) and successively washed with aq. sodium bicarbonate (2 × 100 ml) and brine (2 × 100 ml) solution. The organic layer was collected, dried over anhydrous Na_2_SO_4_, and concentrated. The residue was purified by silica gel flash column chromatography to isolate the compound as white solid **16**: yield 65% (0.51 g); silica gel TLC *R*_*f*_ = 0.25 (50% ethyl acetate: hexane). ^1^H NMR (600 MHz, CDCl_3_) δ 7.54–7.46 (Ar-H, 2H), 7.46–7.37 (Ar-H, 4H), 5.61 (s, 1H, PhC*H*), 5.57 (d, *J* = 3.7 Hz, 1H, H-1), 4.69 (s, 1H, H-5), 4.39 (d, *J* = 3.7 Hz, 1H, H-4), 4.28 (d, *J* = 3.6 Hz, 1H, H-3), 4.21 (dd, *J* = 12.9, 1.3 Hz, 1H, H-6a), 4.10 (dd, *J* = 12.9, 1.6 Hz, 1H, H-6b), 3.88 (dd, *J* = 11.0, 3.5 Hz, 1H, H-2), 2.76 (s, 4H). ^13^C NMR (151 MHz, CDCl_3_) δ 170.89, 137.20, 129.49, 128.42, 126.22, 103.01, 101.29, 77.28, 77.07, 76.85, 75.07, 68.96, 67.28, 64.85, 59.76, 25.48. All the spectral data match with reported data (Bourgault et al., 2014).

### Succinimidyl [2-*O*-benzoyl-4,6-*O*-benzylidene-3-*O*-(p-methoxy)benzyl-β-D-glucopyranosyl]-(1→3)-2-azido-4,6-*O*-benzylidene-2-deoxy-α-D-galactopyranoside (17)

The acceptor **16** (0.50 g, 1.28 mmol) and thioglycoside donor **C** (0.92 g, 1.53 mmol) were dissolved in dry DCM (15 ml) and stirred using 4-Å molecular sieves for 10 min. NIS (0.42 g, 1.88 mmol) and TMSOTf (40 μL, 0.21 mmol) were added to the reaction at −50°C. The solution was then allowed to warm to −30°C. The reaction was stirred under N_2_ and observed to be complete after 2 h. The reaction was diluted with DCM (30 ml) and filtered. The filtrate was washed with aq. sodium thiosulfate (2 × 30 ml), aq. sodium bicarbonate (2 × 30 ml), and brine (2 × 50 ml) solution. The organic layer was collected and dried over anhydrous Na_2_SO_4_, and the resulting residue was subjected to silica gel flash column chromatography to afford the compound as white solid **17**: yield 70% (0.78 g β only); silica gel TLC *R*_*f*_ = 0.50 (50% ethyl acetate: hexane). ^1^H NMR (600 MHz, CDCl_3_) δ 8.03–7.97 (ArH, 2H), 7.57–7.28 (ArH, 22H), 7.06–6.99 (ArH, 2H), 6.60–6.55 (ArH, 2H), 5.56 (s, 1H, PhC*H*), 5.57 (s, 1H, PhC*H*), 5.53 (d, *J* = 3.7 Hz, 1H, H-1), 5.33 (t, *J* = 8.2, 8.2 Hz, 1H, H-2′), 4.93 (d, *J* = 7.7 Hz, 1H, H-1′), 4.72 (d, *J* = 11.8 Hz, 1H, PhC*H*), 4.62–4.54 (m, 2H, PhC*H*, H-5), 4.45–4.37 (m, 2H, H-4, H-5′), 4.19–4.12 (m, 2H, H-6a, H-3), 4.03 (ddd, *J* = 15.0, 11.9, 2.7 Hz, 2H, H-6b, H-2), 3.89–3.84 (m, 3H, H-6a’, H-4′, H-3′), 3.67 (s, 3H), 3.57–3.52 (m, 1H, H-6b’), 2.73 (s, 4H). ^13^C NMR (151 MHz, CDCl_3_) δ 170.94 (2C), 165.06, 159.04, 137.35–126.05 (Ar-C) 113.52, 103.04, 102.83, 101.29, 100.71, 81.18, 75.54, 74.36, 73.55, 73.09, 68.79, 68.63, 66.40, 65.19, 57.87, 55.12, 25.46 (2C). mass spectrum (HRMS), *m/z* = 887.2742 (M+Na)^+^, C_45_H_44_N_4_O_14_ requires 887.2746.

### Succinimidyl [2-*O*-benzoyl-4,6-*O*-benzylidene-β-D-glucopyranosyl]-(1→3)-2-azido-4,6-*O*-benzylidene-2-deoxy-α-D-galactopyranoside (18)

The solution of disaccharide **17** (0.76 g, 0.88 mmol) in DCM–H_2_O (40 ml, 5:1) was cooled to 0°C. To the cooled reaction mixture was added 2,3-dichloro-5,6-dicyano-1,4-benzoquinone (DDQ) (0.60 g, 2.63 mmol). The mixture was heated to room temperature and was stirred for 12 h. Then it was quenched with saturated aq. sodium bicarbonate (2 × 50 ml) and brine (2 × 50 ml), extracted with DCM, and dried over anhydrous Na_2_SO_4_, and the solvent was concentrated in vacuum. The residue was subjected to silica gel flash column chromatography to afford the compound as white solid **18**: yield 57% (0.37 g); silica gel TLC *R*_*f*_ = 0.40 (50% ethyl acetate: hexane). ^1^H NMR (600 MHz, CDCl_3_) δ 8.18–8.10 (Ar-H, 2H), 7.59–7.36 (Ar-H, 18H), 5.61–5.54 (m, 3H, 2 PhC*H*, H-1), 5.31 (dd, *J* = 8.9, 7.7 Hz, 1H, H-2′), 5.07 (d, *J* = 7.7 Hz, 1H, H-1′), 4.62 (brs, *J* = 1.3 Hz, 1H, H-5), 4.48–4.40 (m, 2H, H-4, H-6a’), 4.24 (dd, *J* = 11.1, 3.1 Hz, 1H, H-3), (4.22–4.07 (m, 4H, H-6a, H-6b, H-2, H-3′), 3.87 (t, *J* = 10.3 Hz, 1H, H-6b’), 3.77 (t, *J* = 9.4 Hz, 1H, H-4′), 3.61 (d, *J* = 4.9 Hz, 1H, H-5′), 2.76 (s, 4H). ^13^C NMR (151 MHz, CDCl_3_) δ 170.88 (2C), 166.15, 137.39, 136.80, 133.41, 129.93, 129.02, 128.48, 128.40, 128.21, 126.32, 126.12, 102.99, 102.53, 101.99, 100.69, 80.61, 77.26, 77.05, 76.84, 75.60, 74.81, 74.29, 72.70, 68.78, 68.55, 66.28, 65.23, 60.44, 57.99, 25.47 (2C). mass spectrum (HRMS), *m/z* = 767.2166 (M+Na)^+^, C_37_H_36_N_4_O_13_ requires 767.2171.

### Succinimidyl [2,3,4-tri-*O*-acetyl-α-L-rhamnopyranosyl]-(1→3)-[2-*O*-benzoyl-4,6-*O*-benzylidene-β-D-glucopyranosyl]-(1→3)-2-azido-4,6-*O*-benzylidene-2-deoxy-α-D-galactopyranoside (19)

Disaccharide acceptor **18** (0.35 g, 0.47 mmol) and donor **D** (0.22 g, 0.56 mmol) were dried together in high vacuum overnight. The compounds were dissolved in dry DCM (10 ml), followed by the addition of 4-Å molecular sieves, and stirred for 30 min. The solution temperature was lowered to −20°C, and NIS (0.15 g, 0.67 mmol) and TMSOTf (12 μL, 0.06 mmol) were added. The reaction was monitored by TLC and appeared complete after 30 min. The reaction temperature was raised to 0°C. After completion, the reaction was diluted with DCM (50 ml), filtered, and washed with aq. sodium thiosulfate (2 × 30 ml), aq. sodium bicarbonate (2 × 30 ml), and brine (2 × 30 ml) solution. The organic layer was collected, dried over anhydrous Na_2_SO_4_, and concentrated. The residue was purified by silica gel flash column chromatography to isolate the compound as white solid **19**: yield 65% (0.31 g); silica gel TLC *R*_*f*_ = 0.30 (50% ethyl acetate: hexane). ^1^H NMR (600 MHz, CDCl_3_) δ 8.13–7.96 (Ar-H, 2H), 7.46 (Ar-H, 10H), 5.59 (s, 1H, PhC*H*), 5.52 (d, *J* = 3.7 Hz, 1H, H-1), 5.50 (s, 1H, PhC*H*), 5.44 (dd, *J* = 8.2, 7.4 Hz, 1H, H-2′), 5.27 (dd, *J* = 10.1, 3.6 Hz, 1H, H-3″), 5.08 (dd, *J* = 3.6, 1.7 Hz, 1H, H-2″), 5.06 (d, *J* = 7.3 Hz, 1H, H-1′), 4.87 (t, *J* = 10.0 Hz, 1H, H-4″), 4.83 (brs, 1H, H-1″), 4.62–4.60 (m, 1H, H-5), 4.47 (dd, *J* = 3.3, 1.2 Hz, 1H, H-4), 4.44 (dd, *J* = 10.5, 4.9 Hz, 1H, H-6a’), 4.22 (dd, *J* = 11.0, 3.2 Hz, 1H, H-3), 4.19–4.05 (m, 5H, H-5″, H-3′, H-2, H-6a, H-6b), 3.92 (t, *J* = 9.4 Hz, 1H, H-4′), 3.85 (t, *J* = 10.3 Hz, 1H, H-6b’), 3.66 (td, *J* = 9.8, 5.0 Hz, 1H, H-5′), 2.74 (s, 4H), 1.95 (2s, 6H, 2COC*H*_3_), 1.82 (s, 3H, COC*H*_3_), 0.75 (d, *J* = 6.2 Hz, 3H, Rha-C*H*_3_). ^13^C NMR (151 MHz, CDCl_3_) δ 170.90 (2C), 169.91, 169.86, 169.07, 164.69, 137.37-126.11 (Ar-C), 102.96, 102.27, 101.81, 100.58, 97.78, 78.43, 77.29, 77.08, 76.87, 76.22, 75.45, 74.43, 74.16, 71.04, 69.36, 68.81, 68.73, 68.65, 66.61, 66.36, 65.15, 57.97, 25.45 (2C), 20.75, 20.68, 20.47, 17.30. mass spectrum (HRMS), *m/z* = 1039.3059 (M+Na)^+^, C_49_H_52_N_4_O_20_ requires 1039.3067.

### Succinimidyl [2,3,4-tri-*O*-acetyl-α-L-rhamno-pyranosyl]-(1→3)-[4,6-*O*-acetyl-2-*O*-benzoyl-β-D-glucopyranosyl]-(1→3)-2-azido-4,6-*O*-acetyl-2-deoxy-α-D-galactopyranoside (20)

The solution of compound **19** (0.30 g, 0.30 mmol) in 80% acetic acid (50 ml) was stirred at 80°C for 2 h, and the solvents were evaporated and co-evaporated with toluene (2 × 10 ml). To the solution of the crude compound in pyridine (2 ml) was added acetic anhydride (1 ml), and the reaction mixture was stirred at room temperature for 6 h. The solvents were removed under reduced pressure to yield a residue which was purified by silica gel flash column chromatography to isolate pure trisaccharide as white solid **20**: yield 40% over two steps (0.12 g); silica gel TLC *R*_*f*_ = 0.15 (50% ethyl acetate: hexane). ^1^H NMR (600 MHz, CDCl_3_) δ 8.12–7.97 (Ar*-*H, 2H), 7.61–7.43 (Ar-H, 3H), 5.62 (dd, *J* = 3.4, 1.3 Hz, 1H, H-4), 5.45 (d, *J* = 3.9 Hz, 1H, H-1), 5.30 (dd, *J* = 9.5, 7.8 Hz, 1H, H-2′), 5.18 (t, *J* = 9.6 Hz, 1H, H-4′), 5.14–5.09 (m, 1H, H-2″), 4.92–4.84 (m, 5H, H-3, H-5, H-3″, H-1′, H-1″), 4.32 (dd, *J* = 11.8, 4.6 Hz, 1H, H-6a), 4.23–4.14 (m, 3H, H-5″, H-6a’, H-6b’), 4.04 (t, *J* = 9.3 Hz, 1H, H-3′), 3.83 (ddd, *J* = 12.0, 8.8, 5.4 Hz, 3H, H-2, H-6b, H-4″), 3.67 (ddd, *J* = 10.0, 4.8, 2.8 Hz, 1H, H-5′), 2.74 (s, 4H), 2.17 (s, 3H, COC*H*_3_), 2.14 (s, 3H, COC*H*_3_), 2.11 (s, 3H, COC*H*_3_), 2.07 (s, 3H, COC*H*_3_), 2.01 (s, 3H, COC*H*_3_),1.88 (s, 3H COC*H*_3_), 1.81 (s, 3H, COC*H*_3_), 1.16 (d, *J* = 6.3 Hz, 3H, Rha-C*H*_3_). ^13^C NMR (151 MHz, CDCl_3_) δ 170.89 (2C), 170.52, 170.46, 170.05, 169.49, 169.44, 169.28, 169.12, 164.52, 133.24, 129.97, 129.08, 128.31 (Ar-C), 102.21, 101.18, 99.14, 79.95, 73.39, 72.87, 72.26, 70.86, 69.84, 69.78, 69.24, 69.07, 68.18, 67.36, 62.34, 61.87, 58.53, 25.41 (2C), 20.95 (2C), 20.83, 20.78, 20.64, 20.59, 20.45, 17.32. mass spectrum (HRMS), *m/z* = 1031.2860 (M+Na)^+^, C_43_H_52_N_4_O_24_ requires 1031.2864.

### Succinimidyl [2,3,4-tri-*O*-acetyl-α-L-rham-nopyranosyl]-(1→3)-[4,6-*O*-acetyl-2-*O*-benzoyl-β-D-glucopyranosyl]-(1→3)-2-*N*-tertbutyloxycarbonylalanine-4,6-*O*-acetyl-2-deoxy-α-D-galactopyranoside (21)

To a solution of trisaccharide **20** (0.10 g, 0.10 mmol) in dry DCM:AcOH (3:1, 8 ml), zinc dust (0.07 g, 1.01 mmol) was added, and the reaction was stirred under N_2_ at room temperature. After 1 day, the reaction was observed to be complete by TLC. The reaction was diluted with DCM (15 ml) and washed with aq. sodium bicarbonate (2 × 30 ml) and brine (2 × 30 ml) solution. The organic layer was separated, dried over anhydrous Na_2_SO_4_, and evaporated. The residue was dried and used for the next reaction without further purification. The residue was dissolved in dry DCM (3 ml), and Boc-Ala-OH (0.04 g, 0.20 mmol), T_3_P (0.06 ml, 0.20 mmol), and DIPEA (0.05 ml, 0.30 mmol) were successively added at 0°C. The solution was stirred under a N_2_ atmosphere and allowed to warm to room temperature. The reaction appeared complete after 12 h. The reaction was diluted with DCM (20 ml) and washed with 1N HCl (2 × 15 ml), followed by aq. sodium bicarbonate (2 × 20 ml) solution. The organic layer was collected and subjected to silica gel flash column chromatography to afford the compound as fluffy white solid **21**: yield 66% over two steps (75.0 mg); silica gel TLC *R*_*f*_ = 0.50 (100% ethyl acetate). ^1^H NMR (600 MHz, CDCl_3_) δ 8.13–8.02 (Ar-H, 2H), 7.55–7.39 (Ar-H, 3H), 6.74 (d, *J* = 9.3 Hz, 1H, N*H*), 5.58 (dd, *J* = 3.2, 1.3 Hz, 1H, H-4), 5.32–5.29 (m, 1H, C*H*), 5.27–5.14 (m, 3H, H-4′, H-2′, H-1), 5.10 (dd, *J* = 10.0, 2.8 Hz, 1H, H-2″), 4.92–4.84 (m, 4H, H-1′, H-3″, H-5, H-1″), 4.58 (ddd, *J* = 11.2, 9.2, 3.9 Hz, 1H, H-2), 4.33 (dd, *J* = 11.7, 4.7 Hz, 1H, H-6a), 4.20 (d, *J* = 4.3 Hz, 2H, H-6a’, H-6b’), 4.11–4.03 (m, 2H, H-3, H-3′), 4.00–3.91 (m, 1H, H-5″), 3.88–3.83 (m, 2H, H-6b, H-4″), 3.68–3.65 (m, 1H, H-5′), 2.72 (s, 4H), 2.17 (s, 3H, COC*H*_3_), 2.11 (s, 3H, COC*H*_3_), 2.10 (s, 3H, COC*H*_3_), 2.09 (s, 3H, COC*H*_3_), 2.00 (s, 3H, COC*H*_3_), 1.88 (s, 3H, COC*H*_3_), 1.79 (s, 3H, COC*H*_3_), 1.44 (s, 12H, Ala-C*H*_3_, ^t^Bu-H), 1.15 (d, *J* = 6.3 Hz, 3H, Rha-C*H*_3_). ^13^C NMR (151 MHz, CDCl_3_) δ 172.33, 170.96 (2C), 170.69, 170.48, 170.08, 169.94, 169.33, 169.23, 169.04, 164.39, 133.03, 130.15, 129.31, 128.19, 104.34, 100.20, 99.09, 79.92, 79.83, 72.93, 72.04, 71.66, 70.92, 69.78, 69.76, 69.08, 68.75, 68.22, 67.28, 62.42, 61.65, 53.46, 48.31, 28.38 (3C), 25.35 (2C), 20.94 (2C), 20.87, 20.79, 20.60, 20.58, 20.42, 17.33 (2C). mass spectrum (HRMS), *m/z* = 1176.3854 (M+Na)^+^, C_51_H_67_N_3_O_27_ requires 1176.3854.

### Aminooxy [α-L-rhamnopyranosyl]-(1→3)-[β-D-glucopyranosyl]-(1→3)-2-*N*-alanine-2-deoxy-α-D-galactopyranoside (4)

Compound **21** (60.0 mg, 0.05 mmol) was dissolved in trifluoroacetic acid (TFA)/DCM (3 ml, 1:1) and stirred for 1 h. The mixture was diluted with DCM (10 ml) and washed with sodium bicarbonate solution (2 × 20 ml); the organic layer was separated, dried over Na_2_SO_4_, and filtered. The filtrate was evaporated, dried, and used for the next reaction without further purification. The residue (40.0 mg, 0.03 mmol) was dissolved in methanol (3 ml), and then hydrazine hydrate (0.03 ml, 0.62 mmol) was added and the reaction was stirred for 10 h. The reaction mixture was then concentrated to dryness. This residue was dissolved in a minimal amount of water and purified using P-2 biogel with water as the eluent (collecting ∼0.2 ml fractions), to provide as a white solid **4**: yield 35% over two steps (10 mg); silica gel TLC *R*_*f*_ = 0.20 (50% methanol: DCM). ^1^H NMR (600 MHz, D_2_O) δ 5.00 (brs, 1H, H-1″), 4.86 (d, *J* = 4.0 Hz, 1H, H-1), 4.43 (d, *J* = 8.0 Hz, 1H, H-1′), 4.29 (dd, *J* = 11.3, 4.1 Hz, 1H, H-2), 4.17 (dd, *J* = 3.1, 1.2 Hz, 1H, H-4), 3.99–3.82 (m, 4H, H-5, H-3, H-2″, C*H*), 3.76 (dd, *J* = 12.4, 2.3 Hz, 1H, H-6a), 3.72–3.59 (m, 5H, H-3″, H-5″, H-6b, H-6a’, H-6b’), 3.44 (t, *J* = 9.1 Hz, 1H, H-3′), 3.40–3.23 (m, 4H, H-2′, H-4′, H-4″, H-5′), 1.41 (d, *J* = 7.1 Hz, 3H, Ala-C*H*_3_), 1.13 (d, *J* = 6.3 Hz, 3H, Rha-C*H*_3_). ^13^C NMR (151 MHz, D_2_O) δ 171.64, 103.50, 100.91, 100.44, 82.06, 76.69, 75.61, 73.53, 71.86, 70.60, 70.25, 70.11, 68.75, 68.41, 67.64, 61.01, 60.30, 49.32, 48.03, 16.77, 16.38. mass spectrum (HRMS), *m/z* = 574.2444 (M + H)^+^, C_21_H_39_N_3_O_15_ requires 574.2453.

## Data Availability

The original contributions presented in the study are included in the Supplementary Material; further inquiries can be directed to the corresponding author.
