# Supplementary material for: Synthesis of Aminooxy Glycoside Derivatives of the Outer Core Domain of Pseudomonas aeruginosa Lipopolysaccharide
Source: Front Mol Biosci. 2021 Nov 8;8:750502. doi: 10.3389/fmolb.2021.750502 (PMC8606414; doi:10.3389/fmolb.2021.750502)

Supporting Information
for

Synthesis of aminooxy glycoside derivatives of the outer core domain of *Pseudomonas aeruginosa* lipopolysaccharide

Anshupriya Si, Steven J Sucheck

Department of Chemistry & Biochemistry, University of Toledo, Toledo, OH 43606, United States.

Table of Contents

Title of paper, author's names, address	S1
HRMS data of Succinimidyl 2-azido-3-*O*-acetate-4,6-*O*-benzylidene-2-deoxy-α-D-galactopyranoside (**8**)	S5
^{1}H and ^{13}C NMR of Succinimidyl 2-azido-3-*O*-acetate-4,6-*O*-benzylidene-2-deoxy-α-D-galactopyranoside (**8**)	S6
HRMS data of Succinimidyl 2-azido-3-*O*-acetate-6-*O*-benzyl-2-deoxy-α-D-galactopyranoside (**9**)	S7
^{1}H and ^{13}C NMR of Succinimidyl 2-azido-3-*O*-acetate-6-*O*-benzyl-2-deoxy-α-D-galactopyranoside (**9**)	S8
HRMS data of Succinimidyl [2,3,4-tri-*O*-benzyl-6-tertbutyldiphenylsillyl-α-D-glucopyranosyl]-(1→4)-2-azido-3-*O*-acetate-6-*O*-benzyl-2-deoxy-α-D-galactopyranoside (**10**)	S9
^{1}H and ^{13}C NMR of Succinimidyl [2,3,4-tri-*O*-benzyl-6-tertbutyldiphenylsillyl-α-D-glucopyranosyl]-(1→4)-2-azido-3-*O*-acetate-6-*O*-benzyl-2-deoxy-α-D-galactopyranoside (**10**)	S10
HRMS data of Succinimidyl [2,3,4-tri-*O*-benzyl-α-D-glucopyranosyl]-(1→4)-2-azido-3-*O*-acetate-6-*O*-benzyl-2-deoxy α-D-galactopyranoside (**11**)	S11
^{1}H and ^{13}C NMR of Succinimidyl [2,3,4-tri-*O*-benzyl-α-D-glucopyranosyl]-(1→4)-2-azido-3-*O*-acetate-6-*O*-benzyl-2-deoxy α-D-galactopyranoside (**11**)	S12
HRMS data of Succinimidyl [2-*O*-acetyl-3,4-di-*O*-benzyl-α-L-rhamnopyranosyl]-(1→6)-[2,3,4-tri-*O*-benzyl-α-D-glucopyranosyl]-(1→4)-2-azido-3-*O*-acetate-6-*O*-benzyl-2-deoxy-α-D-galactopyranoside (**12**)	S13
^{1}H and ^{13}C NMR of Succinimidyl [2-*O*-acetyl-3,4-di-*O*-benzyl-α-L-rhamnopyranosyl]-(1→6)-[2,3,4-tri-*O*-benzyl-α-D-glucopyranosyl]-(1→4)-2-azido-3-*O*-acetate-6-*O*-benzyl-2-deoxy-α-D-galactopyranoside (**12**)	S14
HRMS data of Succinimidyl [2-*O*-acetyl-3,4-di-*O*-benzyl-α-L-rhamnopyranosyl]-(1→6)-[2,3,4-tri-*O*-benzyl-α-D-glucopyranosyl]-(1→4)-2-*N*-benzyloxycarbonylalanine-3-*O*-acetate-6-*O*-benzyl-2-deoxy-α-D-galactopyranoside (**13**)	S15
^{1}H and ^{13}C NMR of Succinimidyl [2-*O*-acetyl-3,4-di-*O*-benzyl-α-L-rhamnopyranosyl]-(1→6)-[2,3,4-tri-*O*-benzyl-α-D-glucopyranosyl]-(1→4)-2-*N*-benzyloxycarbonylalanine-3-*O*-acetate-6-*O*-benzyl-2-deoxy-α-D-galactopyranoside (**13**)	S16

HRMS data of Aminooxy [α-L-rhamnopyranosyl]-(1→6)-[α-D-glucopyranosyl]-(1→4)-2-*N*-alanine-2-deoxy-α-D-galactopyranoside (**3**) S17

^{1}H and ^{13}C NMR of Aminooxy [α-L-rhamnopyranosyl]-(1→6)-[α-D-glucopyranosyl]-(1→4)-2-*N*-alanine-2-deoxy-α-D-galactopyranoside (**3**) S18

HRMS data of Succinimidyl 2-azido-4,6-*O*-benzylidene-3-*O*-chloroacetyl-2-deoxy-α-D-galactopyranoside (**15**) S19

^{1}H and ^{13}C NMR of Succinimidyl 2-azido-4,6-*O*-benzylidene-3-*O*-chloroacetyl-2-deoxy-α-D-galactopyranoside (**15**) S20

^{1}H and ^{13}C NMR of Succinimidyl 2-azido-4,6-*O*-benzylidene-2-deoxy-α-D-galactopyranoside (**16**) S21

HRMS data of Succinimidyl [2-*O*-benzoyl-4,6-*O*-benzylidene-3-*O*-(p-methoxy)benzyl-β-D-glucopyranosyl]-(1→3)-2-azido-4,6-*O*-benzylidene-2-deoxy-α-D-galactopyranoside (**17**) S22

^{1}H and ^{13}C NMR of Succinimidyl [2-*O*-benzoyl-4,6-*O*-benzylidene-3-*O*-(p-methoxy)benzyl-β-D-glucopyranosyl]-(1→3)-2-azido-4,6-*O*-benzylidene-2-deoxy-α-D-galactopyranoside (**17**) S23

HRMS data of Succinimidyl [2-*O*-benzoyl-4,6-*O*-benzylidene-β-D-glucopyranosyl]-(1→3)-2-azido-4,6-*O*-benzylidene-2-deoxy-α-D-galactopyranoside (**18**) S24

^{1}H and ^{13}C NMR of Succinimidyl [2-*O*-benzoyl-4,6-*O*-benzylidene-β-D-glucopyranosyl]-(1→3)-2-azido-4,6-*O*-benzylidene-2-deoxy-α-D-galactopyranoside (**18**) S25

HRMS data of Succinimidyl [2,3,4-tri-*O*-acetyl-α-L-rhamnopyranosyl]-(1→3)-[2-*O*-benzoyl-4,6-*O*-benzylidene-β-D-glucopyranosyl]-(1→3)-2-azido-4,6-*O*-benzylidene-2-deoxy-α-D-galactopyranoside (**19**) S26

^{1}H and ^{13}C NMR of Succinimidyl [2,3,4-tri-*O*-acetyl-α-L-rhamnopyranosyl]-(1→3)-[2-*O*-benzoyl-4,6-*O*-benzylidene-β-D-glucopyranosyl]-(1→3)-2-azido-4,6-*O*-benzylidene-2-deoxy-α-D-galactopyranoside (**19**) S27

HRMS data of Succinimidyl [2,3,4-tri-*O*-acetyl-α-L-rhamnopyranosyl]-(1→3)-[4,6-*O*-acetyl-2-*O*-benzoyl-β-D-glucopyranosyl]-(1→3)-2-azido-4,6-*O*-acetyl-2-deoxy-α-D-galactopyranoside (**20**) S28

^{1}H and ^{13}C NMR of Succinimidyl [2,3,4-tri-*O*-acetyl-α-L-rhamnopyranosyl]-(1→3)-[4,6-*O*-acetyl-2-*O*-benzoyl-β-D-glucopyranosyl]-(1→3)-2-azido-4,6-*O*-acetyl-2-deoxy-α-D-galactopyranoside (**20**) S29

HRMS data of Succinimidyl [2,3,4-tri-*O*-acetyl-α-L-rhamnopyranosyl]-(1→3)-[4,6-*O*-acetyl-2-*O*-benzoyl-β-D-glucopyranosyl]-(1→3)-2-*N*-tertbutyloxycarbonylalanine-4,6-*O*-acetyl-2-deoxy-α-D-galactopyranoside (**21**) S30

^{1}H and ^{13}C NMR of Succinimidyl [2,3,4-tri-*O*-acetyl-α-L-rhamnopyranosyl]-(1→3)-[4,6-*O*-acetyl-2-*O*-benzoyl-β-D-glucopyranosyl]-(1→3)-2-*N*-tertbutyloxycarbonylalanine-4,6-*O*-acetyl-2-deoxy-α-D-galactopyranoside (**21**) S31

HRMS data of Aminooxy [α-L-rhamnopyranosyl]-(1→3)-[β-D-glucopyranosyl]-(1→3)-2-*N*-alanine-2-deoxy-α-D-galactopyranoside (**4**) S32

^{1}H and ^{13}C NMR of Aminooxy [α-L-rhamnopyranosyl]-(1→3)-[β-D-glucopyranosyl]-(1→3)-2-*N*-alanine-2-deoxy-α-D-galactopyranoside (**4**) S33

HRMS data of Succinimidyl 2-azido-3-*O*-acetate-4,6-*O*-benzylidene-2-deoxy-α-D-galactopyranoside (**8**)

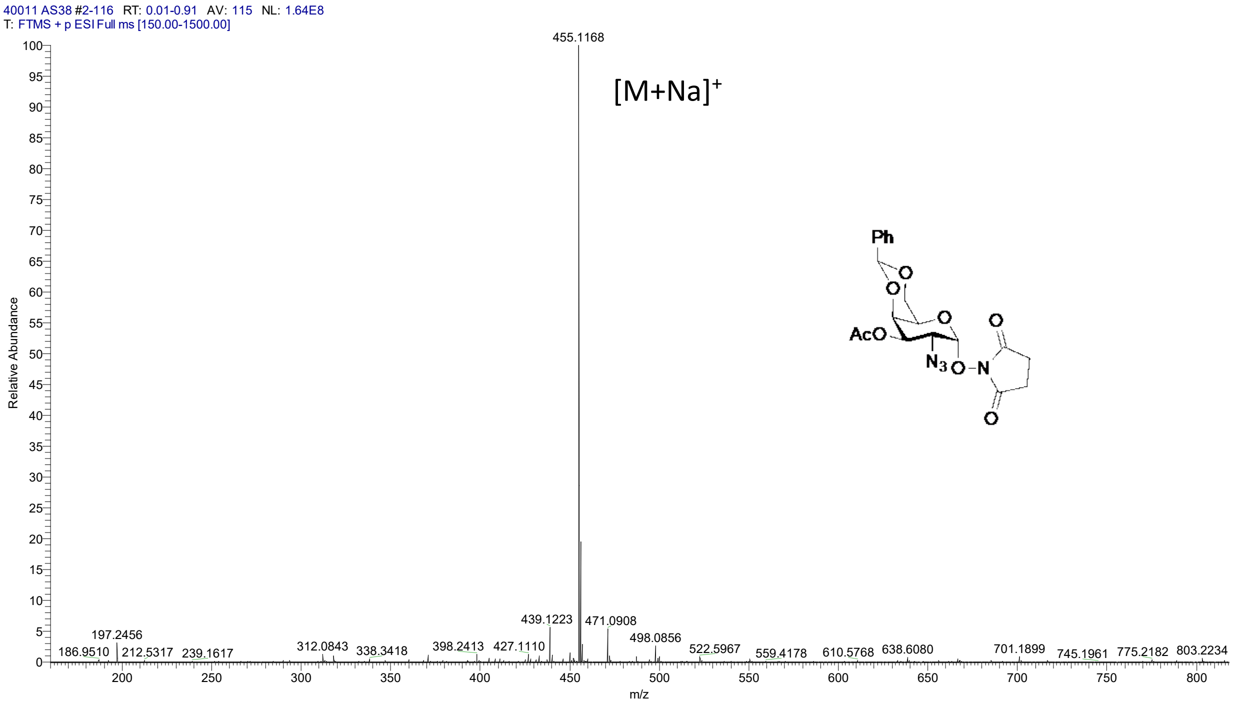

Mass accuracy = (455.1168 – 455.1173)/455.1173)* 10^6 = 1.1 ppm

^{1}H and ^{13}C NMR of Succinimidyl 2-azido-3-*O*-acetate-4,6-*O*-benzylidene-2-deoxy-α-D-galactopyranoside (**8**)

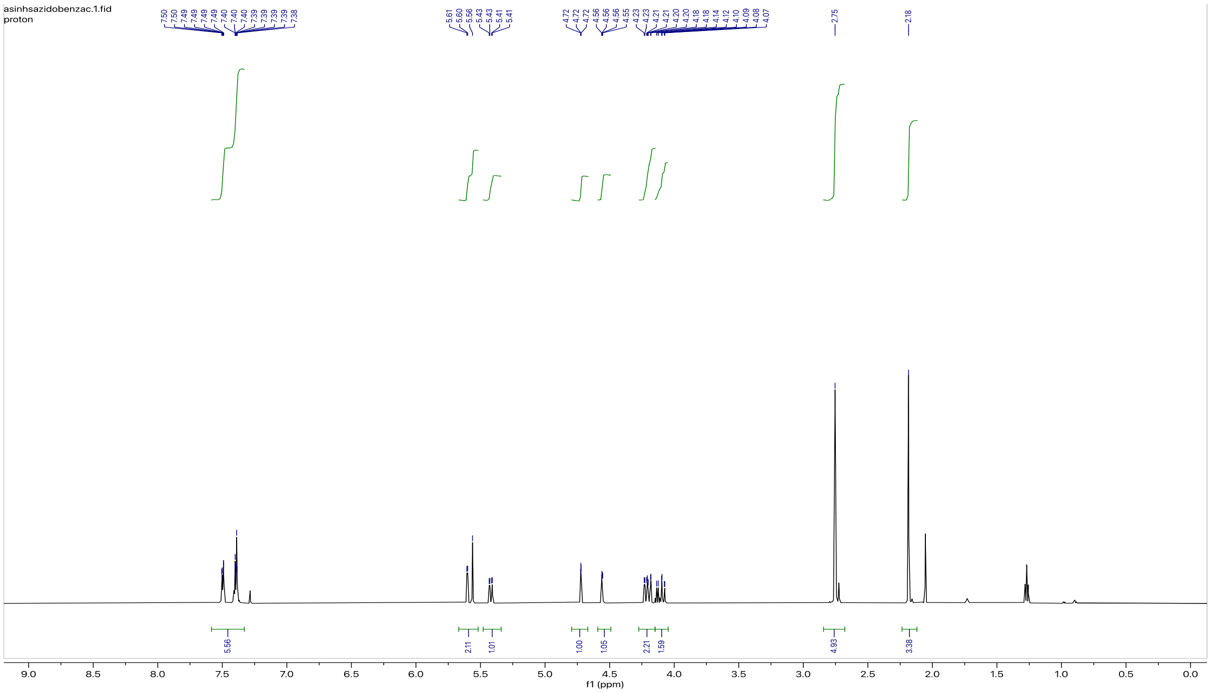

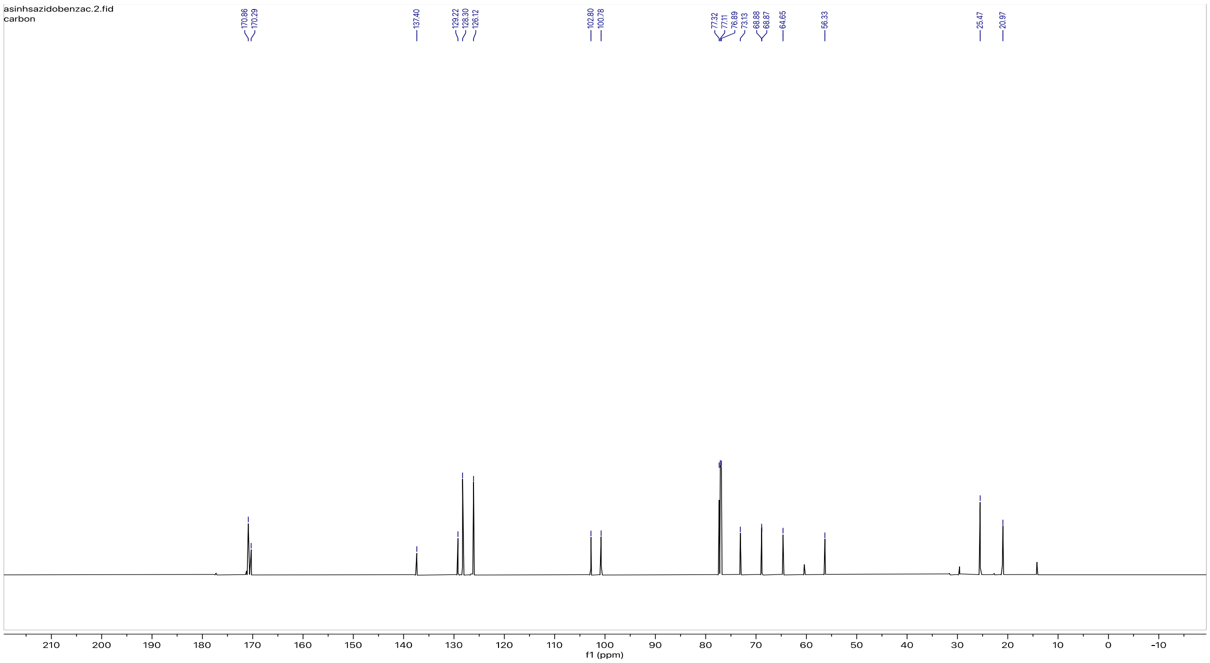

HRMS data of Succinimidyl 2-azido-3-*O*-acetate-6-*O*-benzyl-2-deoxy-α-D-galactopyranoside (**9**)

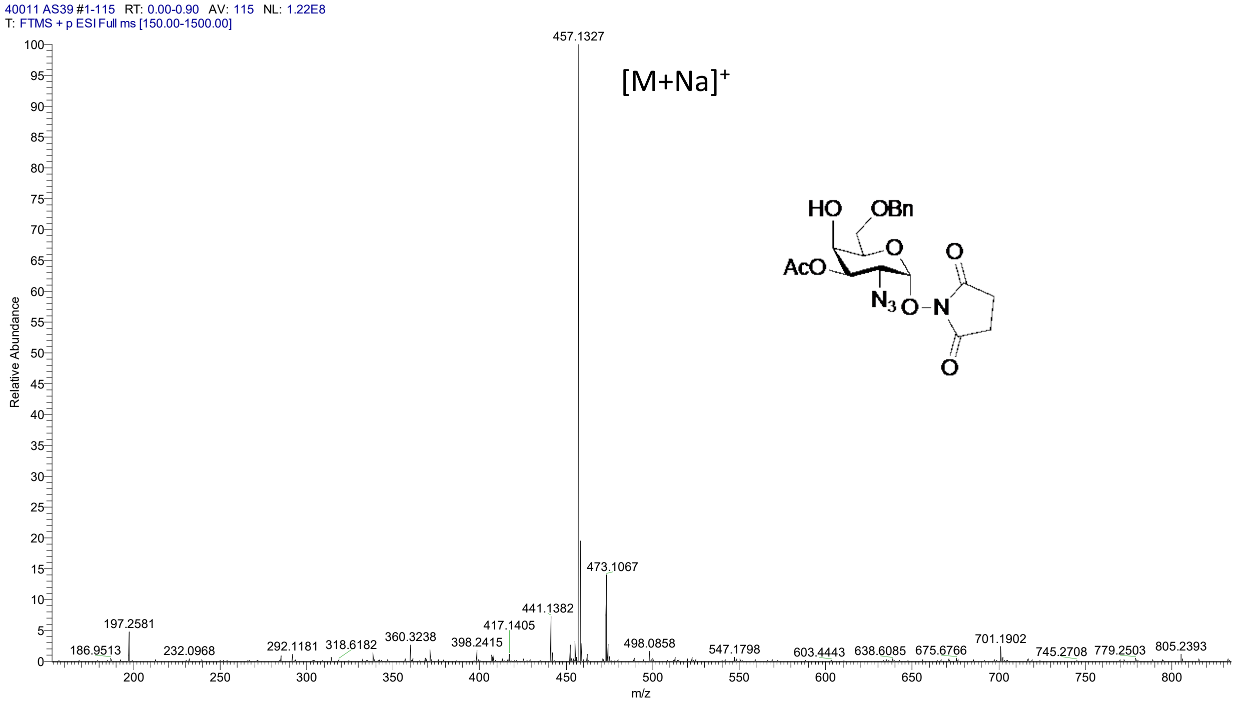

Mass accuracy = (457.1327 – 457.1330)/457.1330)* 10^6 = 0.7 ppm

^{1}H and ^{13}C NMR of Succinimidyl 2-azido-3-*O*-acetate-6-*O*-benzyl-2-deoxy-α-D-galactopyranoside (**9**)

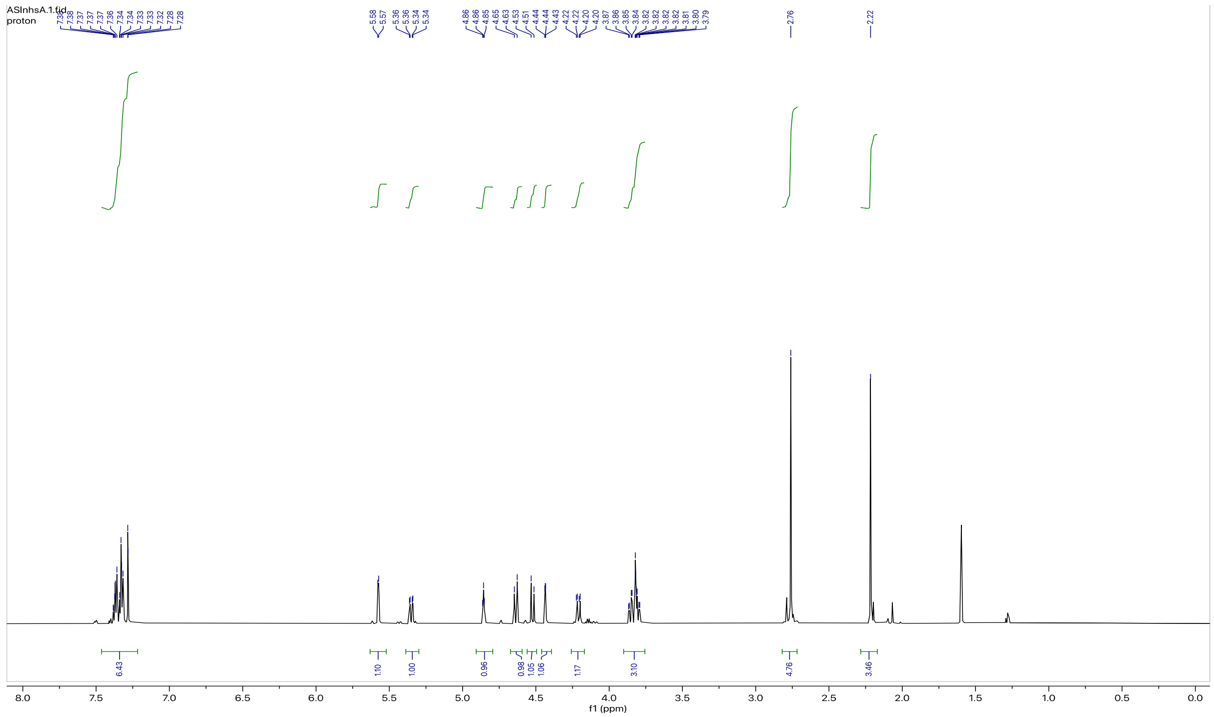

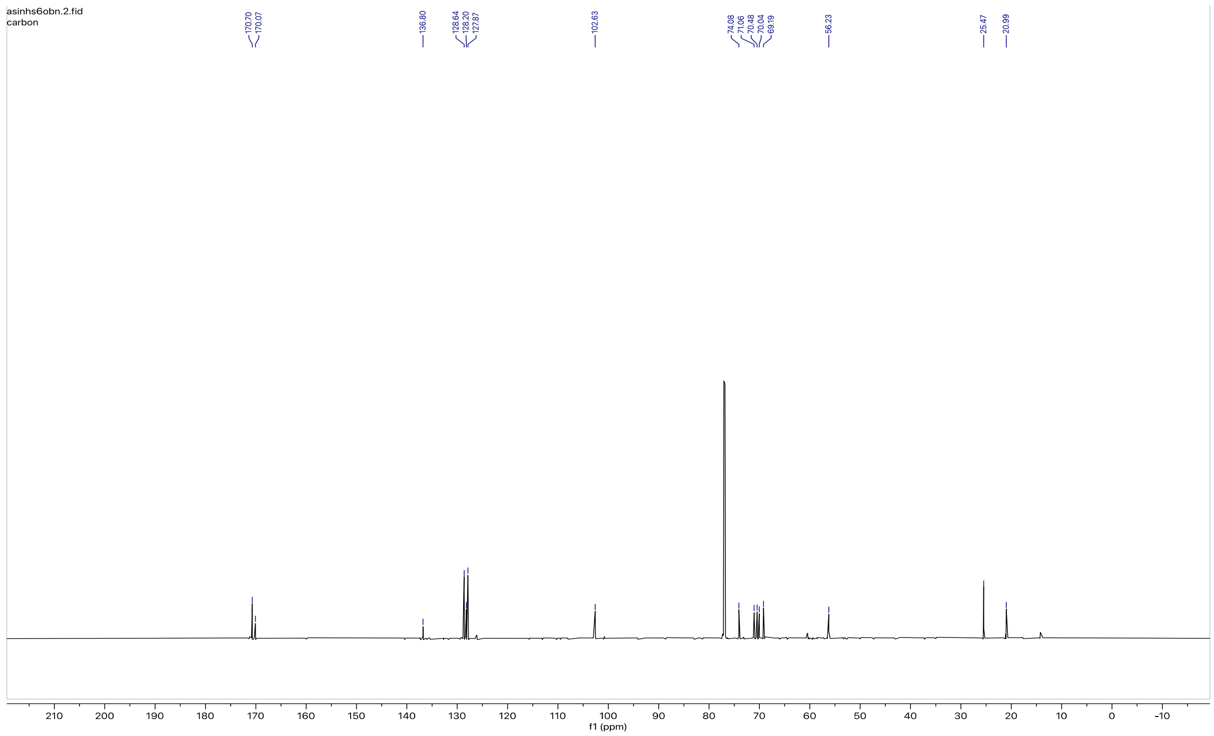

HRMS data of Succinimidyl [2,3,4-tri-*O*-benzyl-6-tertbutyldiphenylsillyl-α-D-glucopyranosyl]-(1→4)-2-azido-3-*O*-acetate-6-*O*-benzyl-2-deoxy-α-D-galactopyranoside (**10**)

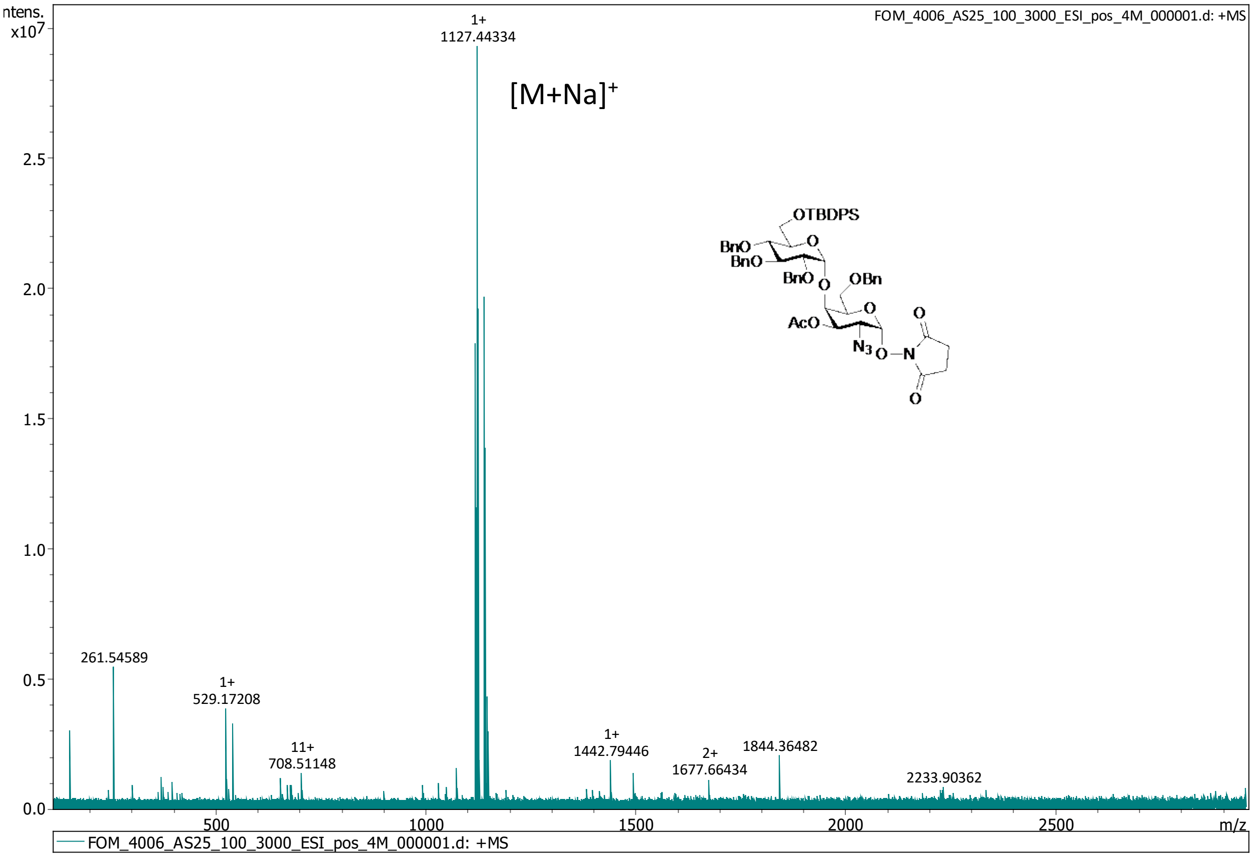

Mass accuracy = (1127.4434 – 1127.4552)/1127.4552)* 10^6 = 0.9 ppm

^{1}H and ^{13}C NMR of Succinimidyl [2,3,4-tri-*O*-benzyl-6-tertbutyldiphenylsillyl-α-D-glucopyranosyl]-(1→4)-2-azido-3-*O*-acetate-6-*O*-benzyl-2-deoxy-α-D-galactopyranoside (**10**)

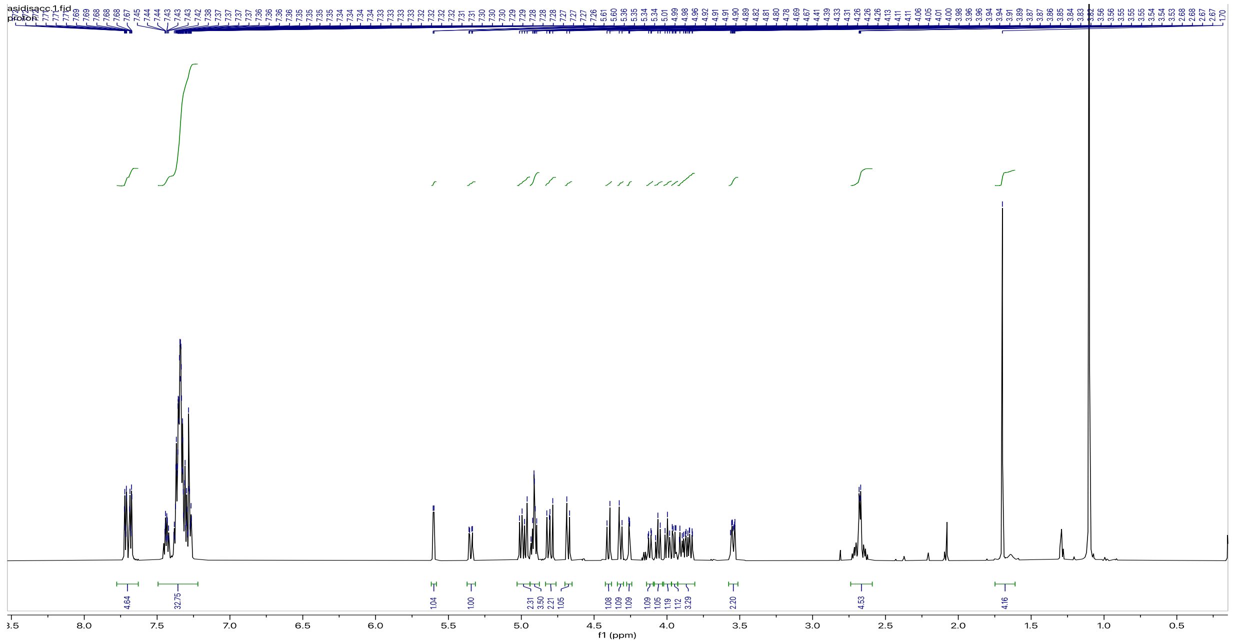

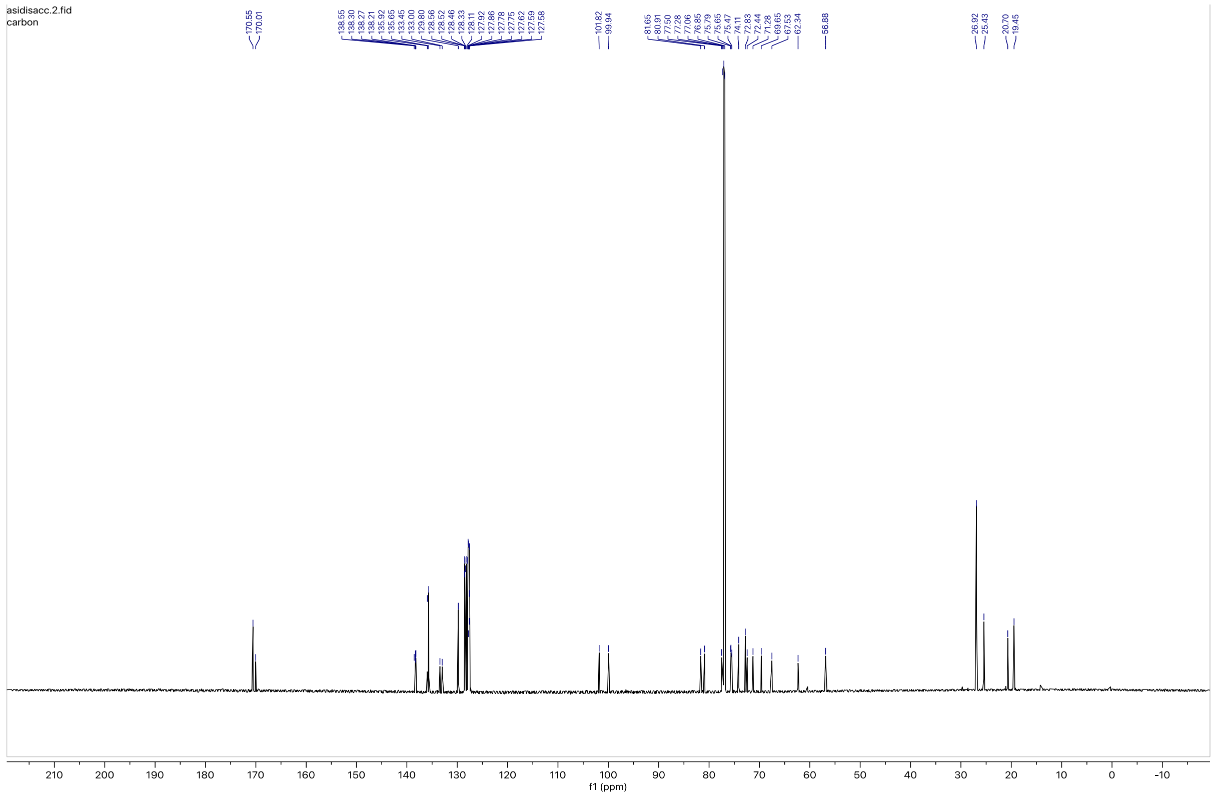

HRMS data of Succinimidyl [2,3,4-tri-*O*-benzyl-α-D-glucopyranosyl]-(1→4)-2-azido-3-*O*-acetate-6-*O*-benzyl-2-deoxy α-D-galactopyranoside (**11**)

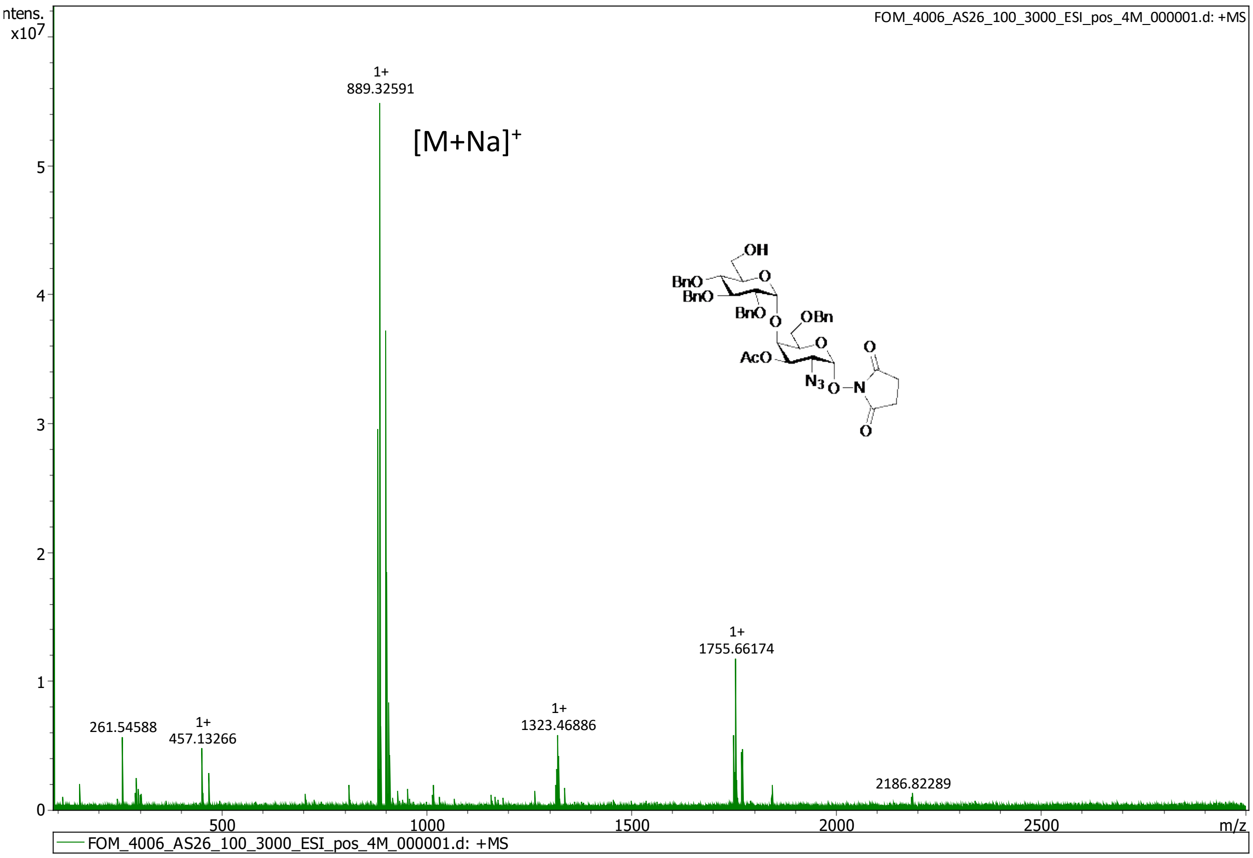

Mass accuracy = (889.3259 – 889.3374)/889.3374)* 10^6 = 0.8 ppm

^{1}H and ^{13}C NMR of Succinimidyl [2,3,4-tri-*O*-benzyl-α-D-glucopyranosyl]-(1→4)-2-azido-3-*O*-acetate-6-*O*-benzyl-2-deoxy α-D-galactopyranoside (**11**)

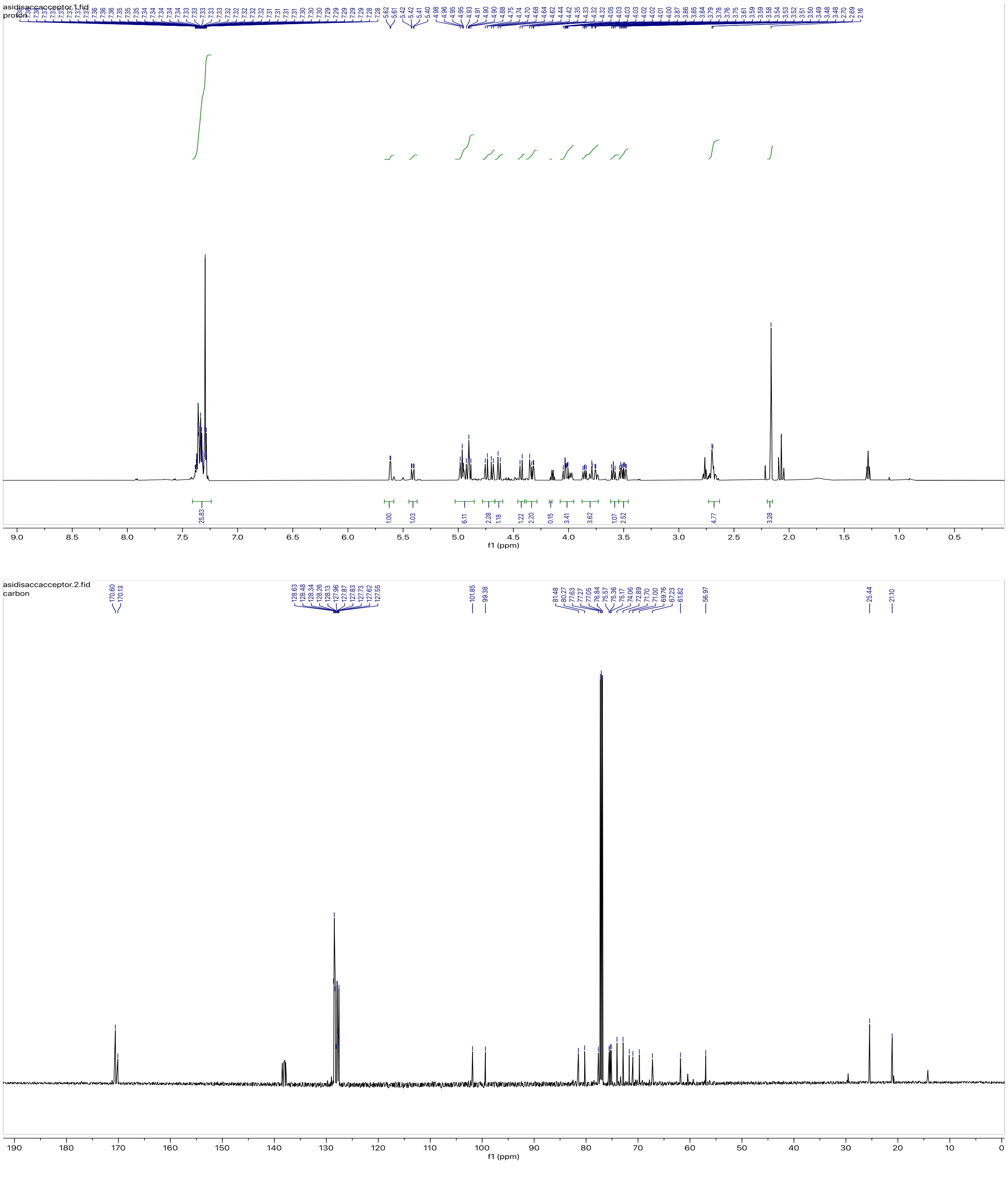

HRMS data of Succinimidyl [2-*O*-acetyl-3,4-di-*O*-benzyl-α-L-rhamnopyranosyl]-(1→6)-[2,3,4-tri-*O*-benzyl-α-D-glucopyranosyl]-(1→4)-2-azido-3-*O*-acetate-6-*O*-benzyl-2-deoxy-α-D-galactopyranoside (**12**)

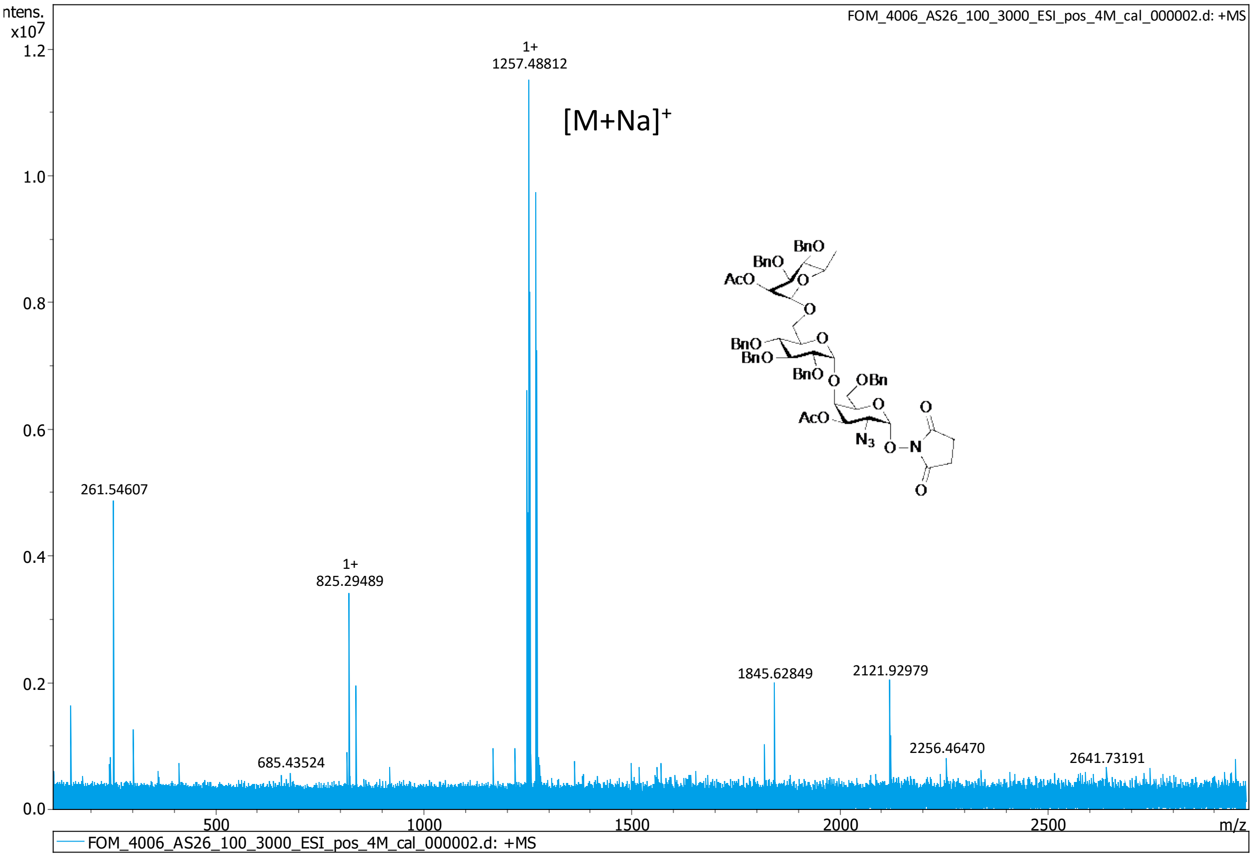

Mass accuracy = (1257.4881 – 1257.4998)/1257.4998)* 10^6 = 0.7 ppm

^{1}H and ^{13}C NMR of Succinimidyl [2-*O*-acetyl-3,4-di-*O*-benzyl-α-L-rhamnopyranosyl]-(1→6)-[2,3,4-tri-*O*-benzyl-α-D-glucopyranosyl]-(1→4)-2-azido-3-*O*-acetate-6-*O*-benzyl-2-deoxy-α-D-galactopyranoside (**12**)

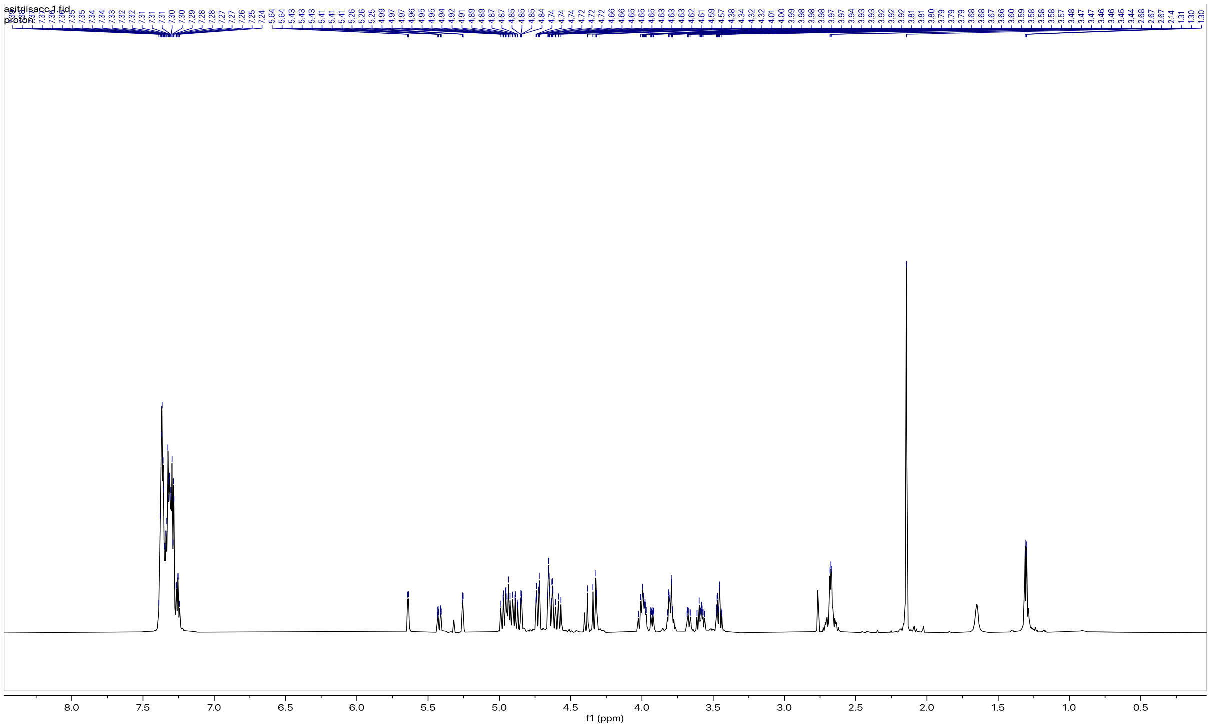

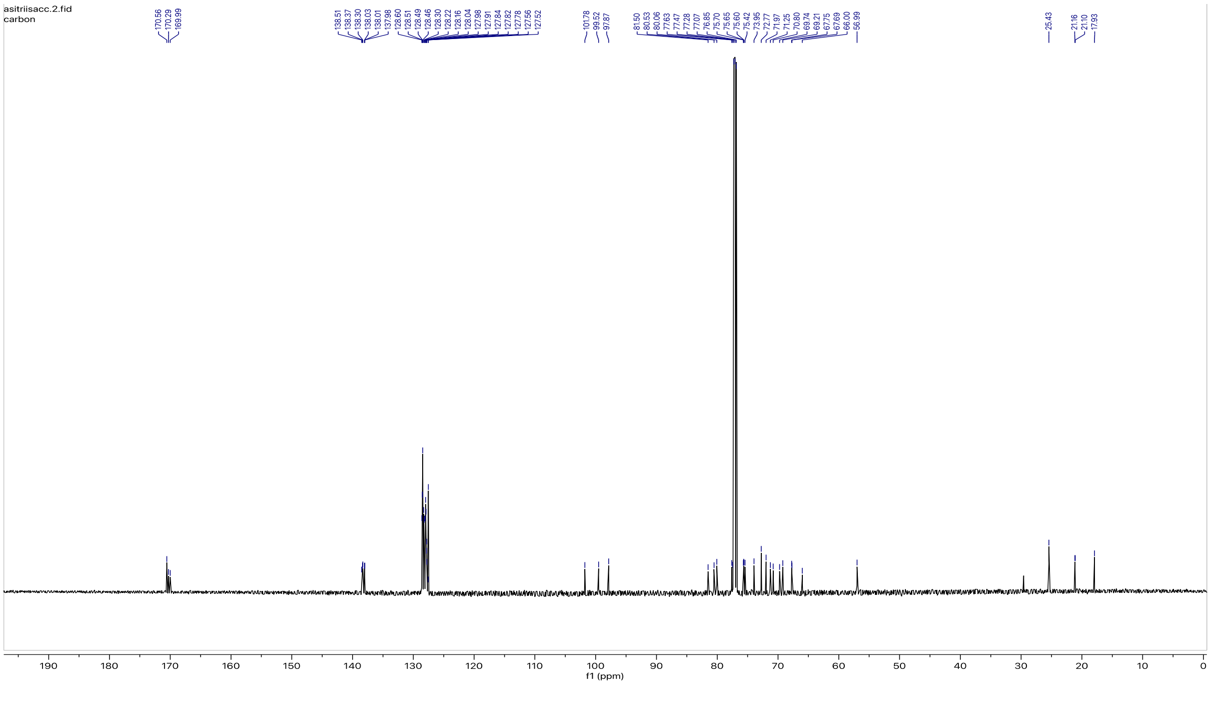

HRMS data of Succinimidyl [2-*O*-acetyl-3,4-di-*O*-benzyl-α-L-rhamnopyranosyl]-(1→6)-[2,3,4-tri-*O*-benzyl-α-D-glucopyranosyl]-(1→4)-2-*N*-benzyloxycarbonylalanine-3-*O*-acetate-6-*O*-benzyl-2-deoxy-α-D-galactopyranoside (**13**)

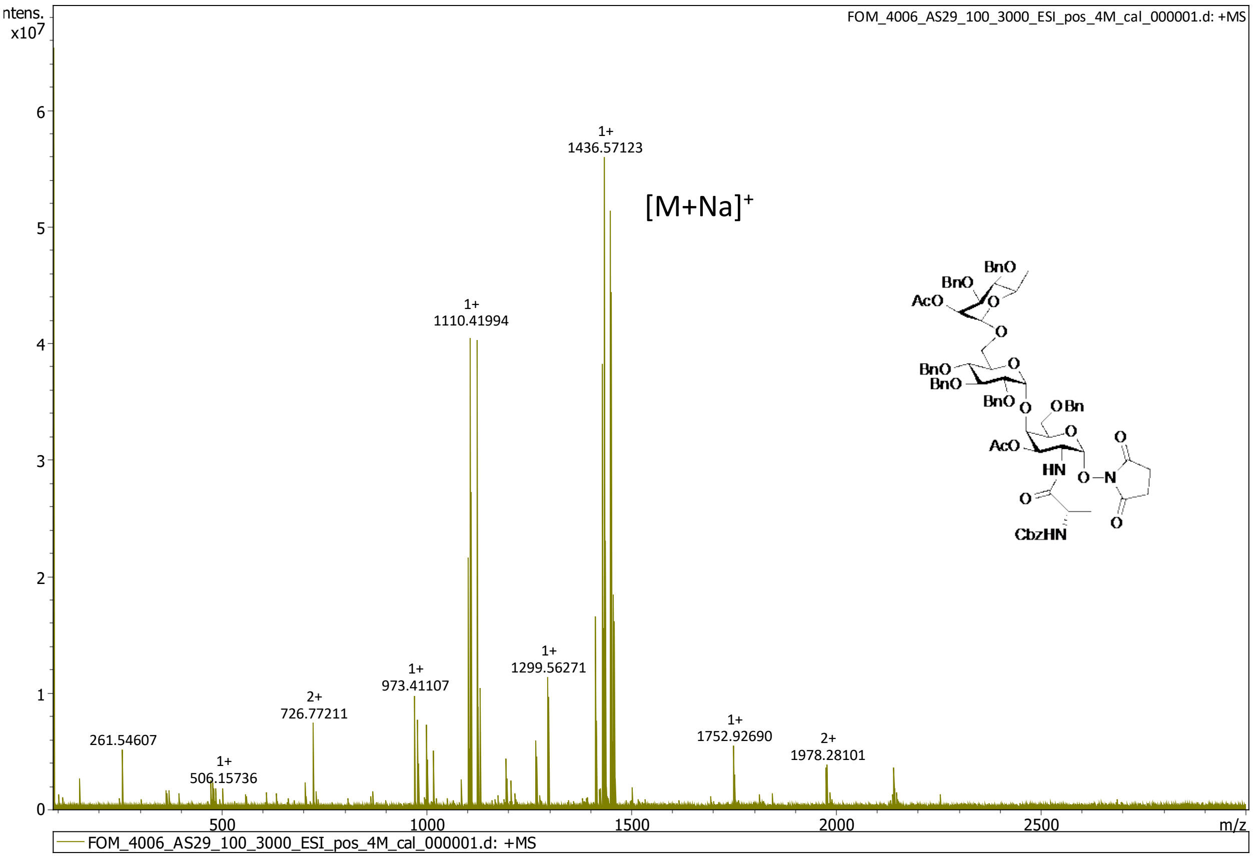

Mass accuracy = (1436.5712 – 1436.5832)/1436.5832)* 10^6 = 8.4 ppm

^{1}H and ^{13}C NMR of Succinimidyl [2-*O*-acetyl-3,4-di-*O*-benzyl-α-L-rhamnopyranosyl]-(1→6)-[2,3,4-tri-*O*-benzyl-α-D-glucopyranosyl]-(1→4)-2-*N*-benzyloxycarbonylalanine-3-*O*-acetate-6-*O*-benzyl-2-deoxy-α-D-galactopyranoside (**13**)

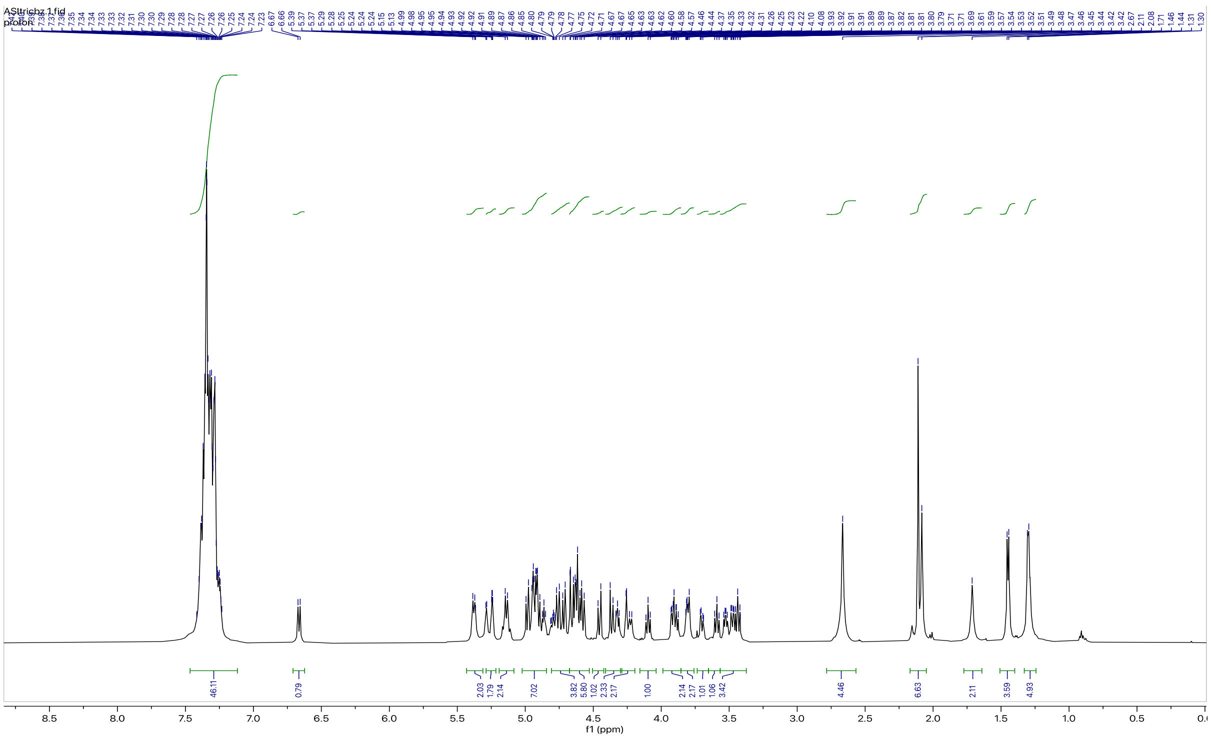

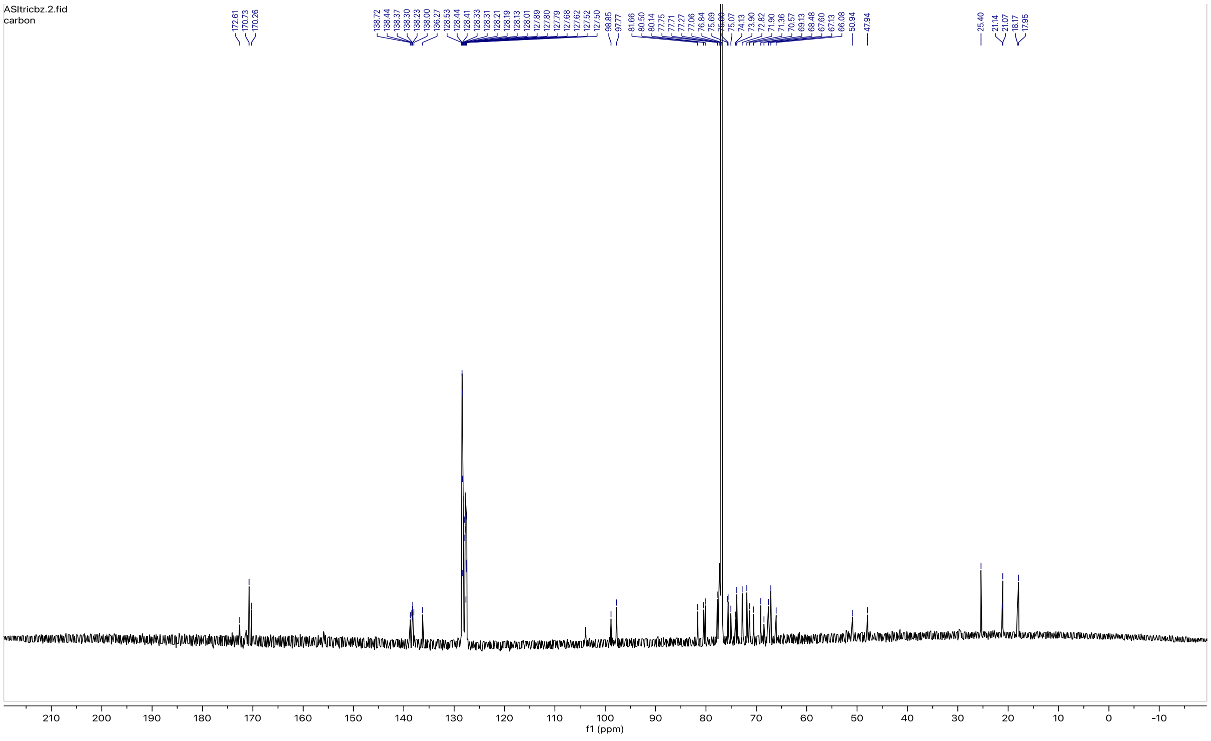

HRMS data of Aminooxy [α-L-rhamnopyranosyl]-(1→6)-[α-D-glucopyranosyl]-(1→4)-2-*N*-alanine-2-deoxy-α-D-galactopyranoside (**3**)

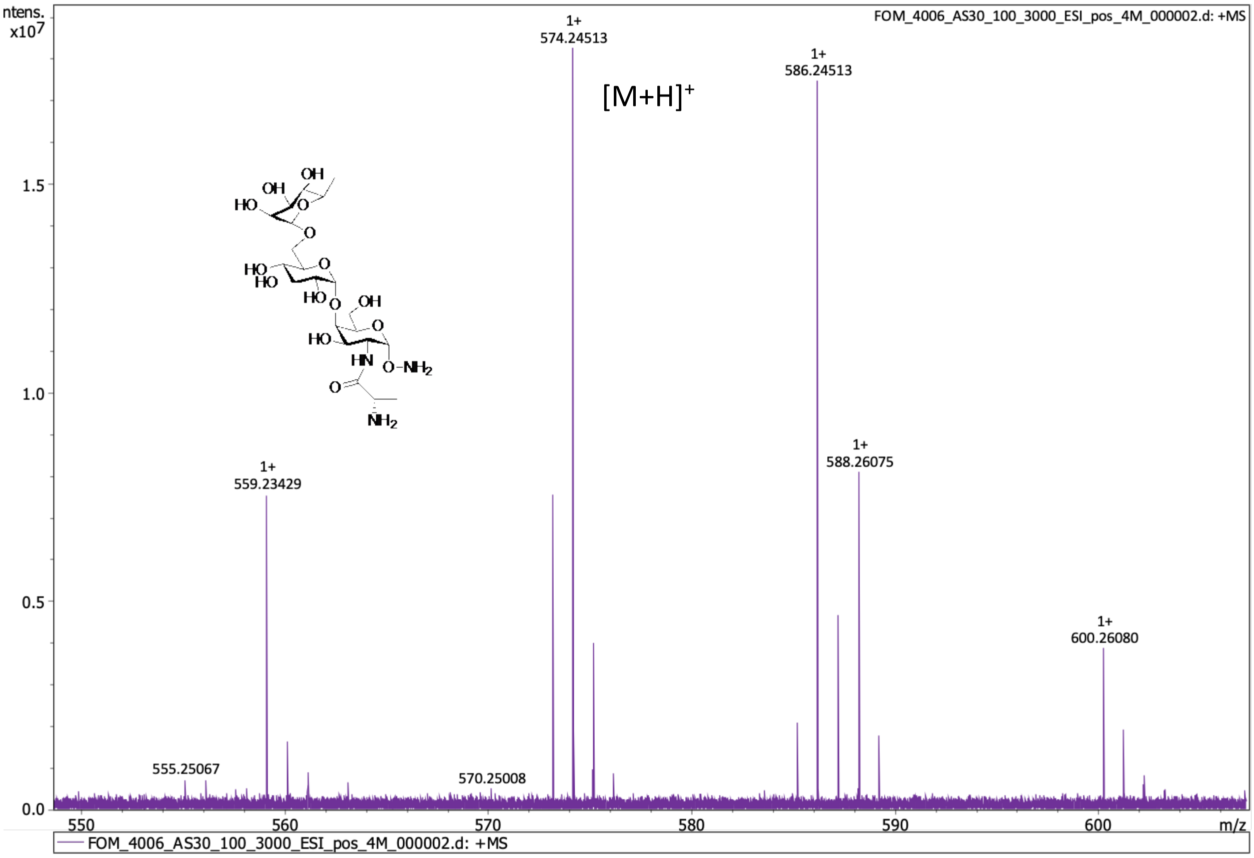

Mass accuracy = (574.2451 – 574.2381)/574.2381)* 10^6 = 0.5 ppm

^{1}H and ^{13}C NMR of Aminooxy [α-L-rhamnopyranosyl]-(1→6)-[α-D-glucopyranosyl]-(1→4)-2-*N*-alanine-2-deoxy-α-D-galactopyranoside (**3**)

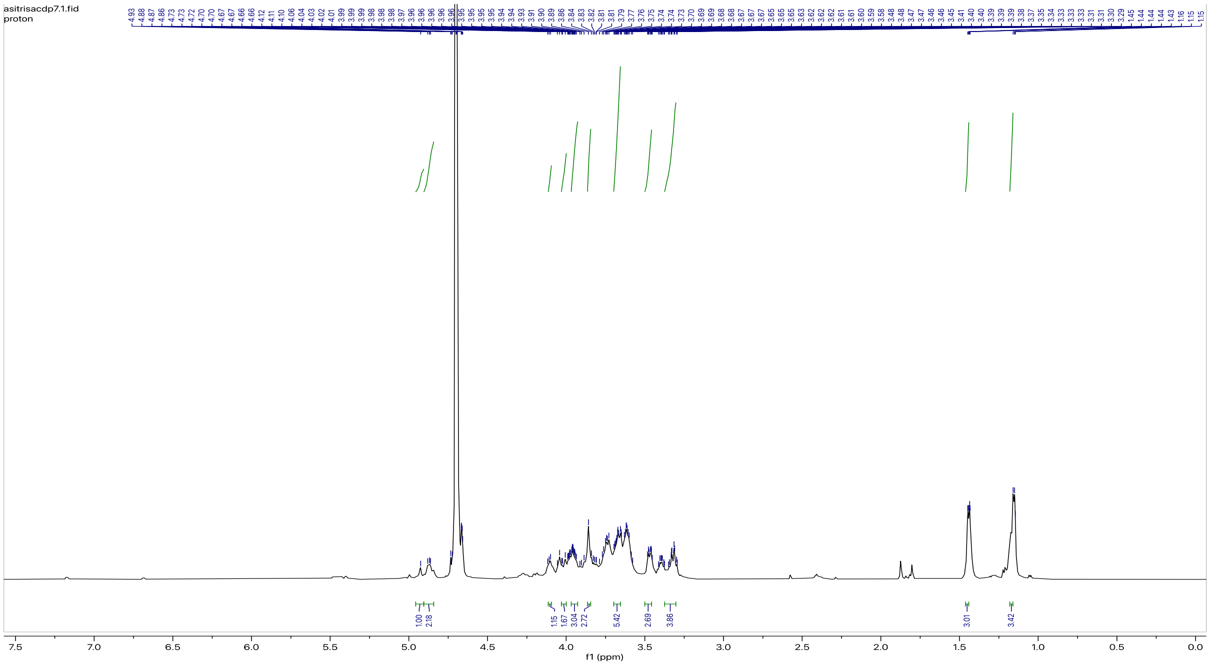

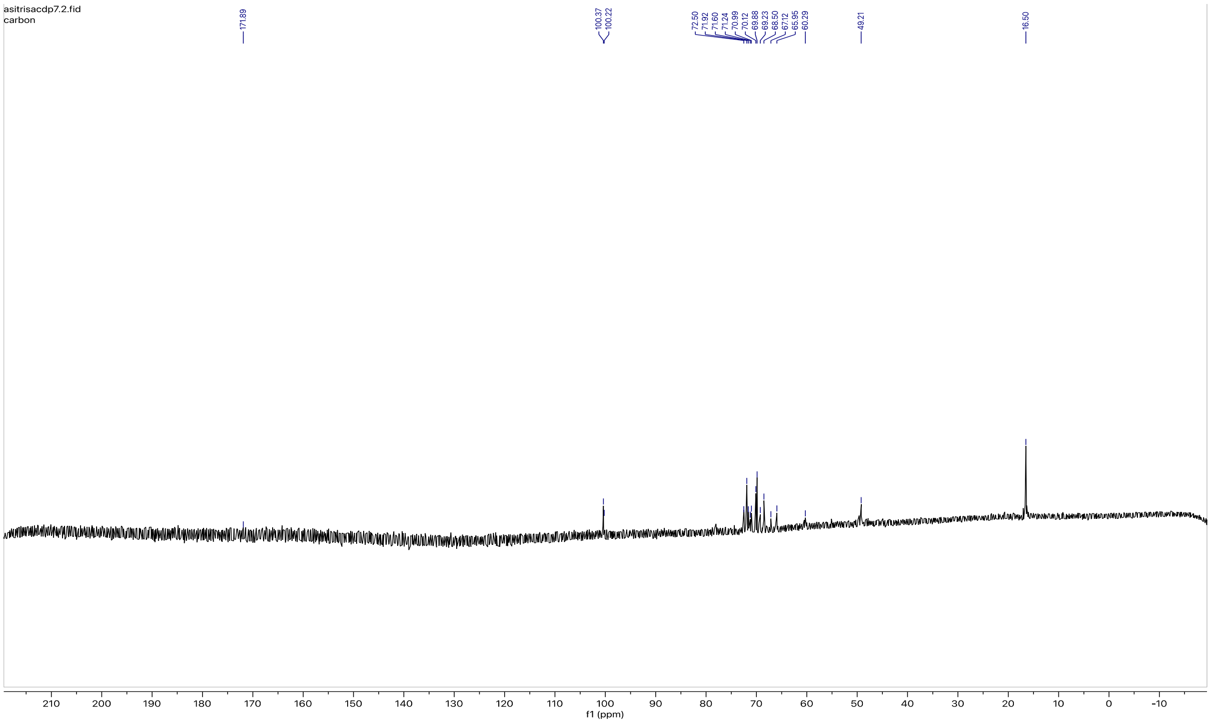

HRMS data of Succinimidyl 2-azido-4,6-*O*-benzylidene-3-*O*-chloroacetyl-2-deoxy-α-D-galactopyranoside (**15**)

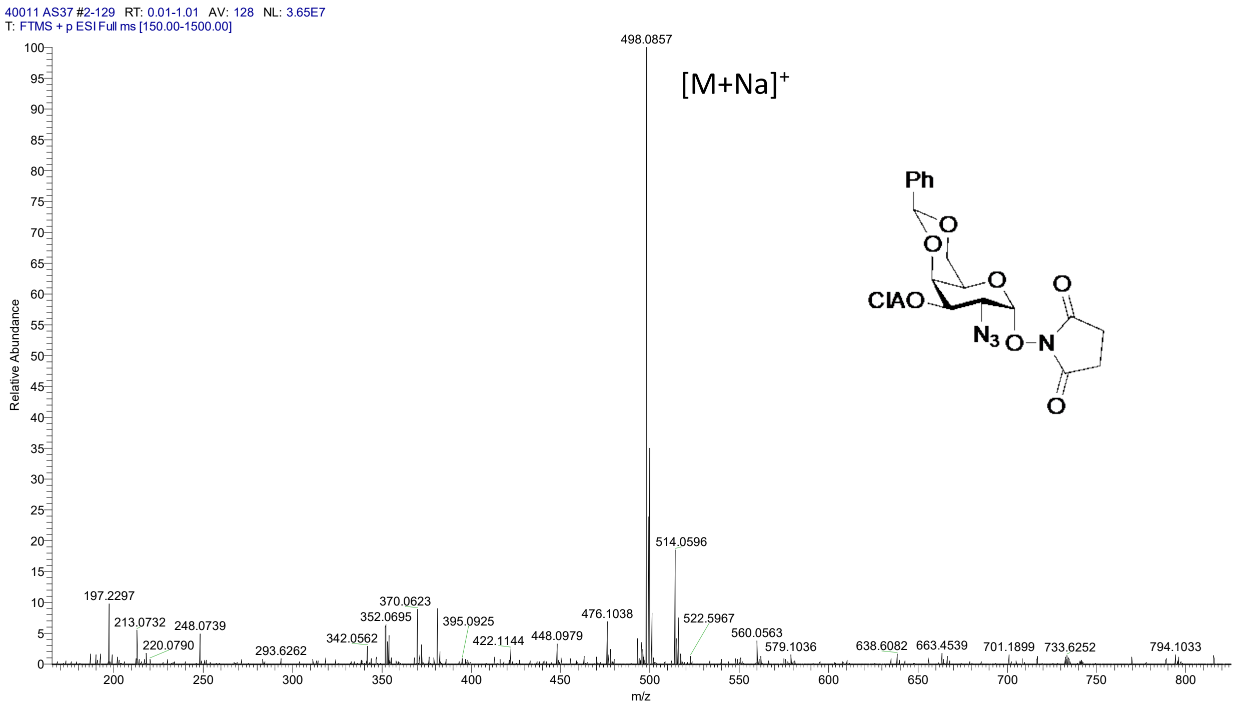

Mass accuracy = (498.0857 – 498.0784)/498.0784)* 10^6 = 14.6 ppm

^{1}H and ^{13}C NMR of Succinimidyl 2-azido-4,6-*O*-benzylidene-3-*O*-chloroacetyl-2-deoxy-α-D-galactopyranoside (**15**)

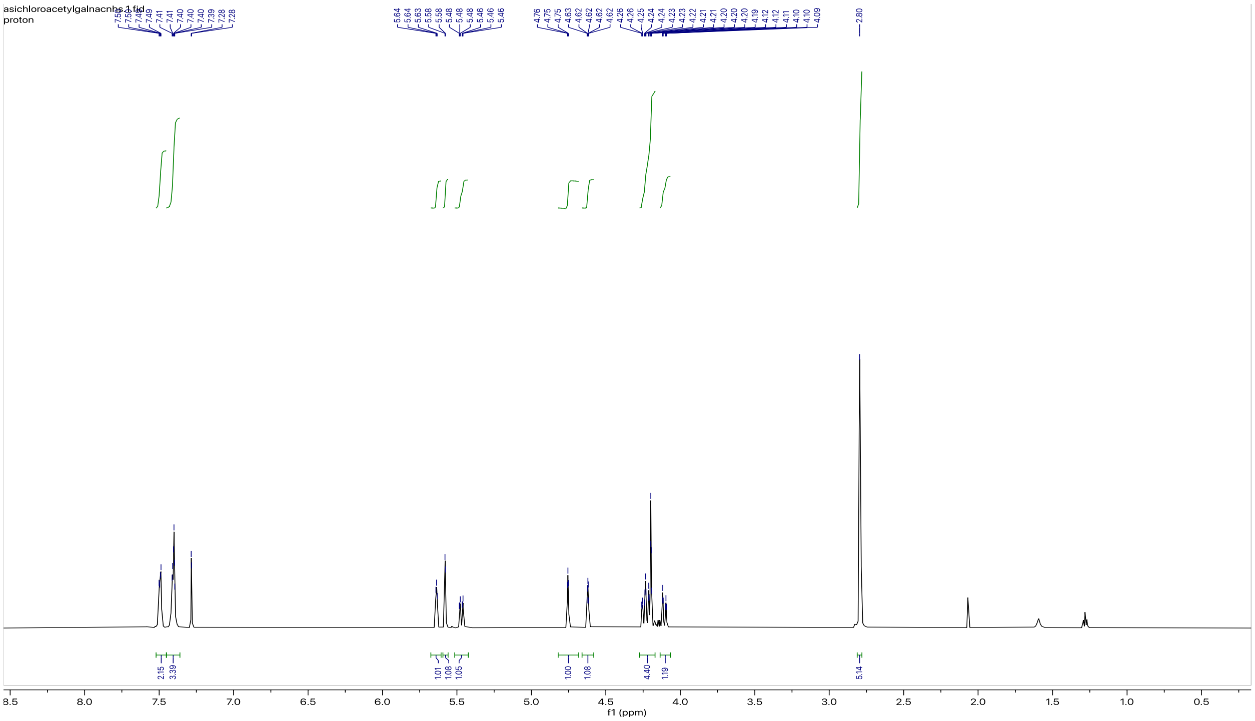

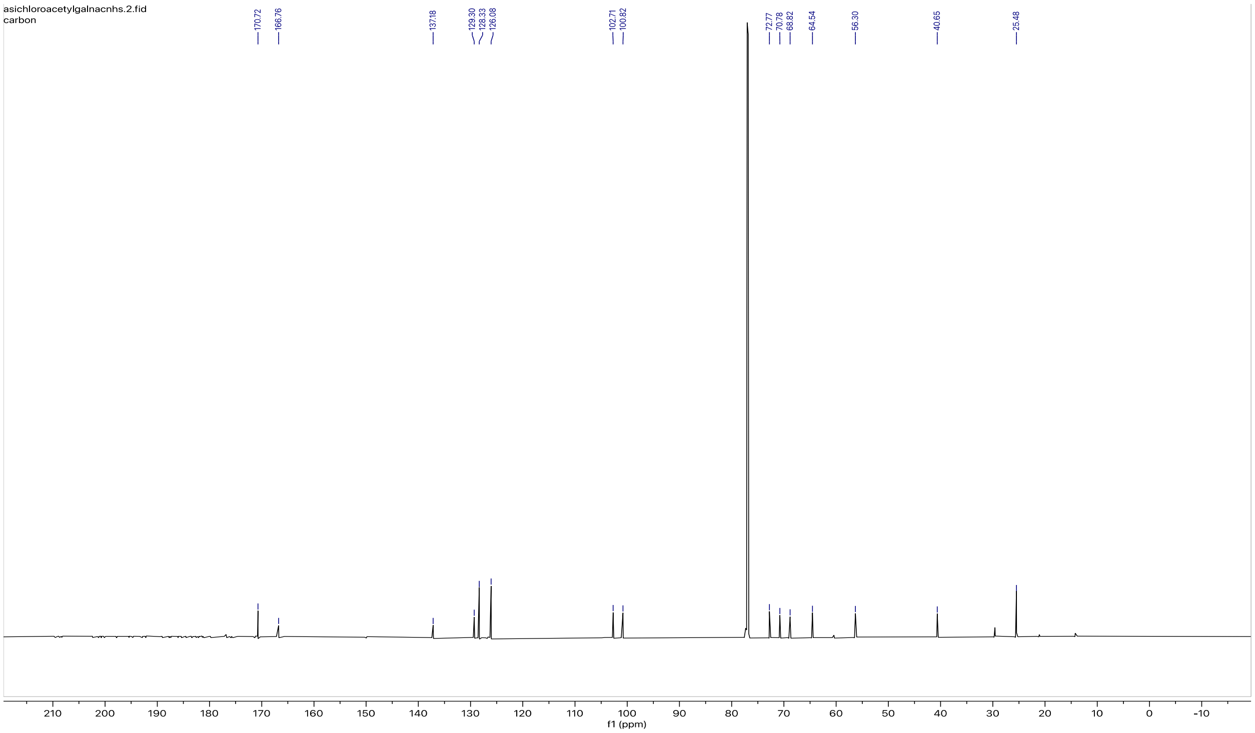

^{1}H and ^{13}C NMR of Succinimidyl 2-azido-4,6-*O*-benzylidene-2-deoxy-α-D-galactopyranoside (**16**)

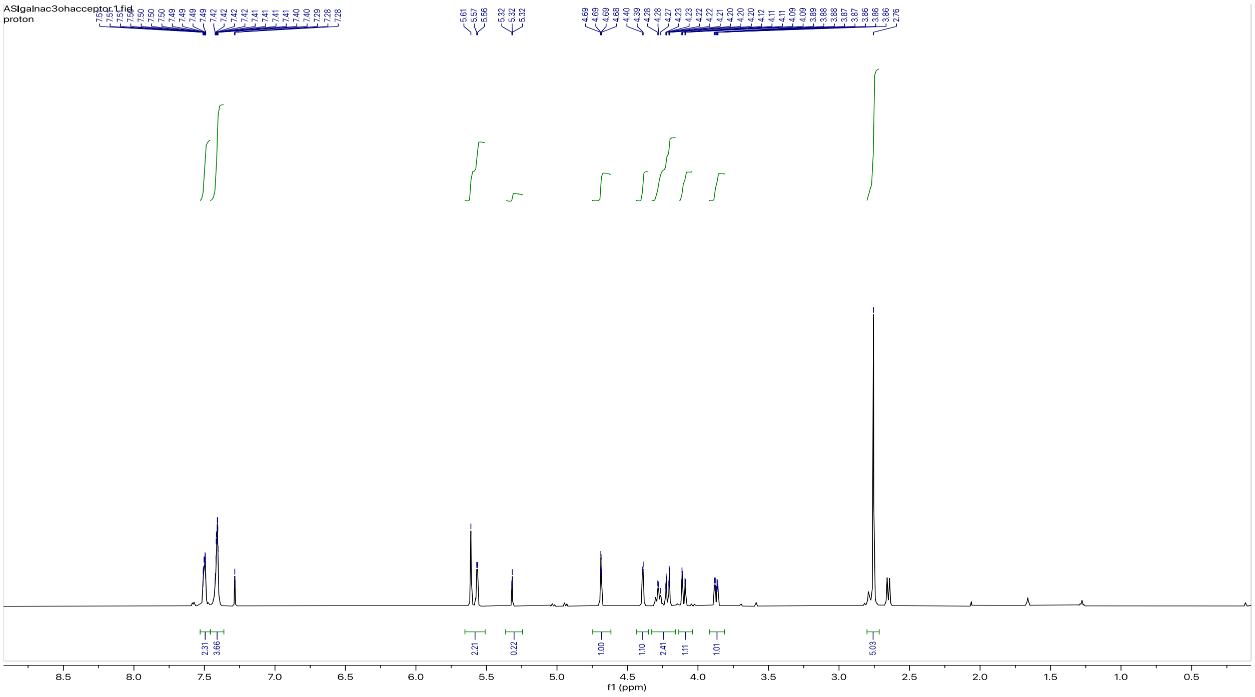

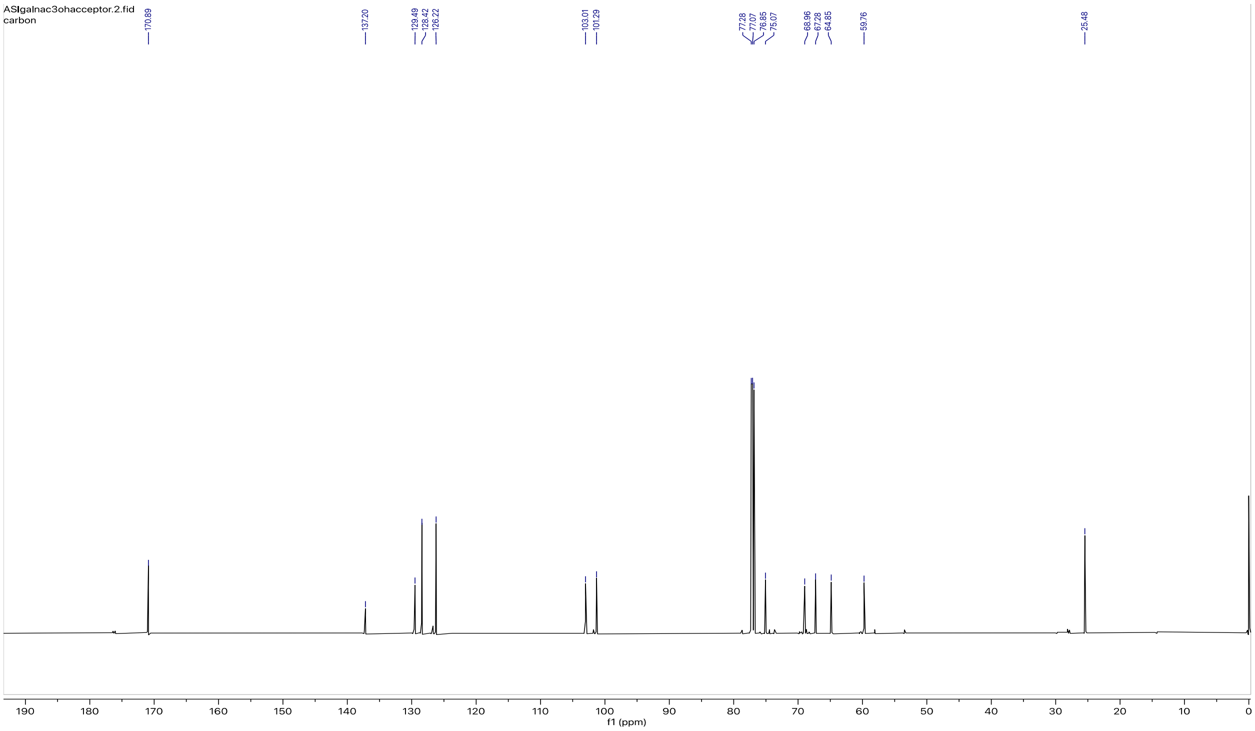

HRMS data of Succinimidyl [2-*O*-benzoyl-4,6-*O*-benzylidene-3-*O*-(p-methoxy)benzyl-β-D-glucopyranosyl]-(1→3)-2-azido-4,6-*O*-benzylidene-2-deoxy-α-D-galactopyranoside (**17**)

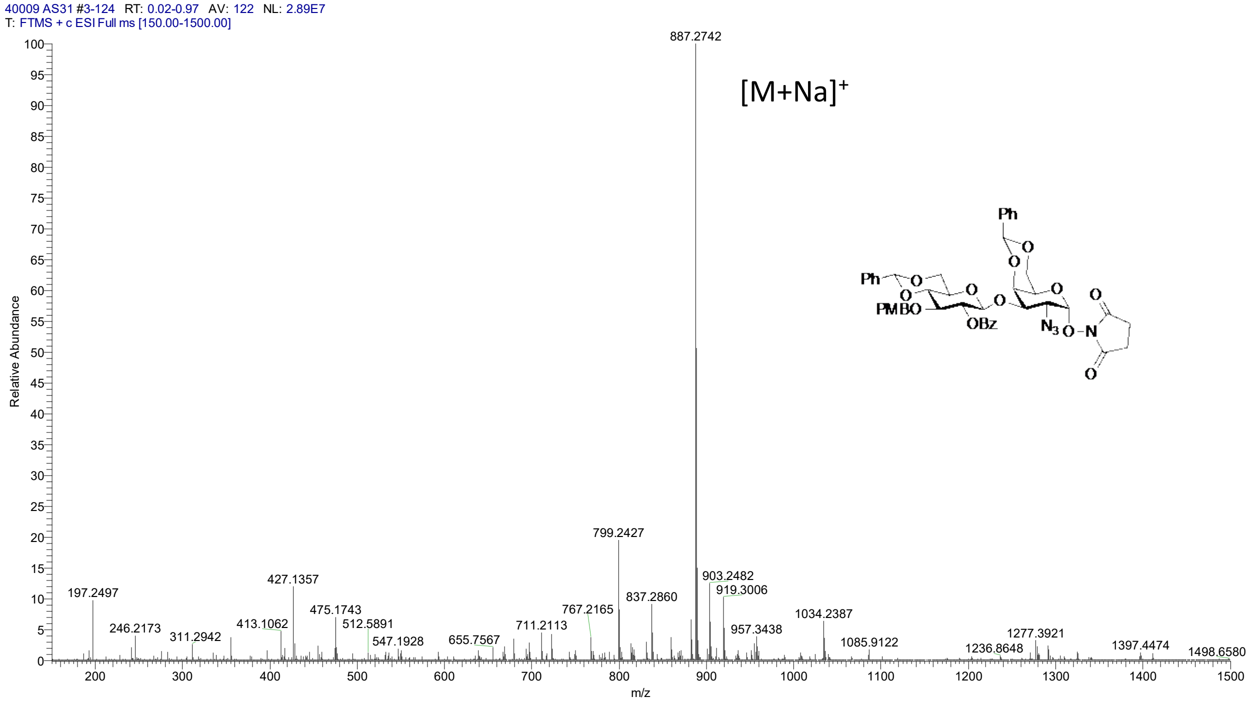

Mass accuracy = (887.2742 – 887.2746)/887.2746)* 10^6 = 0.5 ppm

^{1}H and ^{13}C NMR of Succinimidyl [2-*O*-benzoyl-4,6-*O*-benzylidene-3-*O*-(p-methoxy)benzyl-β-D-glucopyranosyl]-(1→3)-2-azido-4,6-*O*-benzylidene-2-deoxy-α-D-galactopyranoside (**17**)

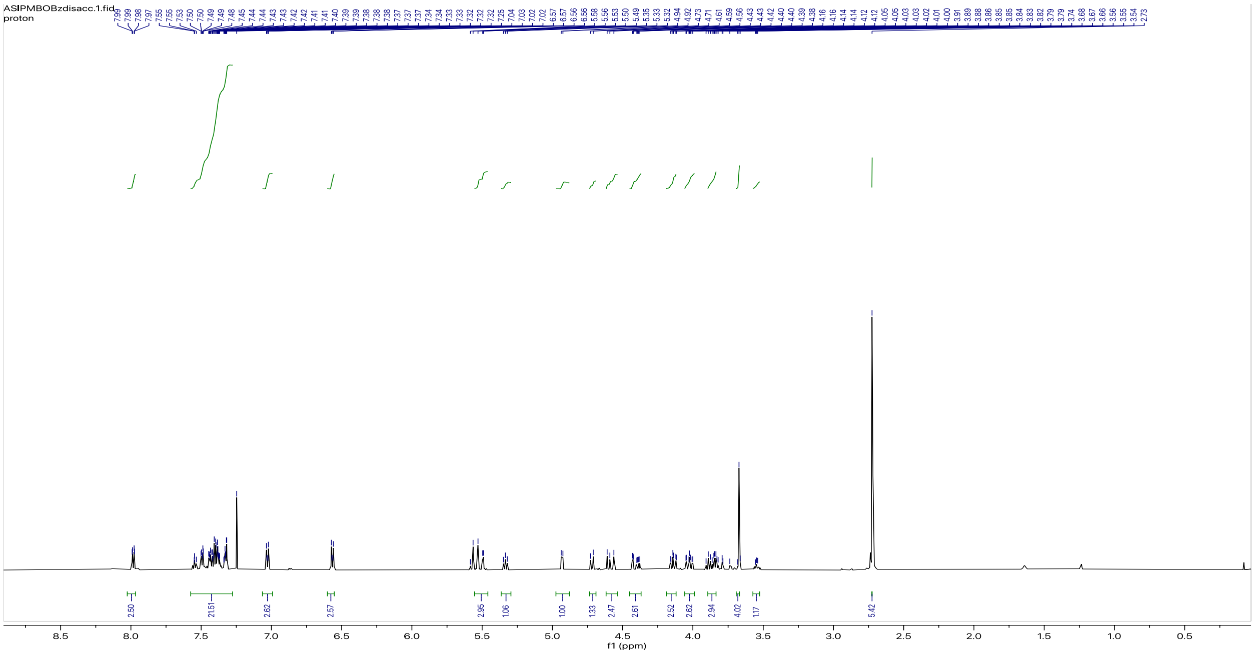

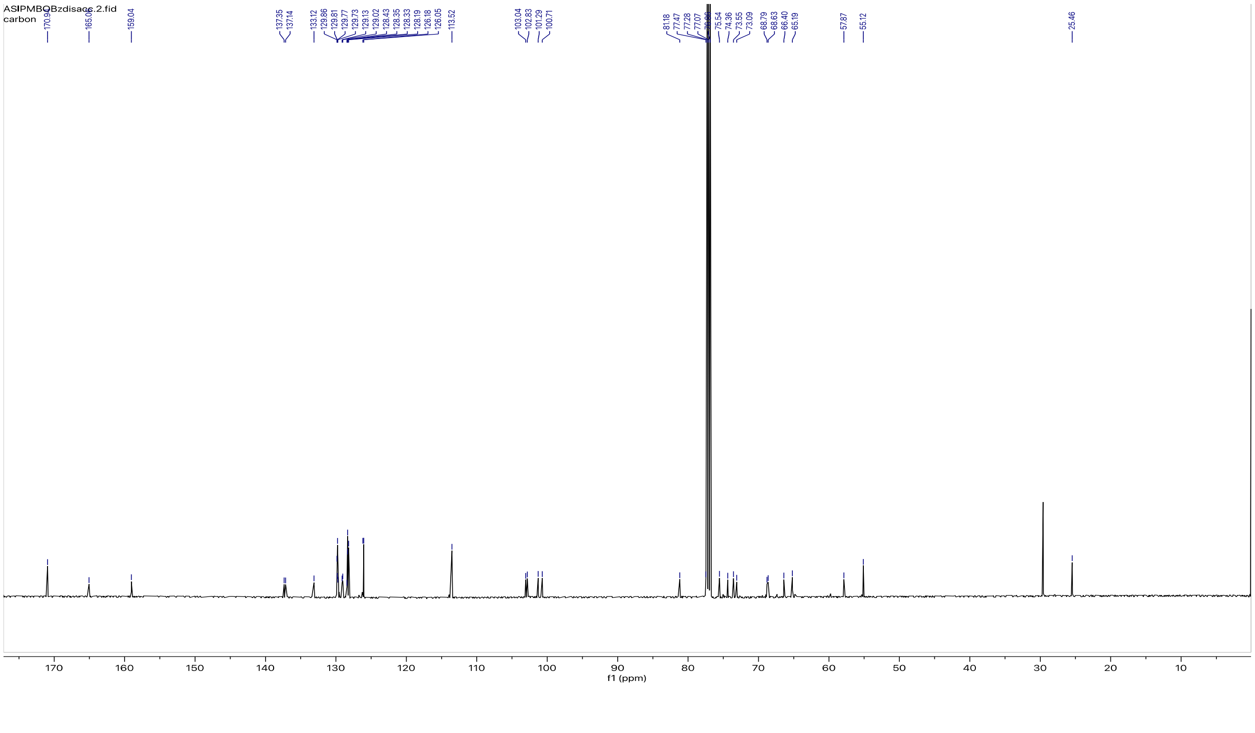

HRMS data of Succinimidyl [2-*O*-benzoyl-4,6-*O*-benzylidene-β-D-glucopyranosyl]-(1→3)-2-azido-4,6-*O*-benzylidene-2-deoxy-α-D-galactopyranoside (**18**)

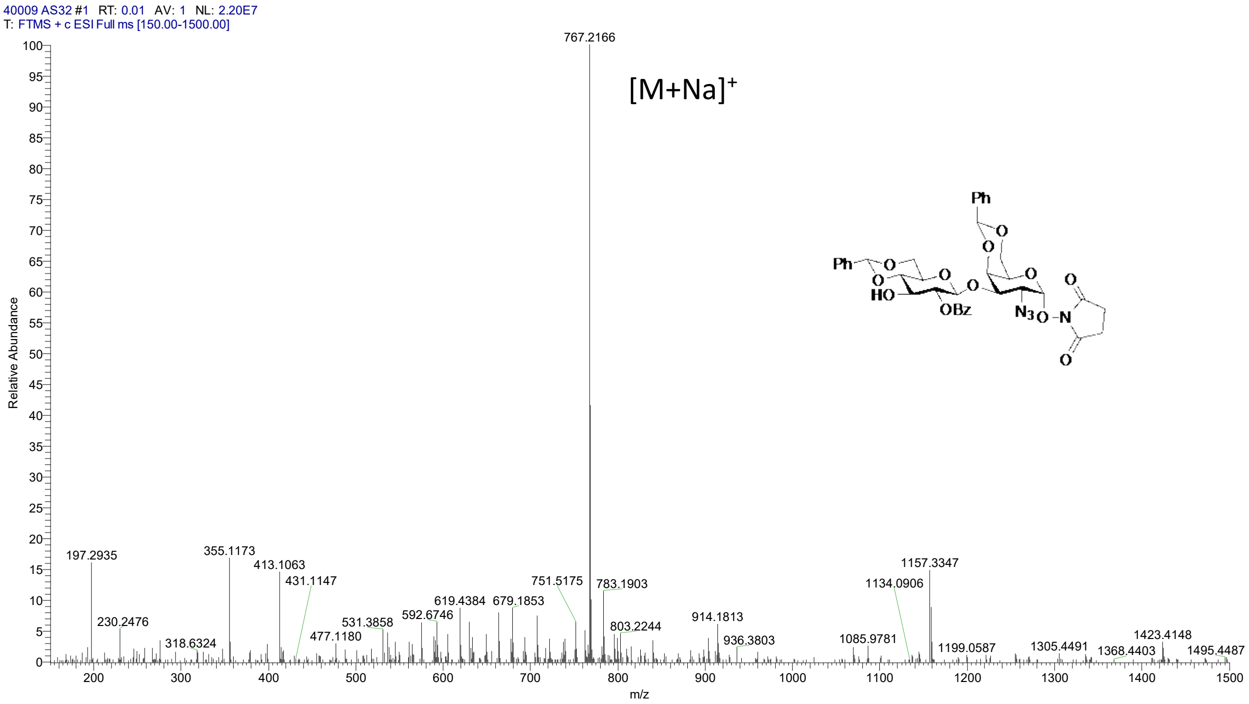

Mass accuracy = (767.2166 – 767.2171)/767.2171)* 10^6 = 0.7 ppm

^{1}H and ^{13}C NMR of Succinimidyl [2-*O*-benzoyl-4,6-*O*-benzylidene-β-D-glucopyranosyl]-(1→3)-2-azido-4,6-*O*-benzylidene-2-deoxy-α-D-galactopyranoside (**18**)

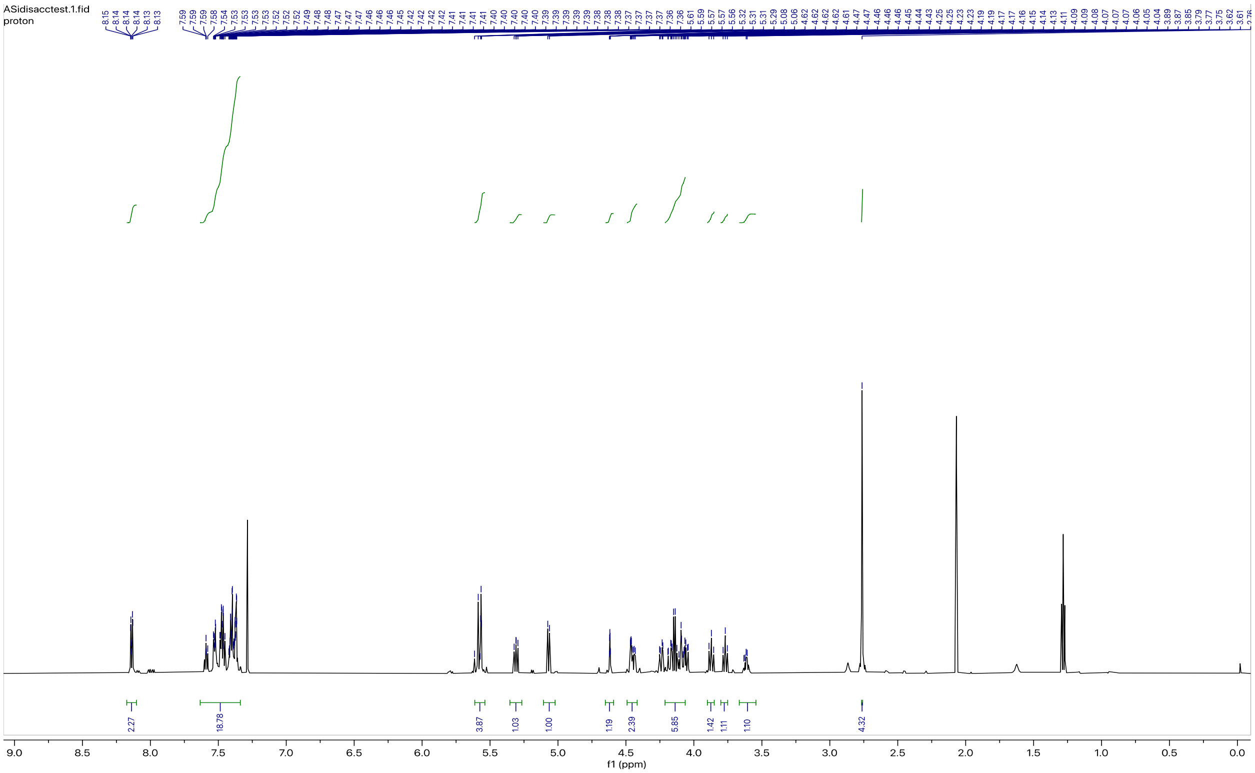

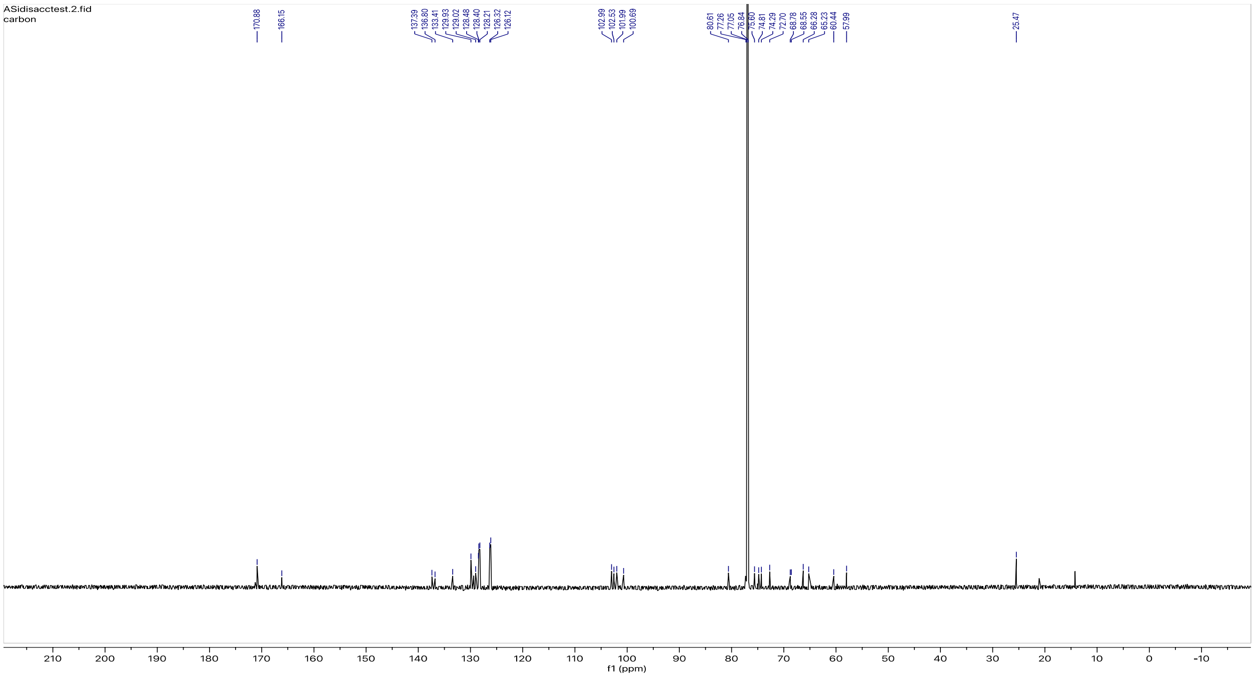

HRMS data of Succinimidyl [2,3,4-tri-*O*-acetyl-α-L-rhamnopyranosyl]-(1→3)-[2-*O*-benzoyl-4,6-*O*-benzylidene-β-D-glucopyranosyl]-(1→3)-2-azido-4,6-*O*-benzylidene-2-deoxy-α-D-galactopyranoside (**19**)

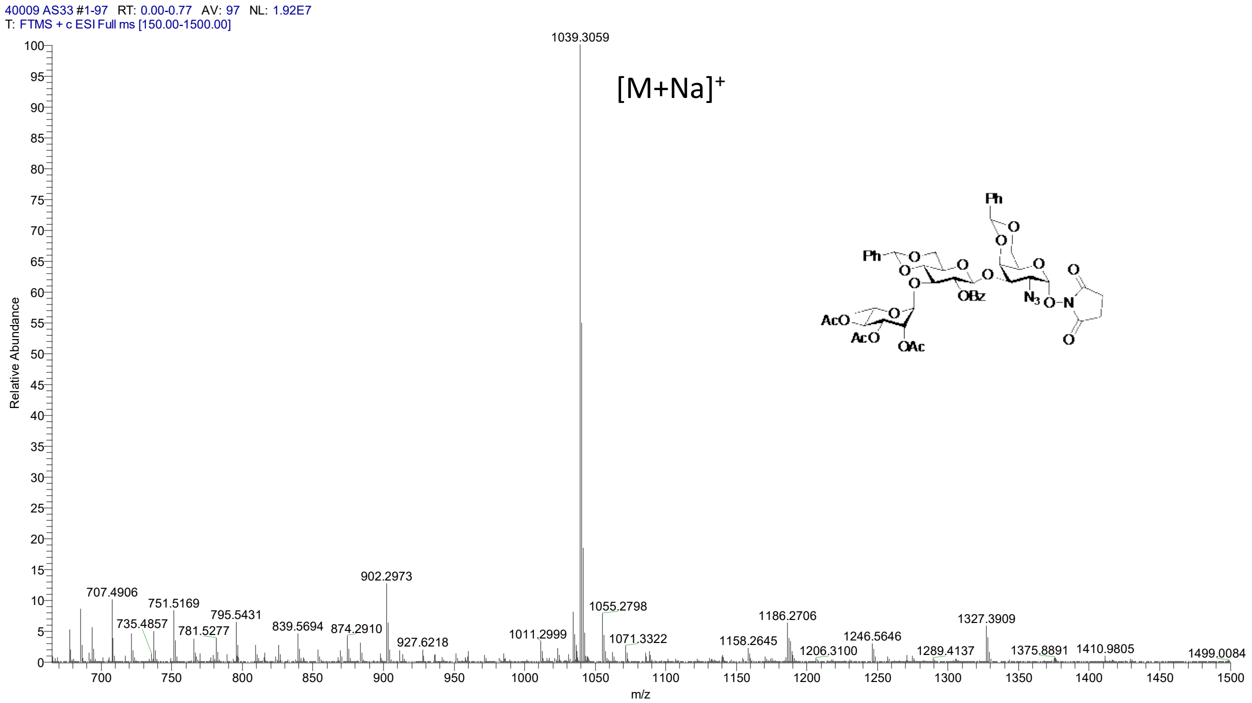

Mass accuracy = (1039.3059 – 1039.3067)/1039.3067)* 10^6 = 0.8 ppm

^{1}H and ^{13}C NMR of Succinimidyl [2,3,4-tri-*O*-acetyl-α-L-rhamnopyranosyl]-(1→3)-[2-*O*-benzoyl-4,6-*O*-benzylidene-β-D-glucopyranosyl]-(1→3)-2-azido-4,6-*O*-benzylidene-2-deoxy-α-D-galactopyranoside (**19**)

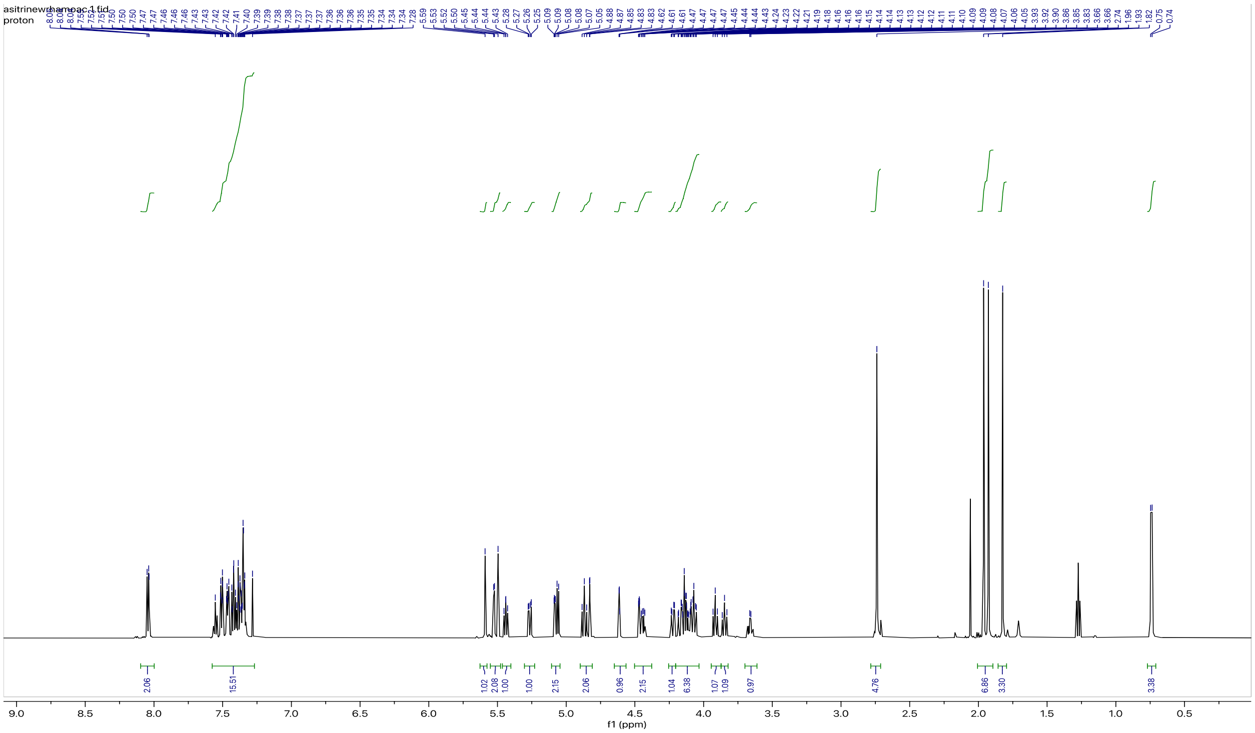

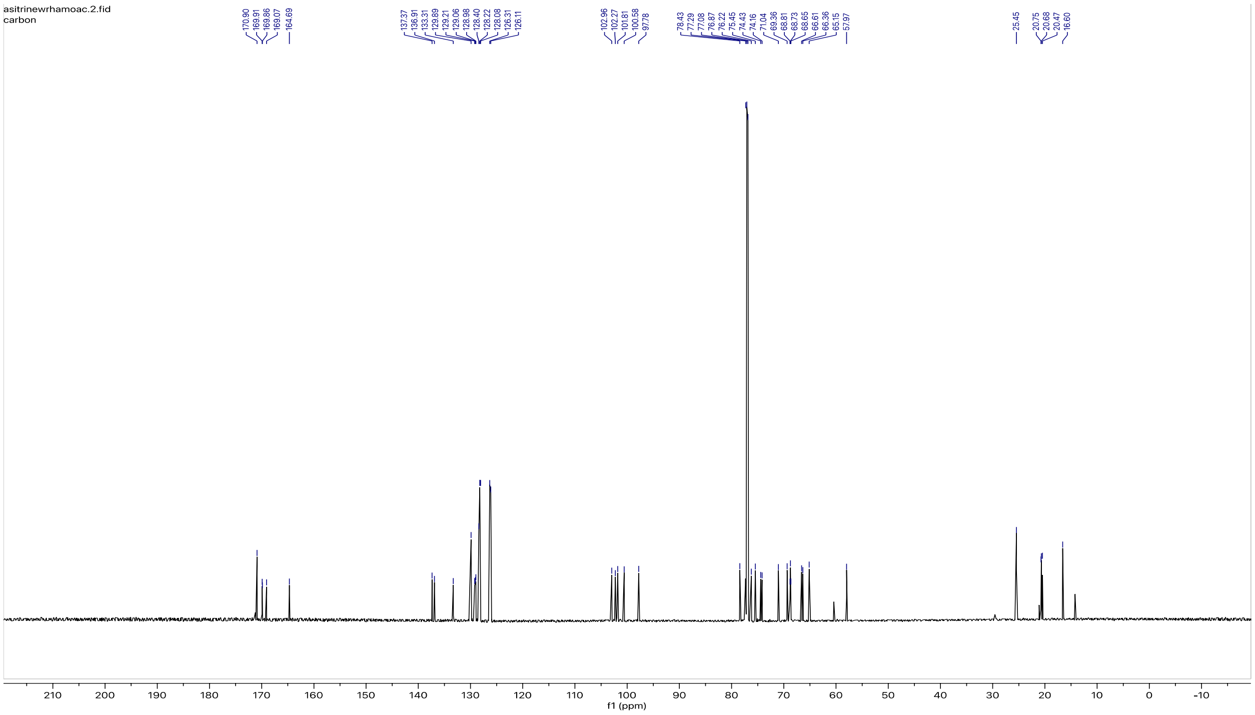

HRMS data of Succinimidyl [2,3,4-tri-*O*-acetyl-α-L-rhamnopyranosyl]-(1→3)-[4,6-*O*-acetyl-2-*O*-benzoyl-β-D-glucopyranosyl]-(1→3)-2-azido-4,6-*O*-acetyl-2-deoxy-α-D-galactopyranoside (**20**)

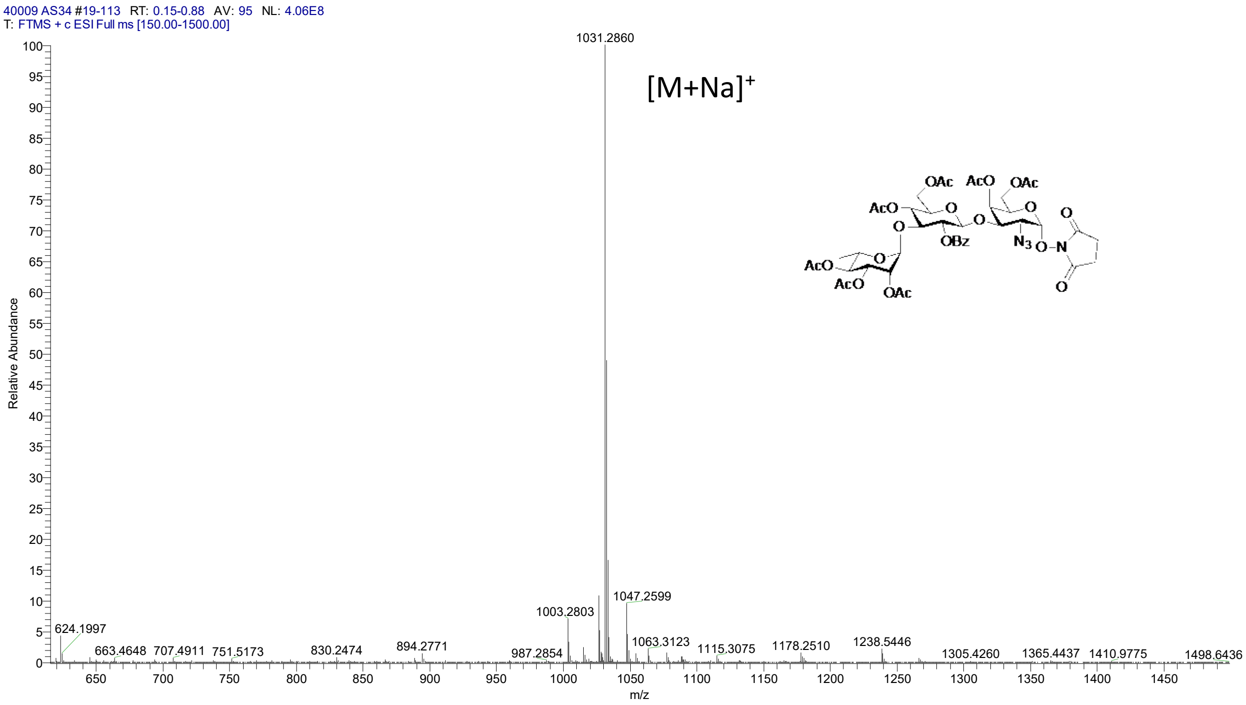

Mass accuracy = (1031.2860 – 1031.2864)/1031.2864)* 10^6 = 0.4 ppm

^{1}H and ^{13}C NMR of Succinimidyl [2,3,4-tri-*O*-acetyl-α-L-rhamnopyranosyl]-(1→3)-[4,6-*O*-acetyl-2-*O*-benzoyl-β-D-glucopyranosyl]-(1→3)-2-azido-4,6-*O*-acetyl-2-deoxy-α-D-galactopyranoside (**20**)

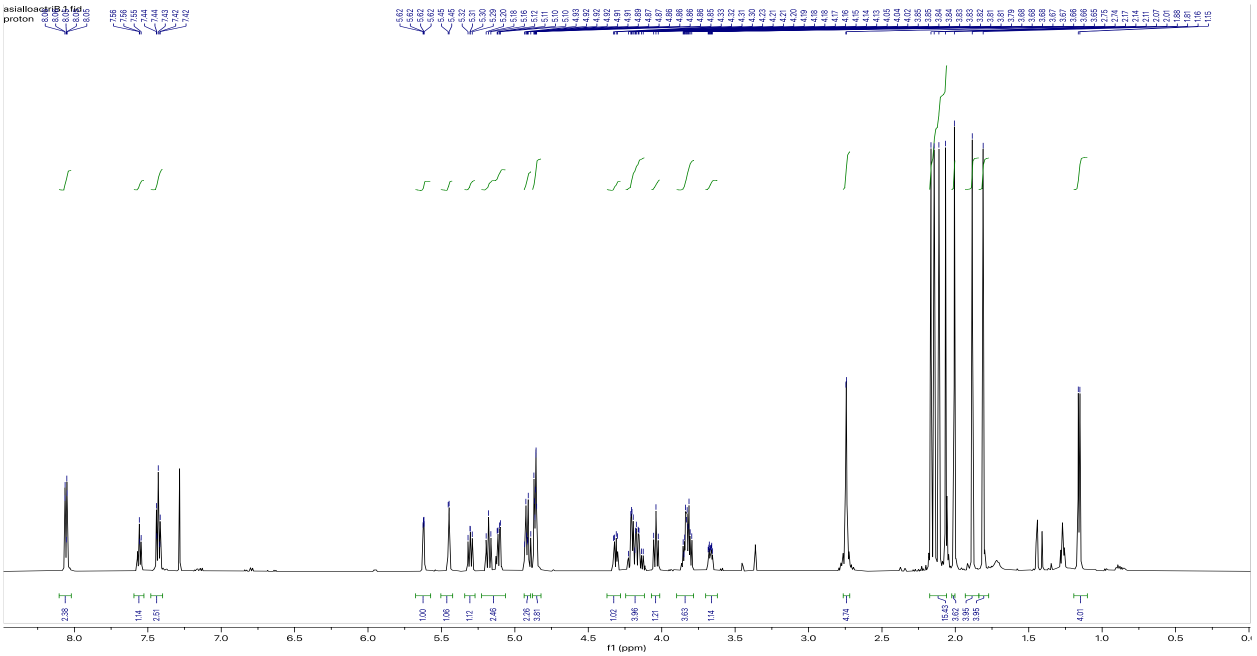

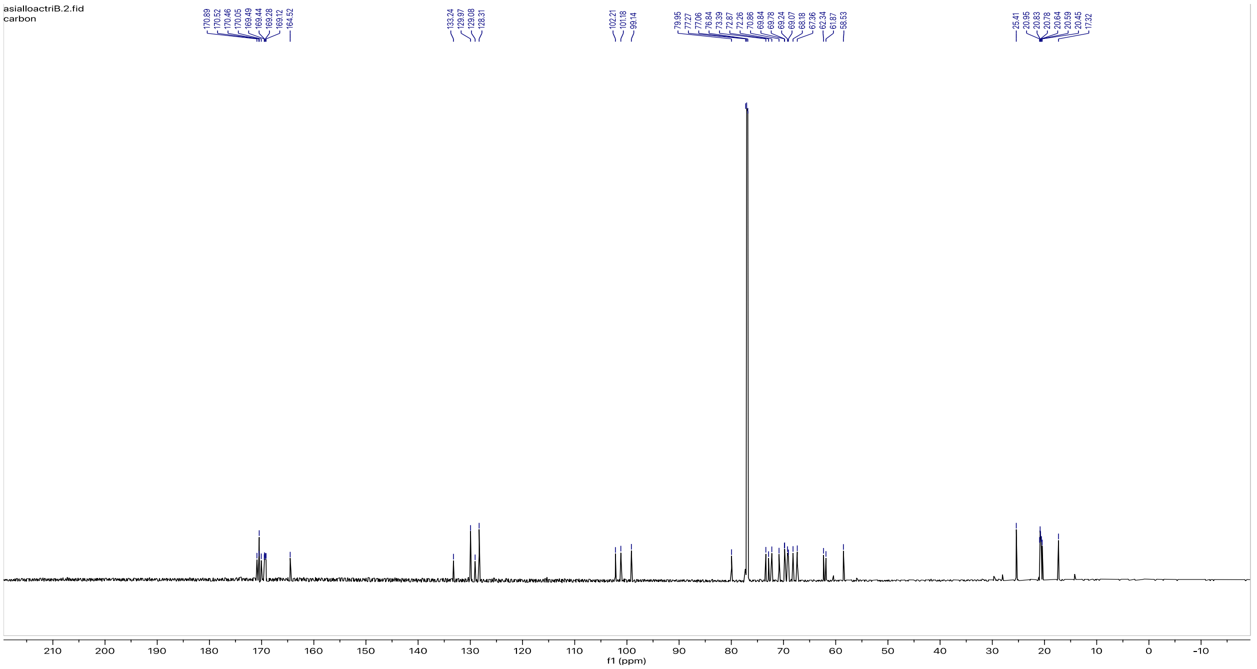

HRMS data of Succinimidyl [2,3,4-tri-*O*-acetyl-α-L-rhamnopyranosyl]-(1→3)-[4,6-*O*-acetyl-2-*O*-benzoyl-β-D-glucopyranosyl]-(1→3)-2-*N*-tertbutyloxycarbonylalanine-4,6-*O*-acetyl-2-deoxy-α-D-galactopyranoside (**21**)

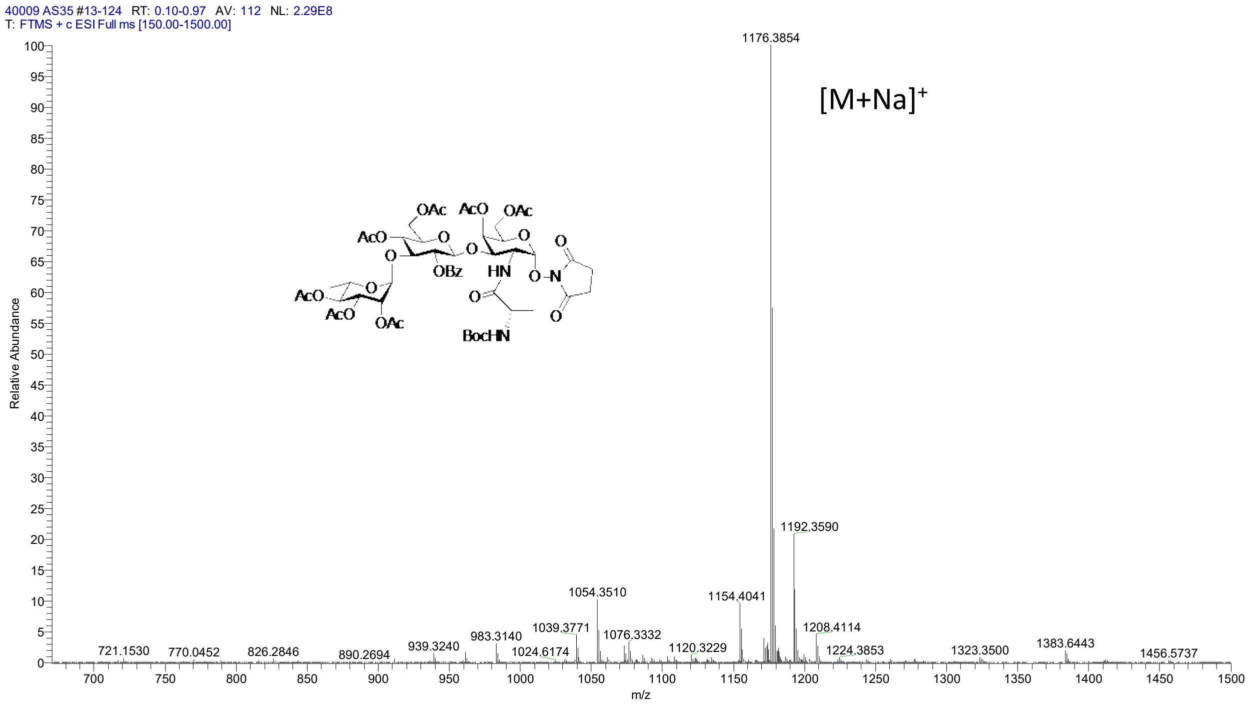

Mass accuracy = (1176.3854 – 1176.3854)/1176.3854)* 10^6 = 0.0 ppm

^{1}H and ^{13}C NMR of Succinimidyl [2,3,4-tri-*O*-acetyl-α-L-rhamnopyranosyl]-(1→3)-[4,6-*O*-acetyl-2-*O*-benzoyl-β-D-glucopyranosyl]-(1→3)-2-*N*-tertbutyloxycarbonylalanine-4,6-*O*-acetyl-2-deoxy-α-D-galactopyranoside (**21**)

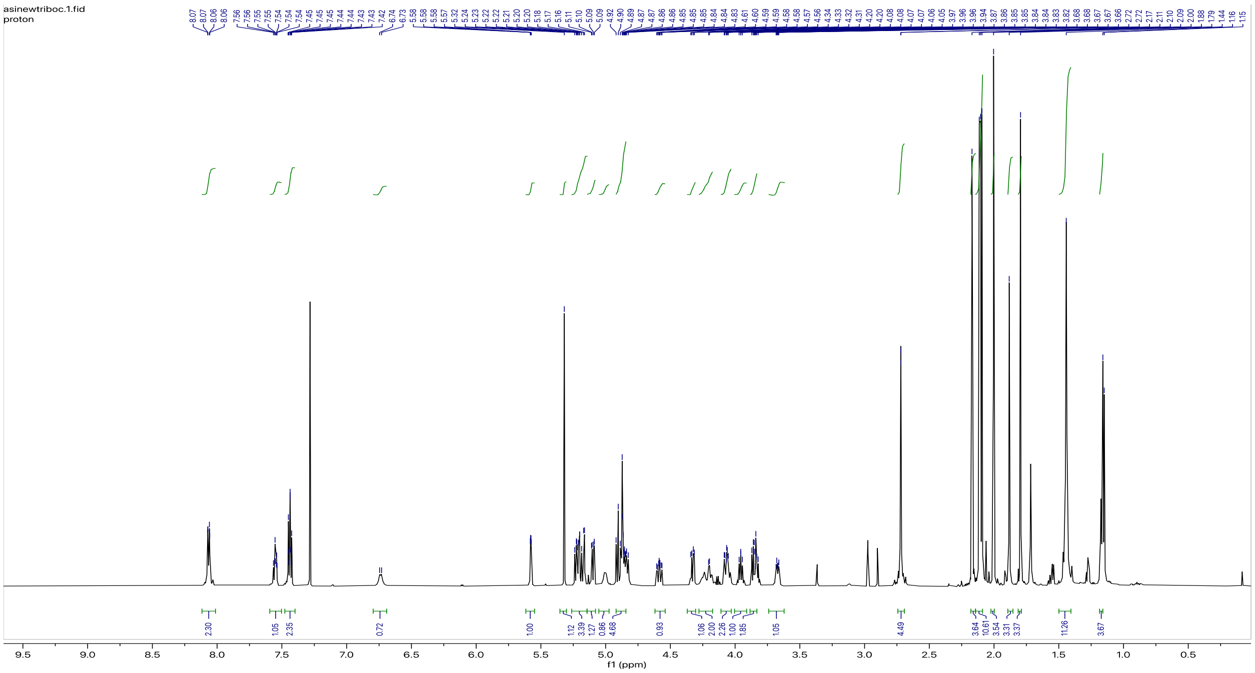

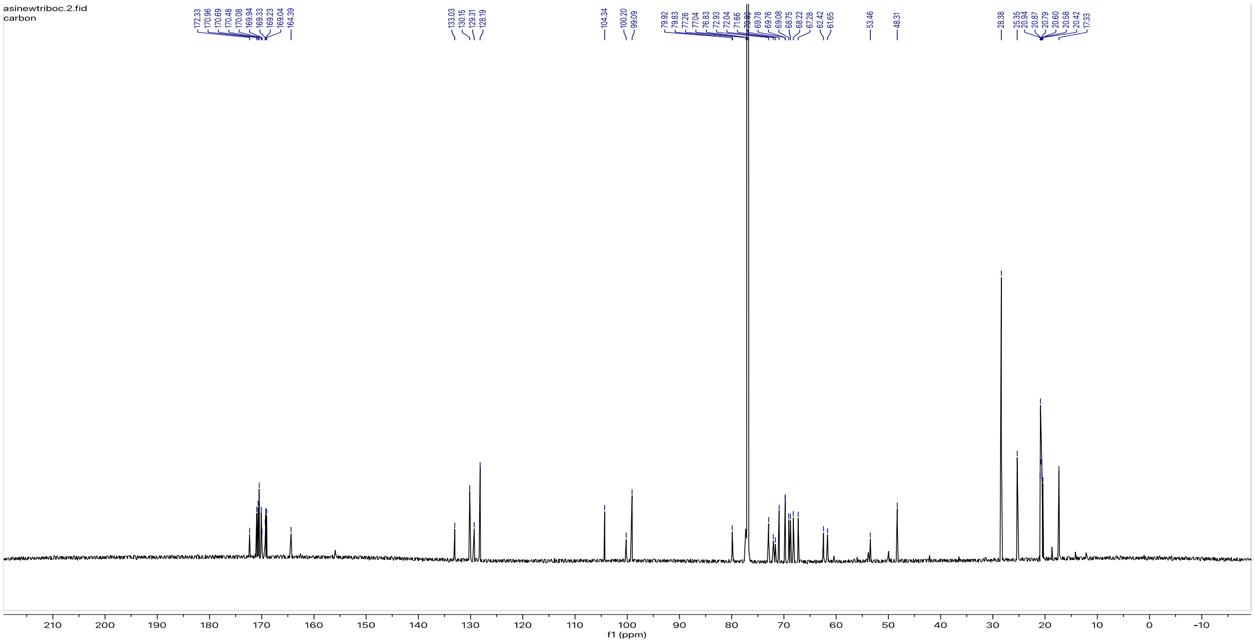

HRMS data of Aminooxy [α-L-rhamnopyranosyl]-(1→3)-[β-D-glucopyranosyl]-(1→3)-2-*N*-alanine-2-deoxy-α-D-galactopyranoside (**4**)

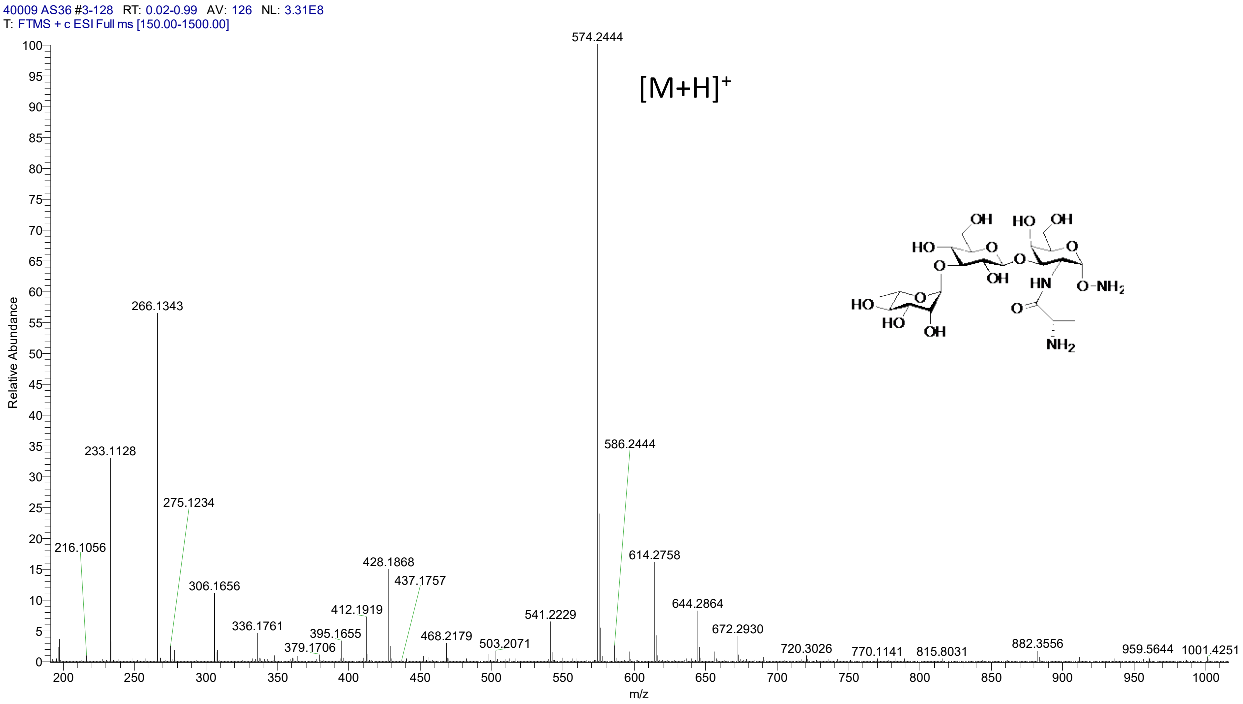

Mass accuracy = (574.2444 – 574.2453)/574.2453)* 10^6 = 1.6 ppm

^{1}H and ^{13}C NMR of Aminooxy [α-L-rhamnopyranosyl]-(1→3)-[β-D-glucopyranosyl]-(1→3)-2-*N*-alanine-2-deoxy-α-D-galactopyranoside (**4**)

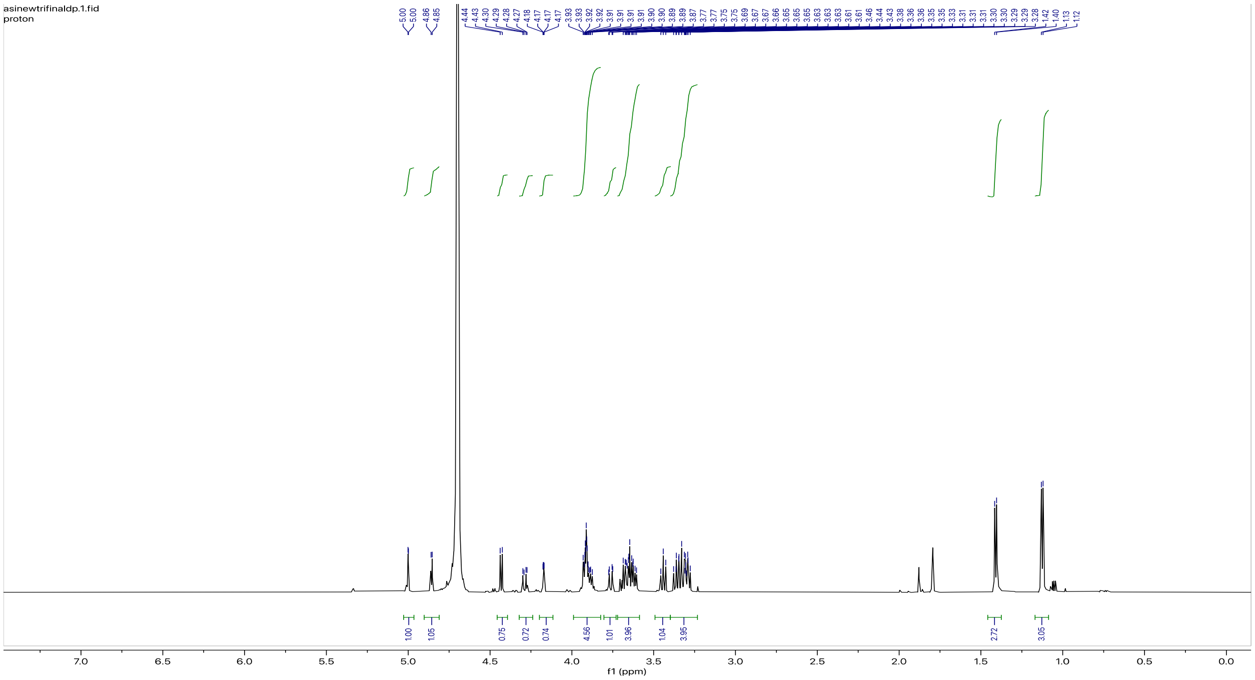

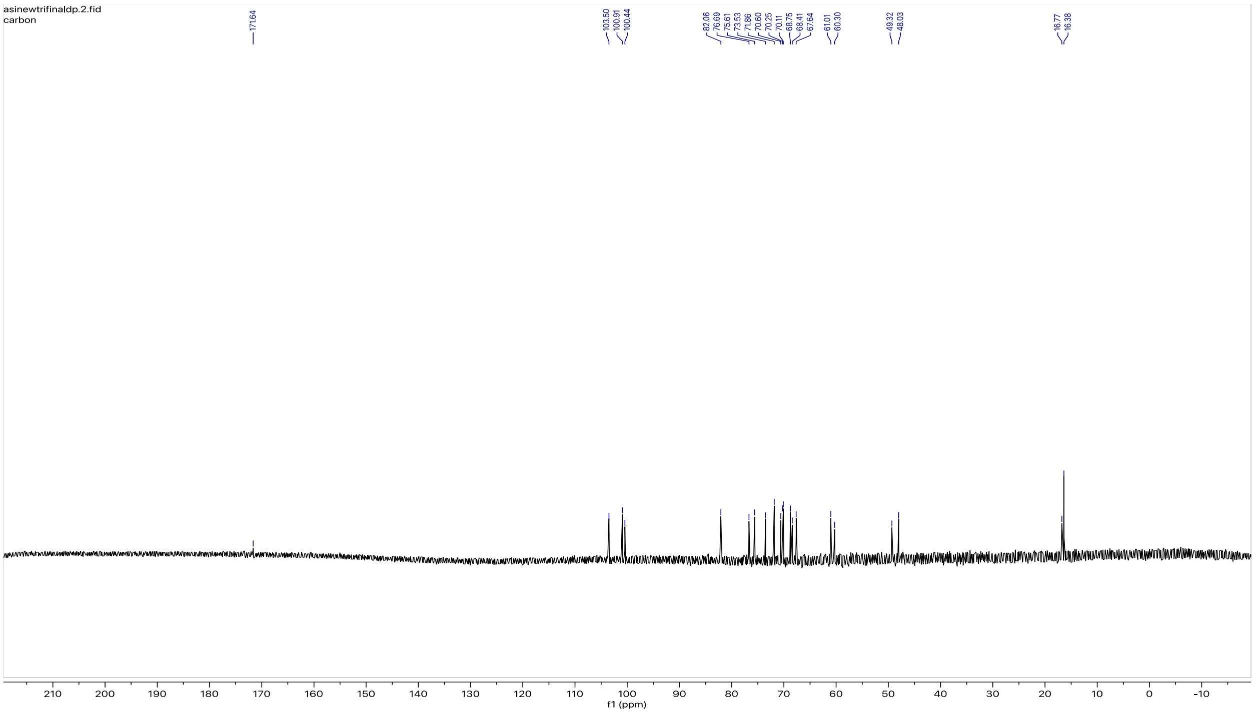

Supplement: Supplementary file 1 [file DataSheet1.PDF]
